# Nanoconfinement-cooperated catalysis enables rapid tandem redox kinetics for wide-temperature lithium-sulfur batteries

**DOI:** 10.1126/sciadv.aej0353

**Published:** 2026-10-14

**Authors:** Tianli Li, Tao Chen, Pu Hu, Congcong Cai, Juntao Wang, Chaoqun Shang, Liwei Xiong, Yajie Li, Hanxing Liu, Jian Wang, Yaobing Wang

**Affiliations:** ^1^ State Key Laboratory of Green and Efficient Development of Phosphorus Resources, Hubei Key Laboratory of Plasma Chemistry and Advanced Materials, School of Materials Science and Engineering, Wuhan Institute of Technology, Wuhan, 430205, China.; ^2^ State Key Laboratory of Advanced Technology for Materials Synthesis and Processing, School of Material Science and Engineering, International School of Material Science and Engineering, Wuhan University of Technology, Wuhan 430070, China.; ^3^ State Key Laboratory of Materials for Advanced Nuclear Energy & School of Materials Science and Engineering, Shanghai University, Shanghai, 200444, China.; ^4^ Helmholtz Institute Ulm (HIU) D89081 Ulm, Germany Karlsruhe Institute of Technology (KIT) D76021 Karlsruhe, Germany.; ^5^ State Key Laboratory of Structural Chemistry, Fujian Institute of Research on the Structure of Matter, Chinese Academy of Sciences, Fuzhou 350002, Fujian, P. R. China.

## Abstract

The slow kinetics of sulfur cathodes caused by the high energy barriers of Li^+^ desolvation stem primarily from the structure of the inner Helmholtz plane (IHP). Herein, a synergistic strategy combining electrochemical tandem catalysis with spatial nanoconfinement is proposed to regulate the Li(solvent)*_x_*^+^ dynamics. As a proof-of-concept, the porous covalent organic framework (denoted as TPBD) decorated MXene is in situ constructed, acting as a tandem kinetic promoter. The theoretical simulations and in situ Raman reveal that the porous TPBD layer confines the bulky Li(solvent)*_x_*^+^ species and sieves the spatial structure with accelerated Li^+^ diffusion kinetics. Meanwhile, MXene serves as a negatively charged substrate that helps disrupt anion-solvent interactions and reconstruct the solvation structure at the IHP, thereby enhancing Li^+^ kinetics and sulfur redox reversibility. Encouragingly, the assembled cells demonstrate a superior Li^+^ transference number (0.77), long-term cycling lifespan up to 1000 cycles, as well as excellent areal capacity (>6 mAh cm^−2^) at 0°C and superior rate capability at 50°C under a high sulfur loading of 7.51 mg cm^−2^. The as-prepared pouch cell remains stable for over 100 cycles, validating this novel integration of catalysis and nanoconfinement for tandem kinetics in Li-S batteries.

## INTRODUCTION

Lithium-sulfur (Li-S) batteries are regarded as promising high-energy battery systems due to their extremely high theoretical energy density (*1*, *2*). However, the sluggish tandem kinetics of the sulfur cathode, caused by the high energy barrier for Li^+^ desolvation and polysulfide shuttling, contribute to severe capacity fading, limiting their further development (*3*, *4*). To tackle these challenges, Pol and co-workers have made important advances in polysulfide management and interfacial engineering. For instance, they demonstrated that functionalized separator coatings (*5*, *6*) and polar carbon hosts (*7*) can immobilize polysulfides and enhance electrochemical kinetics. While these strategies are effective at suppressing polysulfide shuttling at the separator and cathode interfaces, further modulating the intrinsic Li^+^ desolvation barrier at the inner Helmholtz plane (IHP) is essential to fundamentally accelerate the redox kinetics in Li-S batteries. Concurrently, recent efforts have focused on designing functional catalysts with delocalized electrons to achieve strong electronic/ionic coupling with sulfur species, thereby reducing the conversion energy barrier and improving redox reversibility (*8*–*10*). These studies are generally based on surface interactions between electrocatalysts and sulfur. However, the conversion of sulfur species involves the mass transport of multiple species and lithium ions (Li^+^), a process intrinsically linked to Li^+^ migration and featuring sequential reactions in the sulfur cathode (*11*–*13*). Therefore, driving efficient Li^+^ migration across the IHP is critical for achieving high energy density and long cycle life.

It is well established that in the electrolyte, the Li^+^ preferentially coordinates with surrounding solvent molecules, forming the Li(solvent)*_x_*^+^ solvation shell. This leads to solvent-separated ion pairs (SSIPs) with a large hydrodynamic radius (*14*, *15*). The resultant bulky solvated clusters impede Li^+^ mobility due to pronounced steric hindrance at the interface. Recently, the anion chemistry of mixing TFSI^−^ and FSI^−^ can modulate the anion-solvent interactions in the Li^+^ solvation sheath, promoting the formation of contact ion pairs (CIPs) or ion aggregates (AGGs), and consequently lowering the desolvation barrier (*16*, *17*). However, such bulk electrolyte modification inevitably increases viscosity, which significantly compromises ionic conductivity. This trade-off calls for a more localized reconstruction of the Li^+^ solvation structure. Alternatively, the porous metal-organic framework has been introduced to sieve large Li(solvent)*_x_*^+^ species and boost the transport across the IHP. However, this porous sieving effect strongly relies on electric fields and lacks sufficient driving force to further boost the diffusion. Given that sulfur species conversion always occurs at the electrode-electrolyte interface (*18*), it is necessary to reconstruct the IHP to enable fast desolvation within this confined region and provide abundant active sites, thereby significantly enhancing the cycling durability and practical utilization of sulfur species.

Distinct from conventional electrolyte engineering (*19*, *20*) and prior COF/MXene studies that primarily rely on ion sieving or polysulfide blocking at the separator interface (*21*–*23*), our work centers on the microscopic IHP where sluggish Li^+^ desolvation occurs. We propose a synergistic strategy that precisely couples spatial nanoconfinement with electrochemical tandem catalysis. This strategy dynamically reconstructs the Li(solvent)*_x_*^+^ solvation structure and enables stepwise polysulfide catalysis at the heterointerface, tackling both sluggish Li^+^ migration and polysulfide shuttle effect. As a proof-of-concept, the porous covalent organic framework (denoted as TPBD) decorated MXene composite is in situ constructed via a Schiff-base polycondensation reaction between benzene-1,3,5-tricarboxaldehyde and 4,4′,4″-(1,3,5-triazine-2,4,6-triyl) trianiline directly on MXene nanosheets, acting as a coupled kinetic promoter. The TPBD layer strips the outer solvation sheath, and the partially desolvated Li^+^ then reaches the MXene surface for subsequent anion-solvent bond cleavage. Density functional theory (DFT) calculations, in situ Raman spectroscopy, and time-of-flight secondary ion mass spectrometry (TOF-SIMS) analyses reveal that within the Helmholtz interface, the porous TPBD layer confines the large Li(solvent)*_x_*^+^ species and provides spatial sieving with accelerated Li^+^ diffusion kinetics. Meanwhile, the abundant catalytic sites on MXene serve as negatively charged substrates that help to disrupt the anion-solvent interactions, thereby reconstructing the solvation structure at the IHP and enhancing Li^+^ kinetics for sulfur redox reversibility. Consequently, with the synergistic effect of TPBD/MXene, the optimal cells maintain an areal capacity of 8.16 mAh cm^−2^ after 70 cycles at 9.72 mg cm^−2^, surpassing the performance benchmarks of commercial lithium-ion batteries. Even at a low temperature of 0°C, the cell delivers a high areal capacity of 6.53 mAh cm^−2^ (781.09 mAh g^−1^) after 259 cycles at 8.36 mg cm^−2^, along with excellent rate capability at 50°C under a high sulfur loading of 7.51 mg cm^−2^. Moreover, the assembled pouch cell achieves a capacity retention of over 85% after 100 cycles, underscoring the superiority of synergistic catalysis and spatial nanoconfinement in enabling rapid Li^+^ transport for high-performance Li-S batteries.

## RESULTS

### Mechanism illustration and material characterizations

Various solvents coordinate well with Li^+^ to form bulky clusters, thereby blocking ion diffusion and suppressing subsequent reactions. Meanwhile, maintaining a Li^+^-abundant state in the IHP during the discharge process is also challenging. As illustrated in Fig. 1A, the tailored interfacial chemistry of TPBD/MXene heterostructures facilitates dynamic reconstruction of the Li(solvent)*_x_*^+^ structure via the catalytic sites and pore sizes. Besides, the strong chemisorption of anions onto TPBD/MXene surfaces locally enriches the interface with abundant CIPs and AGGs. This Stern-layer restructuring displaces solvent molecules from the primary Li^+^ solvation shell, weakening solvent-ion interactions and forming an ion-conductive pathway, thereby enabling rapid Li^+^ desolvation kinetics. In contrast, the pure TPBD lacks sufficient catalytic sites, failing to effectively exclude solvents from the IHP or enrich CIPs/AGGs, which contributes to retarded Li^+^ desolvation kinetics (fig. S1). Building upon this interfacial acceleration, Fig. 1B depicts the holistic working mechanism of the Li-S battery. The TPBD/MXene heterostructure, featuring a donor-acceptor induced internal electric field, translates optimized desolvation kinetics into superior device performance by: (i) providing dual-conductive networks to lower reaction barriers; (ii) physically and chemically trapping polysulfides to curb the shuttle effect; and (iii) homogenizing Li^+^ flux to suppress dendrite growth. As a proof of concept, 4,4′,4″-(1,3,5-triazine-2,4,6-triyl) trianiline (TP) anchored onto MXene was designed through interfacial interactions. Further, benzene-1,3,5-tricarboxaldehyde (BD) then reacted with the amino groups of TP via Schiff-base polycondensation (fig. S2), yielding the porous TPBD/MXene heterostructure with enhanced structural integrity. The product was further characterized by scanning electron microscopy (SEM) (figs. S3 and S4).

**Fig. 1.**
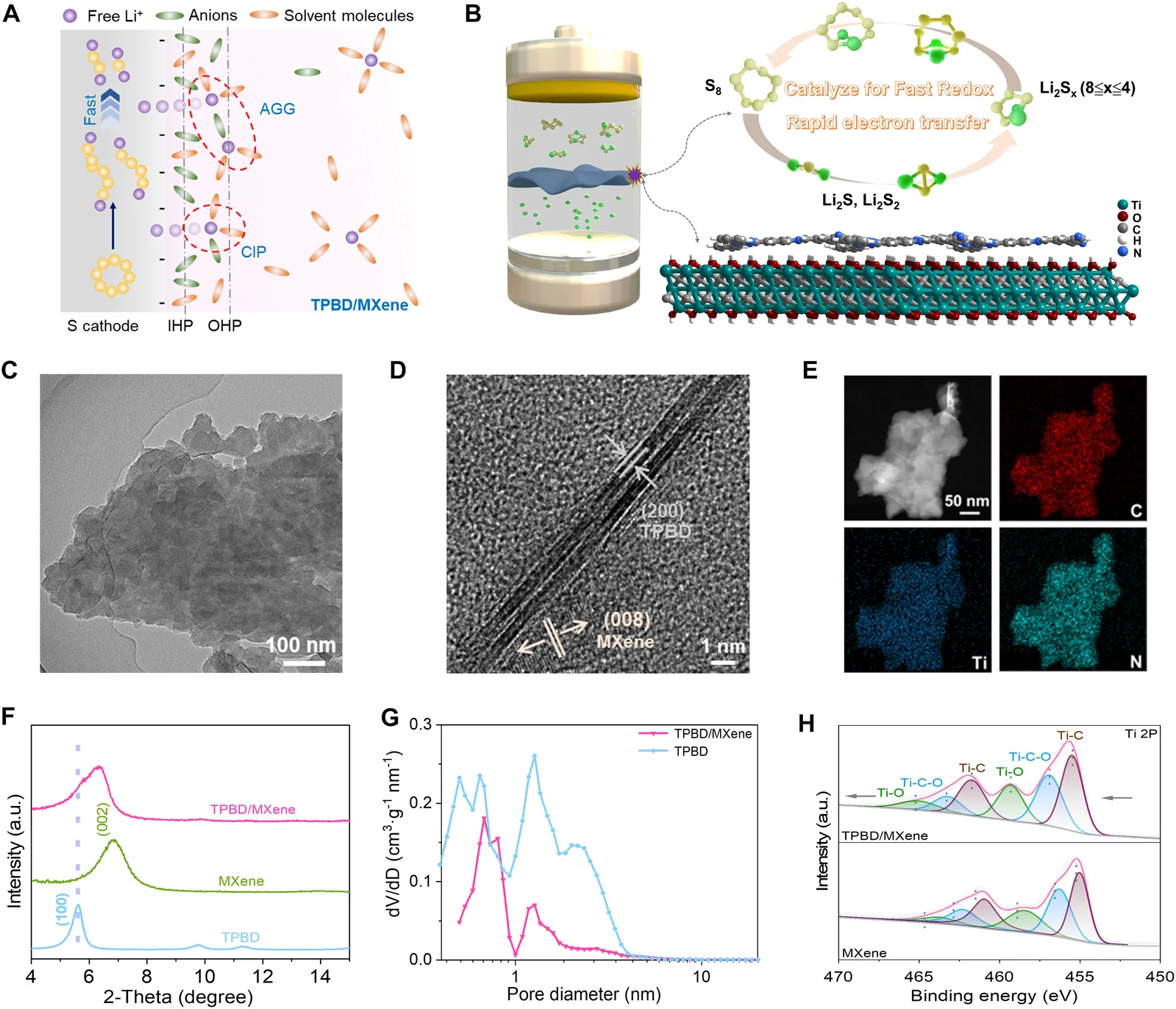
Schematic illustration and structural characterization. **(A)** Schematic of Li^+^ desolvation shell evolution. **(B)** Schematic of accelerated bidirectional sulfur conversion enabled by TPBD/MXene. **(C)** TEM image. **(D)** HRTEM image. **(E)** HAADF-STEM image and elemental mappings. **(F)** XRD patterns. **(G)** Pore size distribution. **(H)** Ti 2p XPS spectra of the as-synthesized materials.

Accordingly, transmission electron microscopy (TEM) analysis elucidates the microstructure morphology of TPBD/MXene (Fig. 1C). In contrast to pure TPBD (fig. S5), TPBD is uniformly distributed on the MXene surface without severe agglomeration. The high-resolution TEM (HRTEM) image (Fig. 1D) displays two distinct interplanar spacings of 1.446 nm and 0.213 nm, corresponding to the (200) plane of TPBD and the (008) plane of MXene, respectively. Notably, the pronounced boundary indicates the two phases of TPBD and MXene, suggesting the construction of the TPBD/MXene heterostructure. Besides, elemental mappings (Fig. 1E) confirm the uniform distribution of Ti, C, and N elements throughout the TPBD/MXene heterostructure. The crystallographic structure evolution was investigated via X-ray diffraction (XRD) (Fig. 1F). The characteristic TPBD/MXene peak shifts to a higher angle (rightward) compared to pristine TPBD, and the prominent peak of the heterostructure appears between the (002) peak of MXene and the (100) peak of TPBD, substantiating the successful integration and strong interaction between the two phases. As elucidated in Raman spectra (fig. S6), the heterostructure exhibits two pronounced peaks at 203 cm^−1^ and 731 cm^−1^, assigned to the A_1g_ (Ti, O, C) and A_1g_ (C) modes of MXene, respectively. The peaks at 1589 cm^−1^ and 1629 cm^−1^ correspond to the C=C and C=N vibrational modes of TPBD (*24*, *25*). As indicated in Fourier Transform Infrared (FTIR) Spectroscopy (fig. S7), the peak at 1624 cm^−1^ (C=N) in the heterostructure corresponds to the imine bonds in the TPBD skeleton, confirming that the Schiff-base polycondensation between aldehyde and amino groups has occurred (*26*, *27*).

As exhibited in Fig. 1G and fig. S8, the Brunauer-Emmett-Teller (BET) surface area of TPBD/MXene decreased to 292 m^2^ g^−1^ compared to raw TPBD (1123 m^2^ g^−1^). This reduction is attributed to the dense coverage of TPBD on the MXene surface. The resulting microporous TPBD/MXene heterostructure induces a strong nanoconfinement effect on the solvated Li^+^, which promotes Li^+^ desolvation while physically restricting the diffusion of larger polysulfides (*28*). Cross-sectional SEM analysis (fig. S9_I_ and S9_II_) reveals that the modification layers of TPBD/MXene and TPBD possess comparable thicknesses of 15.5 μm and 15.7 μm, respectively. Furthermore, contact angle measurements demonstrate the superior electrolyte affinity of the composite. The TPBD/MXene- modified separator exhibits a contact angle of 6.9° (fig. S10_I_), lower than that of the pristine TPBD separator (10.5°) (fig. S10_II_). Consequently, this enhanced electrolyte wettability promotes rapid ion flux and effectively reduces the interfacial impedance of the batteries (*29*). The chemical bonding states of the TPBD/MXene hybrid were investigated by X-ray photoelectron spectroscopy (XPS). The high-resolution Ti 2p spectrum of TPBD/MXene reveals three pairs of peaks centered at binding energies of (462.62 eV, 456.55 eV), (464.3 eV, 458.83 eV), and (461.16 eV, 455.16 eV), which are assigned to Ti-C-O, Ti-O, and Ti-C bonds, respectively (Fig. 1H) (*30*). Notably, the TPBD/MXene heterostructure exhibits distinctive binding energy shifts compared to pristine MXene. This phenomenon underscores the strong anchoring of TPBD onto the MXene surface, creating a robust heterointerface that enhances structural stability. Additionally, the C 1 s spectrum of TPBD/MXene was deconvoluted into four components positioned at 289 eV, 286.3 eV, 285.2 eV, and 282.04 eV, corresponding to C=O, C-O, C-C, and C-Ti bonds, respectively (fig. S11). In contrast to pristine MXene, the intensity of the C-Ti peak in TPBD/MXene is significantly attenuated. This reduction stems from dense coverage by the TPBD layer, which serves as direct evidence of TPBD grafting onto MXene (*31*). Collectively, the robust chemical integration within the TPBD/MXene heterostructure facilitates strong interfacial interactions, leading to charge redistribution and the formation of localized electric fields. These effects weaken solvent-ion interactions, destabilize the solvation shell, and thus accelerate desolvation kinetics. Meanwhile, the porous structure facilitates rapid ion diffusion and effective polysulfide adsorption.

### Synergistic effects of TPBD/MXene on Li^+^ solvation and desolvation kinetics

The ionic speciation in the IHP was systematically investigated. Synergistic regulation of Li(solvent)*_x_*^+^ behavior through electrochemical tandem catalysis and spatial nanoconfinement based on TPBD/MXene is illustrated in Fig. 2A. Specifically, the abundant polar functional groups and uniformly structured nanochannels within the TPBD layer facilitate the pre-organization of solvated ions and guide their directional migration. Across the heterointerface, charge redistribution between the TPBD and MXene generates a strong localized electric field that disrupts the coordination equilibrium between ions and solvent molecules, thereby destabilizing the solvation shell. Simultaneously, the heteroatoms and exposed metal active sites at the interface serve as Lewis acid/base centers, which lower the energy barrier for dissociation. To validate this, in situ Raman spectroscopy was employed to directly probe the reorganization of the solvation shell. The Li^+^ solvation structure in ether-based electrolytes (LE) is resolved into three states: SSIPs, CIPs, and AGGs, whose distribution correlates directly with the Li^+^-solvent interaction strength. As depicted in figs. S12 to S14, the characteristic TFSI^−^ peak appears at 740 cm^−1^ (free anions) with 1 M electrolyte. In contrast, at the TPBD and TPBD/MXene interfaces, this peak shifts to higher wavenumbers, accompanied by emerging spectral signatures of CIPs and AGGs, indicating markedly enhanced Li^+^-anion coordination, which is similar to that in PP with 3 M electrolyte. Quantitative analysis reveals that the CIP + AGG proportion reaches 94% at the TPBD/MXene interface, notably higher than the values for PP (46% for 1 M and 92% for 3 M) and TPBD (89%).

**Fig. 2.**
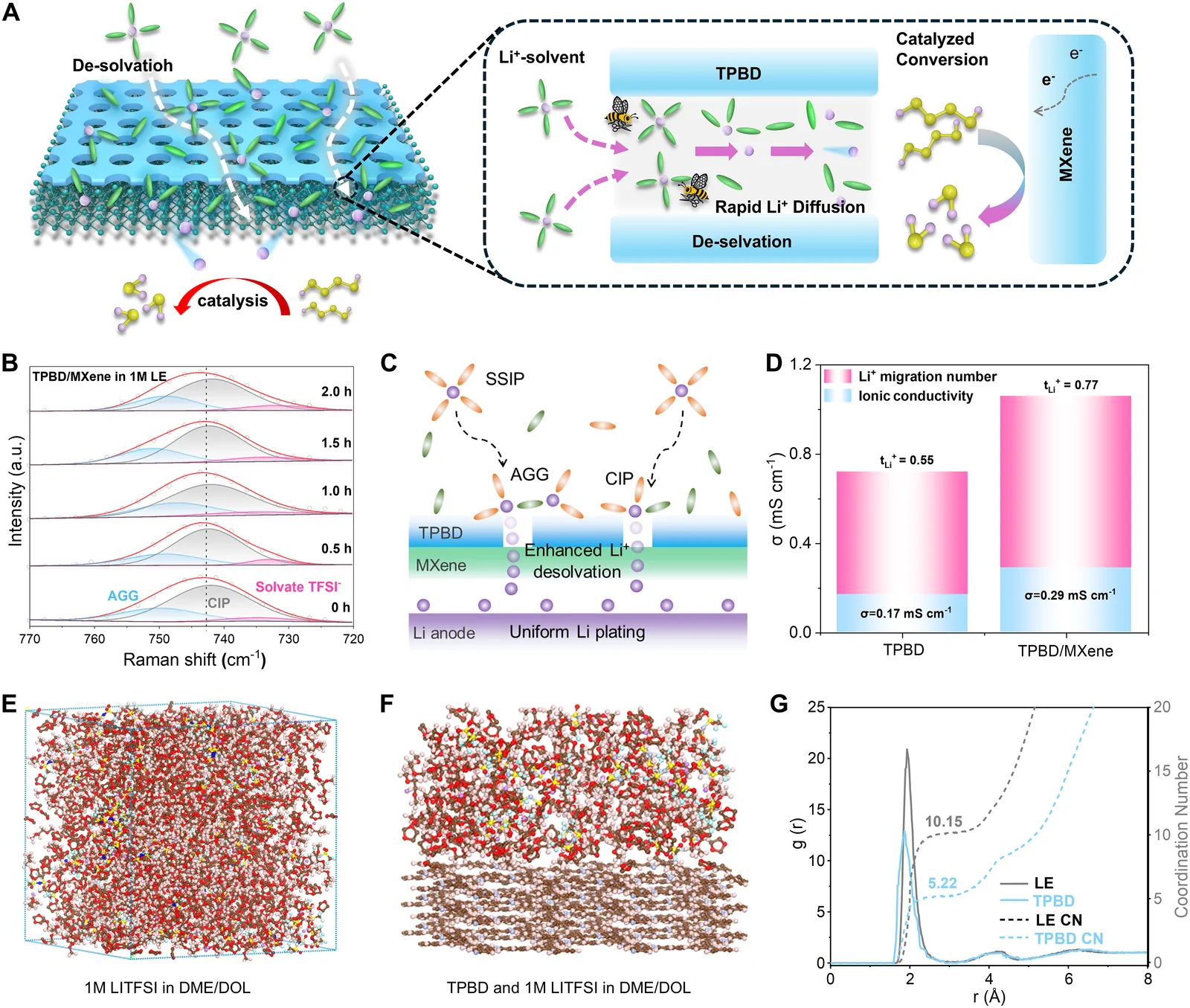
Synergistic regulation of Li^+^ solvation structure and desolvation kinetics. **(A)** Schematic illustration of desolvation catalysis and ionic sieving. **(B)** In situ Raman spectra of electrolyte with TPBD/MXene separator and **(C)** corresponding schematic of Li^+^ solvation structure evolution. **(D)** Li^+^ transference numbers and ionic conductivities. **(E** and **F)** Representative MD simulation snapshots of commercial electrolyte without and with TPBD. **(G)** RDFs and corresponding coordination numbers for Li^+^-O interactions.

During discharge, the solvated TFSI^−^ peak gradually shifts to lower wavenumbers (Fig. 2B), reflecting a dynamic increase in the CIP + AGG proportion from 91.0% to 94.5%. This sustained high concentration of CIPs/AGGs serves as robust quantitative evidence for the in situ reconstruction of the IHP. Consequently, the persistent expulsion of bulky solvent molecules creates a local anion-rich environment that accelerates Li^+^ desolvation kinetics, as schematically illustrated in Fig. 2C. These results demonstrate that the engineered interface effectively restructures the solvation microenvironment, weakening Li^+^ solvent binding and creating a locally concentrated electrolyte region. Therefore, the desolvation barrier is significantly lowered, facilitating rapid ion transport kinetics. Moreover, the TPBD/MXene heterostructure delivers an ionic conductivity of 0.29 mS cm^−1^, approximately 1.7-fold higher than that of the pristine TPBD system (0.17 mS cm^−1^). Concurrently, the Li^+^ transference number is significantly enhanced from 0.55 to 0.77 (Fig. 2D and figs. S15 and S16), suggesting improved Li^+^ transport kinetics. To further elucidate the mechanism underlying these solvation structural changes, molecular dynamics (MD) simulations were employed. As shown in Fig. 2, E to G, the intensity of the Li-O radial distribution function (RDF) peak at 1.93 Å, corresponding to Li^+^ coordination with DME/DOL (LE) (*32*), weakens upon the introduction of TPBD, indicating a modified solvation structure. The coordination number (CN) of Li^+^ with oxygen decreases from 10.15 to 5.22, reflecting a weakened solvation shell that facilitates desolvation. Consistently, the simulated Li^+^-solvent interaction energy decreases in the TPBD system, demonstrating a lower desolvation barrier (*33*). Collectively, these experimental and computational results confirm that the TPBD/MXene heterostructure effectively regulates the Li^+^ solvation environment, weakens Li^+^ solvent binding, and enhances Li^+^ mobility.

### Bifunctional TPBD/MXene for enhanced desolvation and polysulfide immobilization

The side-view (Fig. 3, A and B) and top-view (fig. S17) differential charge density distributions reveal that the TPBD/MXene heterostructure induces pronounced charge transfer (yellow isosurfaces) with Li(DME)_4_^+^, redistributing electron density. The adsorption capabilities of the typical Li(DME)_4_^+^ cluster and TFSI^−^ anions in the solvation shell are compared (Fig. 3C). The results indicate that TPBD/MXene exhibits stronger adsorption energies toward both Li(DME)_4_^+^ and TFSI^−^ compared to pristine TPBD reinforcing interfacial interaction behaviors. Additionally, the dissociation energy barrier for the Li(DME)_4_^+^ was calculated step by step. Generally, the dissociation barrier increases with the coordination number of solvent molecules around Li^+^. The influence of the electronic structure of the TPBD/MXene heterostructure on successive polysulfide conversion was also analyzed. Compared to pristine TPBD, the density of states (DOS) of the TPBD/MXene heterostructure shifts closer to the Fermi level (fig. S18), a compelling indication of facilitated charge transfer at the heterointerface (*34*). Specifically, the dissociation of Li(DME)_4_^+^ requires overcoming a barrier of only 0.2 eV on the TPBD/MXene surface (Fig. 3D). In sharp contrast, on the pristine TPBD surface, this barrier is as high as 0.4 eV. These results demonstrate that TPBD/MXene effectively lowers the dissociation energy barrier, thereby facilitating the release of Li^+^ from the solvation structure.

**Fig. 3.**
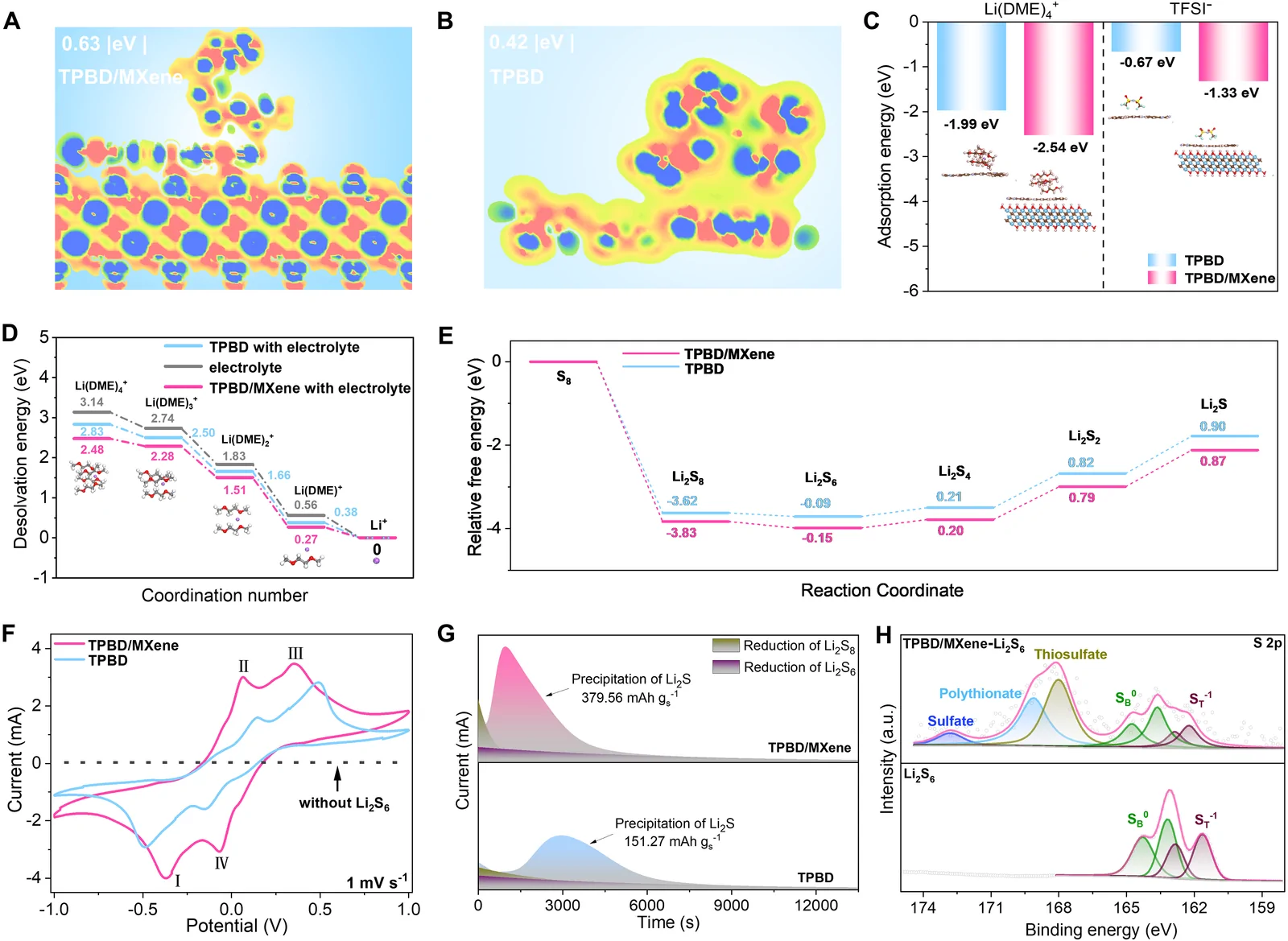
Promoting Li^+^ desolvation and polysulfide anchoring. Side-view differential charge density of Li(DME)_4_^+^ on **(A)** TPBD/MXene and **(B)** TPBD. **(C)** Binding energies of Li(DME)_4_^+^ and TFSI^−^ on diverse substrates. **(D)** Desolvation energetics. **(E)** Gibbs free energy diagrams. **(F)** CV plots of symmetric cells. **(G)** Potentiostatic discharge curves of Li_2_S. **(H)** S 2p XPS spectra before and after polysulfide adsorption.

Furthermore, DFT calculations were performed to investigate the interaction mechanisms between TPBD/MXene and polysulfides. As illustrated in fig. S19, the adsorption energies of S_8_, Li_2_S, Li_2_S_2_, and long-chain polysulfides (Li_2_S_n_, 4 ≤ n ≤ 8) are significantly higher on TPBD/MXene than on TPBD. This indicates that TPBD/MXene can effectively alleviate the dissolution and migration of lithium polysulfides (LiPSs), thus improving the cyclability of Li-S batteries (*35*). To gain a deeper understanding of the interplay between LiPSs and the substrate, the charge density difference for Li_2_S_6_ adsorbed on TPBD (fig. S20) and TPBD/MXene (fig. S21) was analyzed. The blue isosurfaces surrounding the Li-S bonds indicate a reduction in charge density, suggesting a weakening of the interatomic interactions within the LiPSs. The electron accumulation on TPBD/MXene exceeds that on TPBD, indicating that the heterostructure possesses superior anchoring capabilities for polysulfides, which aligns well with the experimental results (*36*). Beyond that, charge accumulation (yellow) and depletion (blue) are concentrated at the TPBD and MXene sides of the interface, respectively (fig. S22). This confirms the presence of a built-in electric field within the TPBD/MXene heterostructure, which significantly optimizes electron transfer and accelerates the multi-step conversion reaction of sulfur. Figure 3E presents the calculated Gibbs free energy (ΔG) profiles for the reduction of sulfur species. The corresponding optimized geometries for adsorbed lithium polysulfides and S_8_ are presented in fig. S23. The transformation from solid Li_2_S_2_ to Li_2_S emerges as the pivotal rate-determining step due to its inherently sluggish kinetics (*37*, *38*). Notably, the TPBD/MXene heterostructure demonstrates a more thermodynamically favorable energy landscape (lower ΔG) compared to TPBD, particularly for the rate-limiting step. This result elucidates that the heterostructure effectively promotes the redox kinetics of LiPSs conversion. To evaluate self-discharge behavior, Li-S cells were rested for one week after 10 cycles at 0.2 C (fig. S24A) (*39*, *40*). The TPBD/MXene-based batteries sustained a higher final voltage of 2.20 V during the resting period compared to the PP separator. Furthermore, it exhibited a minimal capacity loss of only 4.39% upon resuming cycling (fig. S24B). These results underscore the exceptional capability of TPBD/MXene in inhibiting self-discharge.

To investigate the catalytic activity, cyclic voltammetry (CV) tests of symmetric cells with Li_2_S_6_ electrolyte were conducted within a potential window of −1.0 to 1.0 V at 1 mV s^−1^. As shown in Fig. 3F, the cathodic peaks “I” and “IV” are assigned to the reduction of S_8_ to Li_2_S_6_ and subsequently to Li_2_S/Li_2_S_2_. In contrast, the anodic peaks “II” and “III” represent the reversible oxidation of Li_2_S_2_/Li_2_S to Li_2_S_6_, and ultimately to S_8_. Apparently, the batteries with the TPBD/MXene-modified separator exhibit a higher response current, a narrower potential gap, and a larger integrated area compared to pristine TPBD. This demonstrates that TPBD/MXene effectively facilitates Li^+^ desolvation and catalyzes redox reactions, thereby enhancing ion transport kinetics (*41*). Given that the conversion of soluble polysulfides to solid Li_2_S accounts for approximately three-quarters of the total discharge capacity, Li_2_S nucleation experiments were performed to evaluate this critical step (*42*, *43*). During the Li_2_S nucleation experiment, the time-current profile under constant-voltage discharge at 2.06 V reveals distinct stages. The yellow region represents the reduction of Li_2_S_8_, where Li_2_S_8_ combines with Li^+^ to form soluble Li_2_S_6_ (purple region). Subsequently, Li_2_S_6_ undergoes lithiation to convert into Li_2_S_4_. Further lithiation promotes the electrochemical reduction of Li_2_S_4_, and a prominent Li_2_S deposition peak emerges during constant-voltage discharge at 2.02 V (*44*–*46*). The TPBD/MXene electrode delivered an impressive nucleation capacity of 379.56 mAh g^−1^, significantly exceeding that of TPBD (151.27 mAh g^−1^) (Fig. 3G). This substantial increase indicates a reduced nucleation energy barrier on the TPBD/MXene surface, which expedites the bidirectional conversion kinetics and boosts overall rate performance (*47*, *48*). Moreover, the internal resistance and dynamic polarization during charge/discharge were evaluated using the Galvanostatic Intermittent Titration Technique (GITT) at 0.1 C (*49*). As depicted in figs. S25 and S26, the TPBD/MXene-based batteries exhibit reduced polarization, evidenced by the smaller gap between the closed-circuit voltage (CCV) and quasi-open-circuit voltage (QOCV) during Li_2_S nucleation and activation (*50*). Beyond that, optical absorption tests (fig. S27) confirm that the strong anchoring capability of TPBD/MXene effectively mitigates the shuttle effect of polysulfides (*51*).

To elucidate the chemisorption mechanism, XPS was conducted after Li_2_S_6_ adsorption. In the high-resolution S 2p spectrum (Fig. 3H), two characteristic doublets located at 164.7/163.6 eV and 162.8/162.2 eV are assigned to bridging sulfur (SB^0^) and terminal sulfur (ST^−1^) bonds, respectively. Evidently, these peaks exhibit a distinct positive binding energy shift compared to those of pure Li_2_S_6_, indicating strong chemical interaction between the polysulfides and the host material. In addition, three new peaks detected at 172.85, 169.09, and 168.02 eV are attributed to sulfate, polysulfate, and thiosulfate species, respectively (*52*). As revealed in fig. S28A, the Ti 2p binding energies for Ti-O, Ti-C-O, and Ti-C bonds in TPBD/MXene-Li_2_S_6_ exhibit a positive shift compared to those before adsorption, indicating electron transfer from Ti to polysulfides (*53*). Besides, the Li 1 s spectrum (fig. S28B) displays two distinct peaks at 56.6 and 55.4 eV, corresponding to Li-N and Li-S bonds, respectively. The formation of Li-N bonds specifically validates the strong lithiophilic interaction provided by the heterostructure (*54*). Collectively, these results substantiate the superior capability of TPBD/MXene to immobilize LiPSs, thereby accelerating bidirectional sulfur redox kinetics.

### COMSOL simulations of dendrite suppression via modified separators

To elucidate how the TPBD/MXene-modified separator regulates the electric field, Li^+^ concentration, and current distributions, finite element simulations were conducted using COMSOL Multiphysics (*55*, *56*). As illustrated in Fig. 4, A, C, and E and fig. S29, A, C, and E, the TPBD/MXene-modified separator establishes a uniform electric field, a homogeneous Li^+^ concentration profile, and an evenly distributed current density over time. This regulation is attributed to the dynamic electrostatic shielding effect of the functional layer, which effectively diverts the high-flux Li^+^ from high-field protrusions toward low-energy planar sites, thereby eliminating localized deposition hotspots and enabling uniform lithium plating. In contrast, the bare PP separator system exhibits severe electric field distortion, localized Li^+^ concentration polarization, and pronounced current density enhancement at the tips of lithium protrusions as the deposition proceeds (Fig. 4, B, D, and F and fig. S29, B, D, and F). Such inhomogeneities inevitably drive preferential lithium nucleation at high-activity asperities, accelerating the growth of dendritic structures. Li//Li symmetric cells (1 mAh cm^−2^ stripping/plating) employing the modified separator exhibited a reduced nucleation overpotential (≈16.9 mV after 1000 h) and consistently lower polarization across current densities of 0.5–5 mA cm^−2^, compared to cells with a conventional PP separator (fig. S30, A and B). To probe its function in a practical system, we analyzed the lithium anodes from Li-S batteries after 100 cycles at 0.2 C. With the PP separator (fig. S31, A to C), severe lithium dendrite formation and sulfur deposition were observed on the anode, a consequence of polysulfide shuttling and subsequent reduction (*57*, *58*). Conversely, the TPBD/MXene-modified separator enabled a dendrite-free anode with negligible sulfur deposition (figs. S31, D to F and S32), directly demonstrating the coating’s dual role in homogenizing lithium deposition and alleviating polysulfide shuttling. More critically, this composite induces homogeneous Li plating and forms a dense, durable protective film to restrain dendrite proliferation. As a result, TPBD/MXene outperforms bare TPBD in rapid polysulfide trapping and accelerated LiPS redox kinetics, thus enabling long-term stable cycling of Li-S cells. These findings correlate with the enhanced cycling stability, confirming the functional separator as a critical enabler for durable Li-S batteries.

**Fig. 4.**
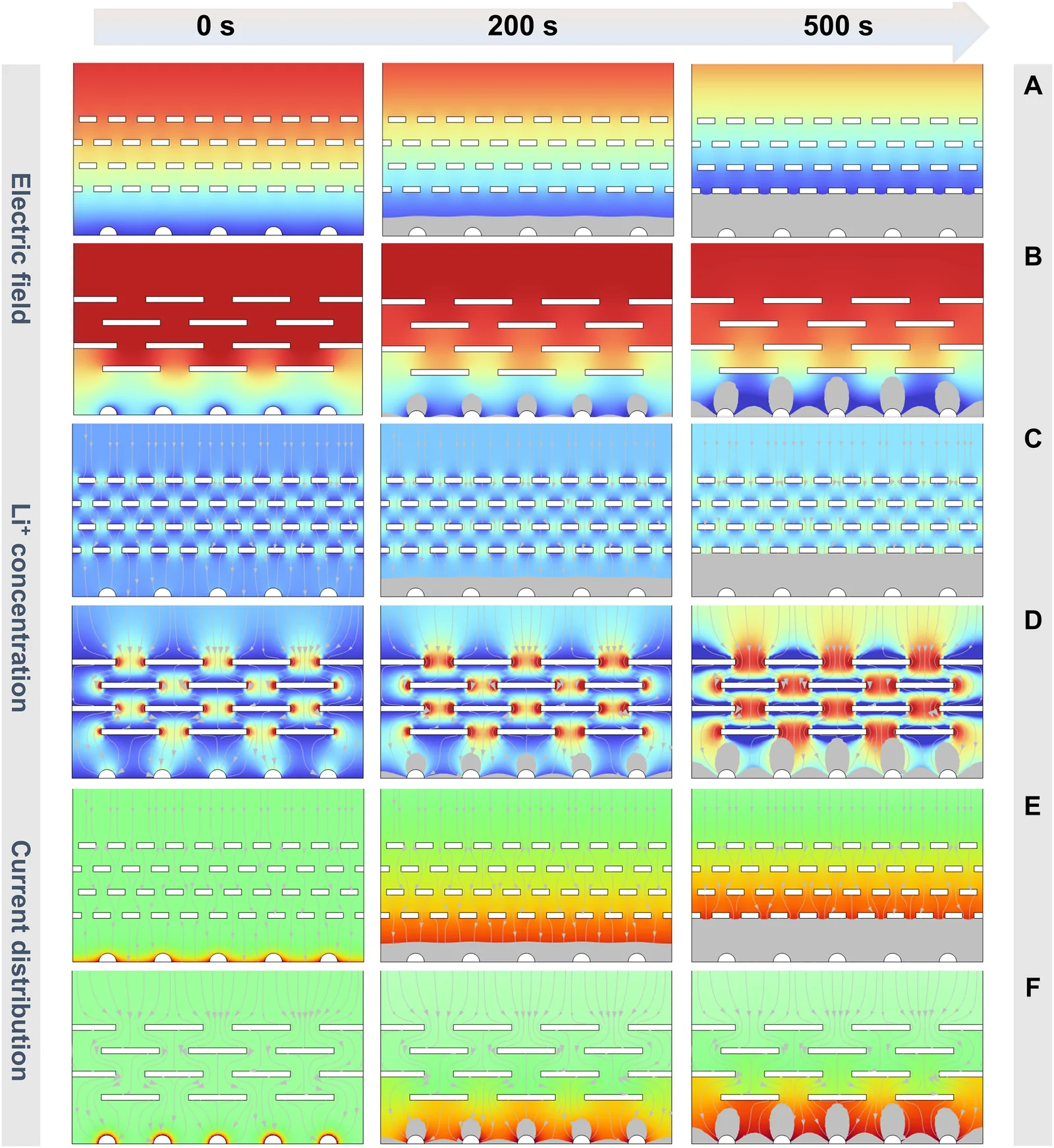
COMSOL simulation of modified separator for suppressing lithium dendrites. Electric field distribution, Li^+^ concentration, and current distribution with **(A, C,** and **E)** TPBD/MXene separator and **(B, D,** and **F)** PP separator.

### In situ and ex situ evidence for polysulfide shuttling suppression and interfacial stabilization

To verify the process described above, operando spatial mapping of sulfur species was performed via time-of-flight secondary ion mass spectrometry (TOF-SIMS) with complementary 2D and 3D analyses (*59*, *60*). The depth sputtering profiles reveal that the TPBD/MXene-modified separator exhibits a persistently lower LiS_2_^−^ signal compared to the TPBD-modified separator (Fig. 5A), underscoring the efficacy of TPBD/MXene in alleviating the shuttle effect. This suppression originates from accelerated conversion kinetics enabled by the ion-conductive framework of TPBD/MXene. This finding is further corroborated by the fewer LiS_2_^−^ regions observed in the TOF-SIMS surface profile of the TPBD/MXene system (Fig. 5, B and C), along with the greater distribution of Li_2_S_2_ shown in the corresponding 3D reconstruction images of the electrode (fig. S33, A and B), demonstrating the prominent catalytic activity of TPBD/MXene toward LiPSs conversion. In addition, the higher F^−^ content on the TPBD/MXene separator surface compared to TPBD (Fig. 5D), as revealed by both 2D (Fig. 5, E and F) and 3D TOF-SIMS mappings (fig. S33, C and D), indicates the formation of a LiF-rich protective layer derived from controlled decomposition of LiTFSI. This layer effectively stabilizes the interface and alleviates further electrolyte decomposition, thereby demonstrating superior interfacial stability in Li-S batteries.

**Fig. 5.**
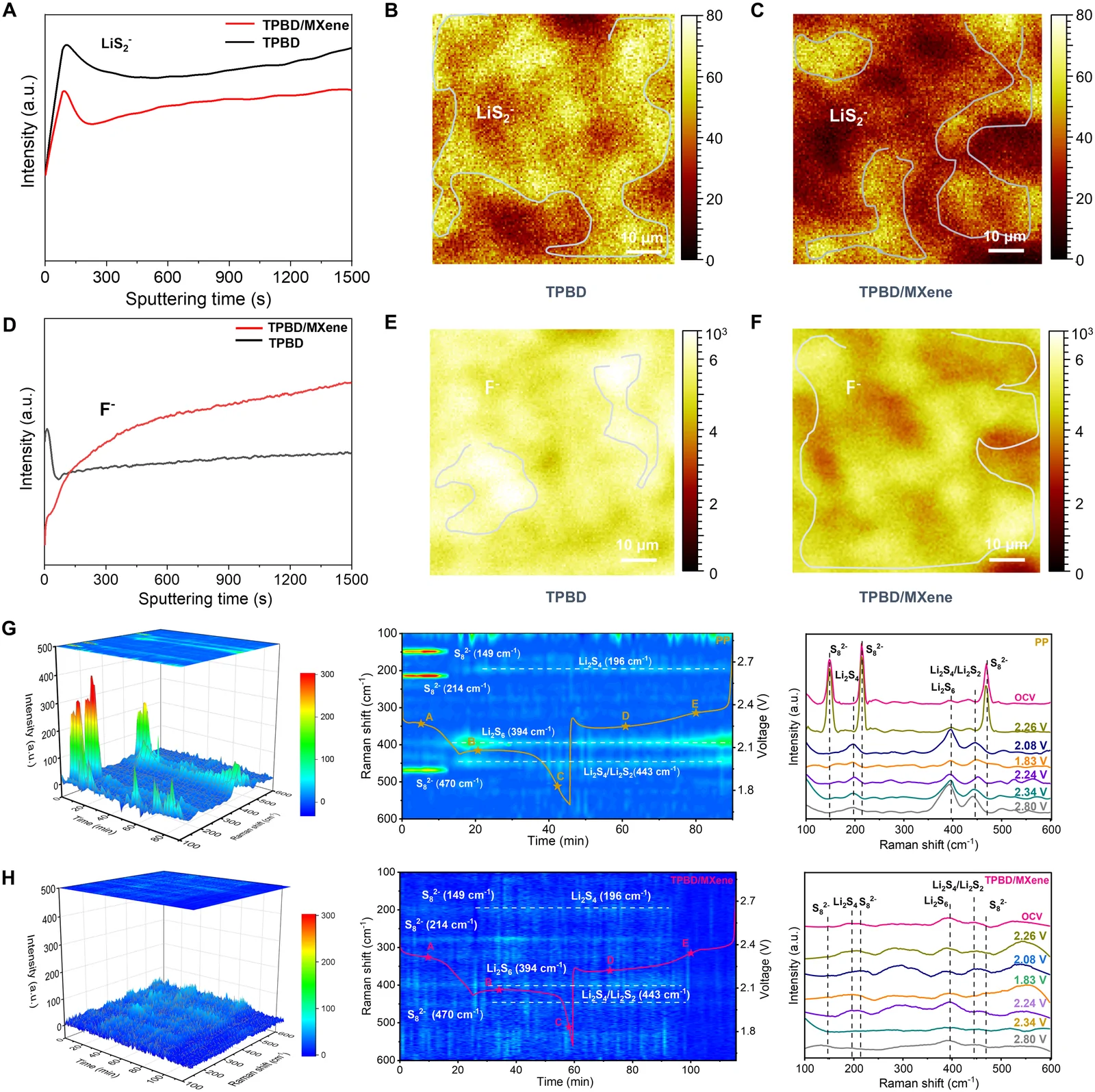
Polysulfide shuttling suppression and interface stabilization mechanism. Depth-profiling analysis of **(A)** LiS_2_^−^ and **(D)** F^−^ species. 2D secondary ion distribution images of **(B** and **C)** LiS_2_^−^ and **(E** and **F)** F^−^ on TPBD/MXene and TPBD-modified separators. Time-resolved in situ Raman spectra of Li-S batteries using **(G)** PP and **(H)** TPBD/MXene separators during cycling.

To probe the dynamic evolution of LiPSs during electrochemical cycling, in situ Raman spectroscopy was conducted. Figure 5, G and H present the Raman spectra acquired during the galvanostatic charge-discharge cycles (0.5 C) of Li-S batteries equipped with PP (Fig. 5G) and TPBD/MXene (Fig. 5H) modified separators, respectively. In the PP separator system, characteristic peaks of S_8_^2−^ and S_6_^2−^ were observed sequentially in the early discharge stage, followed by a progressive transformation into S_6_^2−^, S_4_^2−^, and S_2_^2−^ as discharge proceeded. During the subsequent charge process, signals corresponding to S_4_^2−^ and S_2_^2−^ persisted, albeit with diminished intensity, consistent with ongoing polysulfide shuttling. In striking contrast, the TPBD/MXene separator exhibits virtually no detectable polysulfides-related signals over the entire charge-discharge cycling. Spectra collected at various states of charge and discharge further confirmed the absence of characteristic polysulfides peaks. These observations indicate that the TPBD/MXene coating effectively inhibits the migration of soluble polysulfides toward the anode, thus preventing deleterious side reactions between polysulfides and metallic lithium.

### Comprehensive electrochemical evaluation of Li-S batteries using TPBD/MXene separators

The CV contour maps at various scan rates (Fig. 6A) show that TPBD/MXene delivers higher current densities at the two cathodic peaks and the anodic peak, outperforming batteries with TPBD (fig. S34A) and PP (fig. S34B) separators. Figure S35 presents the CV profiles of Li-S cells using TPBD/MXene, TPBD, and bare PP separators. The positions and potential intervals of the oxidation (Peak C) and reduction peaks (Peaks A and B) serve as critical indicators of reaction kinetics (*34*, *61*). Notably, the TPBD/MXene-based separator yields the sharpest redox peaks with the smallest voltage hysteresis, indicating reduced polarization due to enhanced Li^+^ transport and redox kinetics. Ion transport kinetics were further probed via multi-scan CV from 0.1 to 0.5 mV s^−1^ (fig. S36, A to C). According to the Randles-Sevcik formula, the TPBD/MXene system exhibits the steepest slopes and consistently the highest Li^+^ diffusion coefficients (D_Li_^+^) for all redox peaks compared to control samples (figs. S37 and S38 and table S1), confirming that the heterostructure effectively accelerates Li^+^ mass transfer and redox kinetics. To evaluate the catalyst-enhanced kinetics in Li-S batteries, full cells were assembled using various functional separators. The distribution of relaxation times (DRT) method was subsequently employed to resolve the impedance components. The TPBD/MXene-modified separator (figs. S39A and S40A) significantly reduces both resistance components compared to the TPBD counterpart (figs. S39B and S40B), confirming accelerated transport kinetics. At 0.2 C (1 C = 1675 mAh g^−1^), galvanostatic charge-discharge profiles (fig. S41) reveal that TPBD/MXene manifests the most extended high-potential (Q_H_) and low-potential (Q_L_) plateaus, indicating maximized sulfur utilization. The TPBD/MXene-based cell maintains 1183.6 mAh g^−1^ after 100 cycles, significantly outperforming the TPBD (912.3 mAh g^−1^) and PP (514.6 mAh g^−1^) systems (fig. S42). Furthermore, the heterostructure system demonstrates the lowest voltage hysteresis (ΔE, fig. S43), further confirming its accelerated redox kinetics and reduced polarization (*62*).

**Fig. 6.**
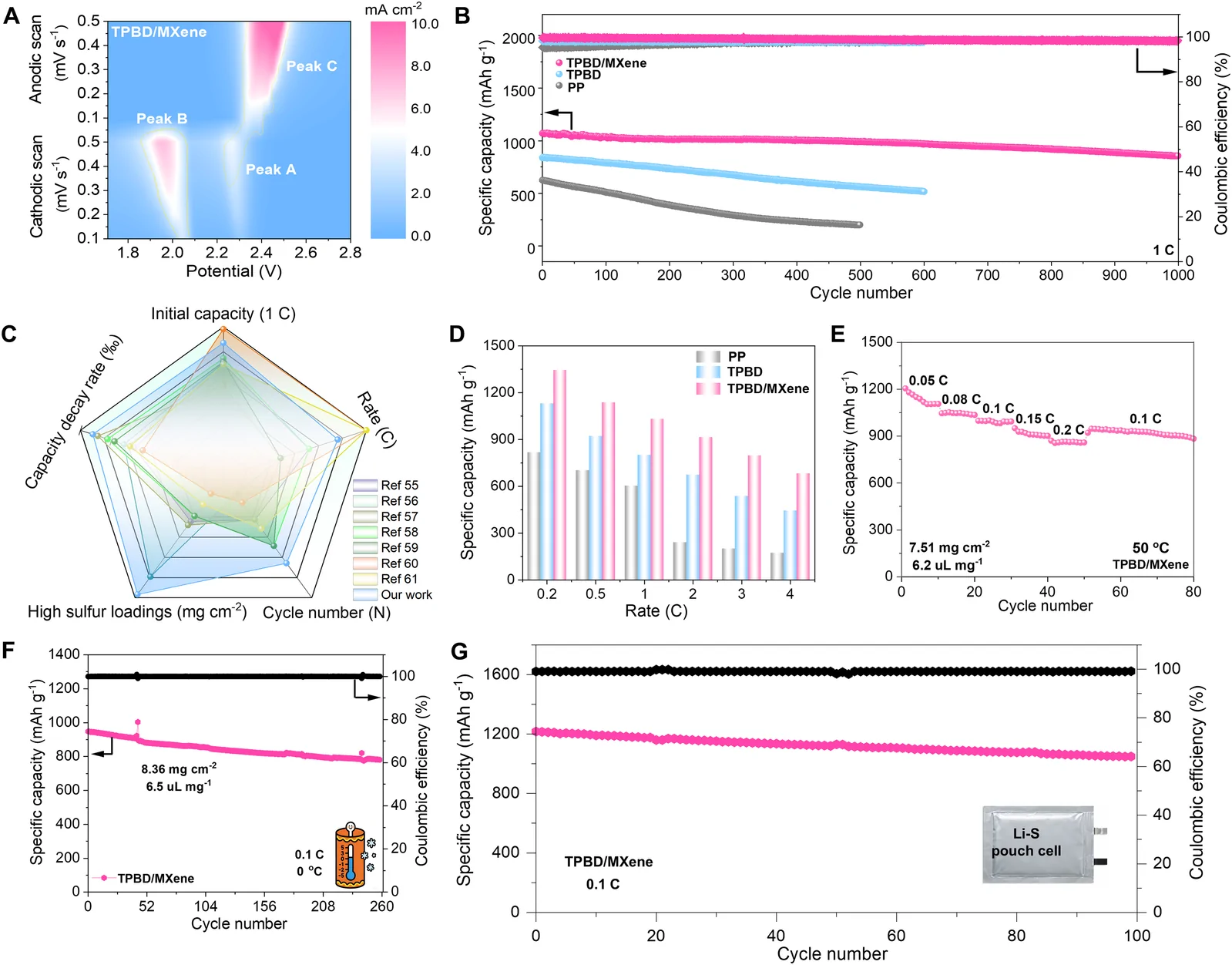
Electrochemical behavior of Li-S batteries. **(A)** CV contour plots at different scan rates. **(B)** Cycling performance at 1 C. **(C)** Performance comparison with reported works. **(D)** Rate performance at room temperature. **(E)** Rate performance at 50°C. **(F)** Cycling performance at 0°C. **(G)** Cyclability of pouch cells.

Long-term cyclic stability was further evaluated at 1 C over 1000 cycles, as demonstrated in Fig. 6B. The cell with heterostructure delivers an initial capacity of 1070.9 mAh g^−1^ and retains 856.6 mAh g^−1^ after 1000 cycles, signifying an ultralow capacity decay rate of 0.02% per cycle. This electrochemical performance compares favorably with other state-of-the-art results reported in the literature (Fig. 6C and table S2). The batteries utilizing the heterostructure separator demonstrate superior rate capability, delivering specific capacities of 1343.9, 1137.3, 1031.7, 914.8, 796.7, and 682.1 mAh g^−1^ at current densities from 0.2 to 4 C, respectively. Upon returning to 0.5 C, the capacity recovers to 1034 mAh g^−1^ (Fig. 6D and fig. S44), outperforming the control cells, particularly at higher rates. As shown in fig. S45, the TPBD/MXene system retains a distinct liquid-solid conversion plateau even at high rates. This exceptional rate performance is ascribed to enhanced Li^+^ desolvation and diffusion kinetics. As presented in fig. S46, the heterostructure-based batteries achieve a remarkable specific capacity of 1032 mAh g^−1^ under a high sulfur loading of 9.72 mg cm^−2^, corresponding to a high areal capacity of 10.03 mAh cm^−2^. After 70 cycles, it maintains 8.16 mAh cm^−2^. To assess practical feasibility, we evaluated cells with high-mass-loading cathodes under wide-temperature conditions. The batteries with heterostructure-separator maintain good rate performance at 50°C under an areal sulfur loading of 7.51 mg cm^−2^, delivering a high initial specific capacity of 1205.4 mAh g^−1^ at 0.05 C (Fig. 6E). Even at 0°C (Fig. 6F), the batteries equipped with the TPBD/MXene-modified separator display a notable areal capacity of 6.53 mAh cm^−2^ after 259 cycles, under a high loading of 8.36 mg cm^−2^ and a lean electrolyte/sulfur (E/S) ratio of 6.5 μL mg^−1^. Furthermore, the pouch cell with heterostructure delivers an appreciable specific capacity of 1216.6 mAh g^−1^ and maintains 1049.1 mAh g^−1^ after 100 cycles (Fig. 6G). Post-failure analyses (figs. S47 and S48) reveal a roughened Li anode with distinct S/F signals on the separator, collectively confirming that cell failure is driven by the shuttle effect and accumulation of Li_2_S and byproducts. These results confirm that the proposed confinement tandem catalysis strategy effectively regulates the solvation structure within the IHP, thereby ensuring robust electrochemical performance.

## DISCUSSION

To modulate the IHP, a synergistic strategy combining electrochemical tandem catalysis with spatial nanoconfinement is proposed to regulate the Li(solvent)*_x_*^+^ dynamics. Unlike conventional electrolyte engineering, this interfacial design provides a dual-functional platform: the built-in electric field and ordered porous channels drastically lower the activation energy for the conversion of polysulfides, while simultaneously homogenizing the Li^+^ flux to inhibit dendrite propagation. Consequently, under a sulfur loading of 9.72 mg cm^−2^, the cell achieves an exceptional areal capacity of 10.03 mAh cm^−2^. Even at 0°C, the battery retains a high areal capacity of 6.53 mAh cm^−2^ after 259 cycles under a sulfur loading of 8.36 mg cm^−2^ and a low E/S ratio. Meanwhile, it demonstrates excellent rate capability at 50°C with a high sulfur loading of 7.51 mg cm^−2^. Impressively, the assembled pouch cell also achieves a capacity retention of over 85% after 100 cycles. This catalyst-driven Helmholtz interfacial engineering strategy opens a new avenue for the rational design of high-performance, wide-temperature-range Li-S batteries.

## MATERIALS AND METHODS

### Chemicals and materials

Lithium sulfide (≥99.98%), acetic acid (AR, ≥99.8%), tetraglyme (99%), and sulfur (AR, ≥99.5%) were procured from Aladdin Reagent Co., Ltd. Hydrochloric acid (HCl, A.R. grade) and lithium fluoride (LiF, A.R. grade) were sourced from Shanghai Technology Co., Ltd. Titanium carbide (Ti_3_AlC_2_) was acquired from 11 Technology Co., Ltd. Bis (trifluoromethane) sulfonimide lithium salt (C_2_F_6_LiNO_4_S_2_, ≥99%), benzene-1,3,5-tricarboxaldehyde (BD, 97%), acetonitrile (AR, >99.5%), 4,4′,4″-(1,3,5-triazine-2,4,6-triyl) trianiline (TP, ≥95%), and N-methyl-2-pyrrolidone (C_5_H_9_NO, >99.0%) were purchased from Aladdin Reagent Co., Ltd. Polyvinylidene fluoride (PVDF), 1,3-dioxolane (DOL, 99.9%), and 1,2-dimethoxyethane (DME, 99.5%) were acquired from Suzhou Dodochem Technology Co., Ltd. LA133 binder was purchased from Canrd Technology Co., Ltd. All procured chemicals were utilized without any additional purification procedures.

### Preparation of MXene suspension

MXene suspension was prepared through selectively etching Ti_3_AlC_2_ precursor using a LiF/HCl mixture as the etchant. Specifically, LiF was thoroughly mixed with HCl (9 M) in a Teflon reactor. Subsequently, Ti_3_AlC_2_ (2 g) was slowly introduced into the resulting solution, and the mixture was subjected to magnetic stirring at 35°C. Afterwards, the multilayered Ti_3_C_2_T_x_ was rinsed with deionized water until the supernatant stabilized at pH ≈7. Next, deionized water was added to the Ti_3_C_2_T_x_ slurry, followed by ultrasonication of the mixture under an Ar atmosphere. Finally, the Ti_3_C_2_T_x_ suspension was centrifuged at 3500 rpm to obtain the final product.

### Preparation of TPBD/MXene and TPBD

In a Pyrex tube, 4,4′,4″-(1,3,5-triazine-2,4,6-triyl) trianiline (marked as TP, 0.16 mmol) was blended with acetonitrile and a specified amount of Ti_3_C_2_T_x_, followed by sonication. Subsequently, a mixture of benzene-1,3,5-tricarboxaldehyde (labeled as BD), and acetonitrile (8 mL) was added to the above solution and sonicated for an additional 30 minutes. Afterwards, 12 M CH_3_COOH was added to the Pyrex tube, which was then sealed and maintained at 25°C for 3 days without agitation. The obtained yellow solid was washed and centrifuged using N, N-dimethylformamide, and then placed in a vacuum oven at 50°C for 12 h. TPBD was prepared using an equivalent procedure, but without the addition of MXene.

### Preparation of the S electrode

The carbon nanotube/sulfur (CNT/S) composite was synthesized through a melt diffusion technique. CNT and S were uniformly mixed in a mortar at a mass ratio of 1:4, and the resulting powder was sealed in a ceramic boat and heated at 155°C for 12 h to obtain the CNT/S mixture. The working electrode was prepared by evenly mixing CNT/S, carbon black, and LA133 in a solution of n-propanol with the mass ratio of 8:1:1. This slurry was then coated onto aluminum foil and dried at 50°C for 12 h. The areal sulfur loading was approximately 2 ∼ 10 mg cm^−2^. The fabrication process for high-sulfur-loading cathodes was identical to that of low-sulfur-loading cathodes.

### Preparation of TPBD/MXene and TPBD-modified separators

The as-synthesized materials (TPBD/MXene and TPBD), carbon black, and polyvinylidene fluoride (PVDF) were evenly mixed in N-methy l-2-pyrrolidone (NMP) at a mass ratio of 8:1:1 to form a slurry. Subsequently, the slurry was uniformly coated onto the surface of a commercial PP separator. The coating material was coated onto the cathode-facing side of the separator and dried at 50°C for 12 h. The areal loading of TPBD/MXene and TPBD modified layers on the PP separator was approximately 0.28 mg cm^−2^.

### Electrochemical characterization

The electrochemical performance was studied through the assembly of CR2025 coin cells comprising CNT/S cathode, lithium anode, and separators (TPBD/MXene, TPBD, and PP). The electrolyte comprises a blended solution of 1,3-dioxolane (DOL) and 1,2-dimethoxyethane (DME) in a volume ratio of 1:1, containing 1 M lithium bis (trifluoromethanesulfonyl) imide (LiTFSI), with 2% LiNO_3_ as an additive. The cycling capabilities of different substances were examined within the voltage range of 1.7 to 2.8 V (vs. Li/Li^+^) on a Land battery testing system (Wuhan LAND Electronic Co., Ltd.). Electrochemical Impedance Spectroscopy (EIS) was executed on the CHI760E electrochemical workstation (Chenhua Instrument, Shanghai) within a scan frequency range from 100 kHz to 0.01 Hz with an amplitude of 5 mV. Cyclic voltammetry (CV) experiments were also performed on this workstation. The Li-S pouch cell was constructed by interposing the TPBD/MXene-modified separator between the sulfur cathode and the lithium anode. The size of the positive and negative electrodes in Li-S pouch batteries was 40 mm × 60 mm. The sulfur loading was about 5 mg cm^−2^ and the E/S ratio was retained at 5 μL mg^−1^. The thickness of the lithium strip is 100 μm. The GCD cycling profiles were measured on a Land battery testing system at 0.1 C within the potential window spanning from 1.7–2.8 V. Low-temperature coin cells were assembled with an areal sulfur loading of 8.36 mg cm^−2^ and an E/S ratio of 6.5 μL mg^−1^. Before testing, the assembled cells were rested at room temperature for 12 h to ensure complete electrolyte infiltration, then transferred to a temperature-controlled chamber preset at 0°C for electrochemical cycling. All GCD measurements were conducted on a Neware battery test system at 0.1 C within a potential window of 1.7–2.8 V. The key electrochemical metrics under various operational conditions are summarized in table S3. All batteries were assembled in an argon-filled glovebox. The Li^+^ diffusion coefficient (D_Li_^+^) of the batteries was derived from CV curves according to the Randles-Sevcik equation

$$I_{p}=\left(2.69\times 10^{5}\right)n^{1.5}AD_{\text{Li}}^{+0.5}v^{0.5}C_{\text{Li}}$$

where *I_p_* symbolizes the peak current, *n* represents the number of electrons transferred in the reaction (n = 2), *A* stands for the area of electrode material, *D*_Li_^+^ denotes the lithium-ion diffusion coefficient, *C*_Li_ is the Li^+^ concentration, 𝑣 implies the CV scanning rates (V s^−1^). There is a discernible linear correlation between Ip and 𝑣^1/2^.

### Li_2_S_6_ visualized adsorption tests

Li_2_S_6_ solution was made by mixing Li_2_S and S powders (molar ratio 1:5) in DME/DOL (1:1 by volume), followed by continuous stirring until the substances were fully dissolved. The same amounts of TPBD/MXene and TPBD were separately dispensed into a 2 mL of the Li_2_S_6_ solution. Subsequently, the adsorption ability of the as-synthesized materials towards the Li_2_S_6_ solution was verified by ultraviolet-visible (UV-vis) spectroscopic analysis.

### Assembly and measurement of Li_2_S_6_ symmetric cell

The symmetric cells were assembled using identical working and counter electrodes, which were constructed from the active material (either TPBD/MXene or TPBD), carbon black, and LA133 at a weight ratio of 8:1:1. The prepared Li_2_S_6_ solution was utilized as the electrolyte, which was then dispensed onto the counter electrode and the working electrode. CV measurements were performed from −0.1 V to 0.1 V, employing a scanning rate of 1 mV s^−1^.

### Li_2_S nucleation tests

Li_2_S_8_ solution was synthesized by dissolving Li_2_S and S at a molar ratio of 1:7 in tetraglyme, stirring at 60°C for 24 h. The as-fabricated TPBD/MXene and TPBD materials were used as cathodes, while lithium foil served as the anode, and commercial PP was utilized as the separator. The electrolyte containing 0.25 M Li_2_S_8_ with 1.0 M LiTFSI in tetraglyme was dropped onto the cathode, and 1 M LiTFSI in tetraglyme was added into the anode side. The assembled batteries were galvanostatically discharged to 2.06 V at 0.112 mA and held at a constant voltage of 2.05 V to promote Li_2_S growth. The battery was assembled within a glovebox that was fully purged with argon.

### Materials characterization

Details of the materials characterization can be found in the Supplementary Materials.

### Theoretical calculations

Details of the computational methods can be found in the Supplementary Materials.

## Supplementary Materials

### This PDF file includes:

Supplementary Text Figs. S1 to S48 Tables S1 to S3 References

## References

[1] S. Kim, W.-G. Lim, H. Jung, Y. C. Jeong, C.-Y. Park, S. B. Yang, C. H. Lee, D. Wang, K. Sohn, J. W. Han, J. Lee, Protective catalytic layer powering activity and stability of electrocatalyst for high-energy lithium-sulfur pouch cell. Nat. Commun. 16, 1649 (2025). 10.1038/s41467-025-56606-2 PMC11828906 39952971

[2] H. Chen, Y. Qiu, Z. Cai, W. Liang, L. Liu, M. Li, X. Hou, F. Chen, X. Zhou, T. Cheng, L. He, J. Wang, X. Zhang, S. Dou, L. Li, Topological Insulator Heterojunction with Electric Dipole Domain to Boost Polysulfide Conversion in Lithium-Sulfur Batteries. Angew. Chem. Int. Ed. Engl. 64, e202423357 (2025). 10.1002/anie.202423357 39743867

[3] Y. He, D. Xiong, M. Chen, W. Zhang, S. Liu, Y. Ye, M. Wang, Y. Chen, Q. Tang, X. Peng, C. Wang, H. Zhan, H. Liu, M. Liu, J. Su, H. Shu, J. Wang, X. Wang, Modulating Ion-Dipole and Dipole–Dipole Interactions for Stable Wide-Temperature-Range Lithium–Sulfur Batteries Enabled by Quantum-Dot Catalysts. Angew. Chem. Int. Ed. Engl. 64, e202512168 (2025). 10.1002/anie.202512168 PMC12455398 40761161

[4] T. Li, Y. Liu, J. Wang, H. Hao, Z. Yu, H. Liu, Constructing metal telluride-grafted MXene as electron “donor-acceptor” heterostructure for accelerating redox kinetics of high-efficiency Li-S batteries. J. Mater. Chem. A 12, 12691–12701 (2024).

[5] M. H. Parekh, H. Rao, D. Jokhakar, V. P. Parikh, M. Palanisamy, V. G. Pol, Polysulfide shuttle mitigation through a tailored separator for critical temperature energy-dense lithium–sulfur batteries. Sustain. Energy Fuels 6, 5591–5599 (2022).

[6] D. A. Gribble, P. F. Cheng, V. G. Pol, P. R. Kidambi, Size-selective nanoporous atomically thin graphene separators for lithium–sulfur batteries. ACS Appl. Mater. Interfaces 17, 55554–55563 (2025). 10.1021/acsami.5c11148 PMC12492388 40906896

[7] K. Kim, P. J. Kim, J. P. Youngblood, V. G. Pol, Surface functionalization of carbon architecture with nano-MnO_2_ for effective polysulfide confinement in lithium-sulfur batteries. ChemSusChem 11, 2375–2381 (2018). 10.1002/cssc.201800894 29845736

[8] W. Wang, X. Wang, H. Yang, Z. Yu, W. Zhang, H. Huang, Z. Zhao, X. Zheng, L. Liu, Y. Deng, Y. Li, Design rules for anion-doped catalysts revealed by p-p-s orbital coupling in Li-S chemistry. Nat. Commun. 16, 10895 (2025). 10.1038/s41467-025-65908-4 PMC12678606 41345389

[9] Y. Huang, J. Li, Y. Zhang, L. Lin, Z. Sun, G. Gao, B. Sa, L. Wang, L. Ma, S. Lee, M.-S. Wang, D.-L. Peng, K. Amine, Q. Xie, Energizing Robust Sulfur/Lithium Electrochemistry via Nanoscale-Asymmetric-Size Synergism. J. Am. Chem. Soc. 147, 4752–4765 (2025). 10.1021/jacs.4c10238 39875196

[10] T. Li, Y. Liu, J. Wang, H. Hao, Z. Yu, H. Liu, Electronic redistribution and fast delivery enabled by Co_0.5_Ni_0.5_Te_2_ grafting 3D MXene heterostructure for accelerating High-Durability sulfur redox kinetics. Chem. Eng. J. 487, 150502 (2024).

[11] F. Li, H. Yuan, Y. Wang, Z. Xue, M. He, J. Wang, F. Wu, M. Huang, Y. Xiang, A. Hu, W. Chen, T. Wu, X. Sun, Tailoring Li-Accelerated Motif Enables Lithium Stabilization and Polysulfide Conversion for Long-Cycling Li–S Batteries. Adv. Funct. Mater. 35, e11078 (2025).

[12] T. Lai, K. Liao, A. Manthiram, Modulating Li^+^ and Polysulfide Solvation with Low-Density Moderately Solvating Electrolytes for Lithium–Sulfur Batteries. Angew. Chem. Int. Ed. Engl. 65, e22940 (2026). 10.1002/anie.202522940 41482684

[13] J. Wang, J. Zhang, Y. Zhang, H. Li, P. Chen, C. You, M. Liu, H. Lin, S. Passerini, Atom-Level Tandem Catalysis in Lithium Metal Batteries. Adv. Mater. 36, e2402792 (2024). 10.1002/adma.202402792 38616764

[14] J. Zhang, F. Liu, R. He, Q. Guan, N. Tian, J. Wu, Z. Cao, S. Yin, Y. Zhang, L. Jia, X. Li, C. You, H. Liu, M. Liu, Y. Miao, H. Lin, J. Wang, Taming Interfacial Ion-Dipole Interactions With d-Orbital Delocalized Electron Catalysis Expediates Low-Temperature Li Metal Batteries. Adv. Mater. 38, e10894 (2025). 10.1002/adma.202510894 PMC12810596 41074733

[15] M. He, L. Zhu, Y. Liu, Y. Jia, Y. Hao, G. Ye, X. Hong, Z. Xiao, Y. Ma, J. Chen, M. B. Shafqat, Q. Pang, Highly Solvating Electrolytes with Core-Shell Solvation Structure for Lean-Electrolyte Lithium-Sulfur Batteries. Angew. Chem. Int. Ed. Engl. 64, e202415053 (2024). 10.1002/anie.202415053 39526580

[16] L. Liao, F. Wang, J. He, L. Ma, Q. Liu, Y. Deng, Ultrathin TA-MXene Modification Layer with Sub-Nanometer Interlayer Channels Suppresses Polysulfide Shuttling and Accelerates Li^+^ Desolvation. Adv. Funct. Mater. 36, e12612 (2025).

[17] F. Zhu, J. Wang, Y. Zhang, H. Tu, X. Xia, J. Zhang, H. He, H. Lin, M. Liu, Low-Temperature Lithium Metal Batteries Achieved by Synergistically Enhanced Screening Li^+^ Desolvation Kinetics. Adv. Mater. 37, e2411601 (2024). 10.1002/adma.202411601 PMC11795707 39679840

[18] J. Wang, J. Zhang, X. Cheng, Y. Zhang, H. Li, Q. Guan, F. Wu, H. Li, D. Wang, M. Liu, Y. Zhang, Q. Xiao, S. Passerini, H. Lin, Electrode/Electrolyte Interface Studies of Rechargeable Li Batteries with Interface-Specific Sum Frequency Generation Spectroscopy. J. Am. Chem. Soc. 147, 44633–44651 (2025). 10.1021/jacs.5c17560 41311262

[19] W. Weng, V. G. Pol, K. Amine, Ultrasound Assisted Design of Sulfur/Carbon Cathodes with Partially Fluorinated Ether Electrolytes for Highly Efficient Li/S Batteries. Adv. Mater. 25, 1608–1615 (2013). 10.1002/adma.201204051 23335003

[20] B. C. Xiong, M. S. B. Reddy, S. Das, W. Stasko, G. Freeman, V. G. Pol, Suppressing Shuttle Effect through Solvent Coordination and Nitrate Synergy Enables Stable Lithium-Sulfur Batteries. ACS Appl. Mater. Interfaces 18, 26105–26115 (2026). 10.1021/acsami.5c25388 42047298

[21] Y. H. Zhuang, H. Yang, Y. H. Li, Y. Zhao, H. H. Min, S. Cui, X. D. Shen, H. Y. Chen, Y. F. Wang, J. Wang, Curvature-Induced Electron Delocalization Activates the Bifunctional Catalytic Activity of COF/MXene for High-Performance Lithium-Sulfur Batteries. ACS Nano 19, 11058–11074 (2025). 10.1021/acsnano.4c17087 40068113

[22] K. Yang, F. Zhao, J. Li, H. Y. Yang, Y. C. Wang, Y. B. He, Designing Organic-Inorganic Hybrid Materials to Construct a Complementary Interface with Versatility for Li-S Batteries. Adv. Funct. Mater. 34, 2410236 (2024).

[23] C. L. Wei, Y. S. Wang, Y. C. Zhang, L. W. Tan, Y. Qian, Y. Tao, S. L. Xiong, J. K. Feng, Flexible and stable 3D lithium metal anodes based on self-standing MXene/COF frameworks for high-performance lithium-sulfur batteries. Nano Res. 14, 3576–3584 (2021).

[24] P. Dong, Y. Wang, A. Zhang, T. Cheng, X. Xi, J. Zhang, Platinum Single Atoms Anchored on a Covalent Organic Framework: Boosting Active Sites for Photocatalytic Hydrogen Evolution. Acs Catalysis 11, 13266–13279 (2021).

[25] T. Li, W. Liu, Y. Liu, J. Wang, H. Hao, Z. Yu, H. Liu, Covalent organic frameworks integrated MXene as selective “ion-sieving” heterostructure catalyst for kinetics-reinforced Li-S batteries. Chem. Eng. J. 498, 155817 (2024).

[26] Y. Lin, H. Cui, C. Liu, R. Li, S. Wang, G. Qu, Z. Wei, Y. Yang, Y. Wang, Z. Tang, H. Li, H. Zhang, C. Zhi, H. Lv, A covalent organic framework as a long-life and high-rate anode suitable for both aqueous acidic and alkaline batteries. Angew. Chem. Int. Ed. Engl. 62, e202218745 (2023). 10.1002/anie.202218745 36705089

[27] X. Yan, B. Wang, J. Ren, X. Long, D. Yang, An Unsaturated Bond Strategy to Regulate Active Centers of Metal-Free Covalent Organic Frameworks for Efficient Oxygen Reduction. Angew. Chem. Int. Ed. Engl. 61, e202209583 (2022). 10.1002/anie.202209583 36131487

[28] X. Kang, T. He, S. Niu, J. Zhang, R. Zou, F. Zhu, F. Ran, Precise Design of a 0.8 nm Pore Size in a Separator Interfacial Layer Inspired by a Sieving Effect toward Inhibiting Polysulfide Shuttling and Promoting Li^+^ Diffusion. Nano Lett. 24, 10007–10015 (2024). 10.1021/acs.nanolett.4c01480 39134477

[29] X. Dong, W. Gu, X. Tong, G. Liu, J. Sun, H. Li, X. Gu, T. Zhu, S. Zhang, In situ growth strategy to construct “four-in-one” separators with functionalized polyphosphazene coatings for safe and stable lithium-sulfur batteries. Small 20, e2311471 (2024). 10.1002/smll.202311471 38429237

[30] Z. Ye, Y. Jiang, L. Li, F. Wu, R. Chen, Enhanced catalytic conversion of polysulfide using 1D CoTe and 2D MXene for heat-resistant and lean-electrolyte Li-S batteries. Chem. Eng. J. 430, 132734 (2022).

[31] X. Wang, D. Luo, J. Wang, Z. Sun, G. Cui, Y. Chen, T. Wang, L. Zheng, Y. Zhao, L. Shui, G. Zhou, K. Kempa, Y. Zhang, Z. Chen, Strain Engineering of a MXene/CNT Hierarchical Porous Hollow Microsphere Electrocatalyst for a High-Efficiency Lithium Polysulfide Conversion Process. Angew. Chem. Int. Ed. Engl. 60, 2371–2378 (2021). 10.1002/anie.202011493 33398902

[32] C. N. Zhu, C. C. Sun, R. H. Li, S. T. Weng, L. W. Fan, X. F. Wang, L. X. Chen, M. Noked, X. L. Fan, Anion-Diluent Pairing for Stable High-Energy Li Metal Batteries. ACS Energy Lett. 7, 1338–1347 (2022).

[33] Y. Jin, P. M. L. Le, P. Y. Gao, Y. B. Xu, B. W. Xiao, M. H. Engelhard, X. Cao, T. D. Vo, J. T. Hu, L. R. Zhong, B. E. Matthews, R. Yi, C. M. Wang, X. L. Li, J. Liu, J.-G. Zhang, Low-solvation electrolytes for high-voltage sodium-ion batteries. Nat. Energy 7, 718–725 (2022).

[34] X. Zuo, L. Wang, M. Zhen, T. You, D. Liu, Y. Zhang, Multifunctional TiN-MXene-Co@CNTs networks as sulfur/lithium host for high-areal-capacity lithium-sulfur batteries. Angew. Chem. Int. Ed. Engl. 63, e202408026 (2024). 10.1002/anie.202408026 38867467

[35] Z. Li, P. Li, X. Meng, Z. Lin, R. Wang, The Interfacial Electronic Engineering in Binary Sulfiphilic Cobalt Boride Heterostructure Nanosheets for Upgrading Energy Density and Longevity of Lithium-Sulfur Batteries. Adv. Mater. 33, e2102338 (2021). 10.1002/adma.202102338 34480377

[36] D. Yang, M. Li, X. Zheng, X. Han, C. Zhang, J. Jacas Biendicho, J. Llorca, J. Wang, H. Hao, J. Li, G. Henkelman, J. Arbiol, J. Ramon Morante, D. Mitlin, S. Chou, A. Cabot, Phase engineering of defective copper selenide toward robust lithium-sulfur batteries. ACS Nano 16, 11102–11114 (2022). 10.1021/acsnano.2c03788 35758405

[37] Z. Jin, T. Lin, H. Jia, B. Liu, Q. Zhang, L. Li, L. Zhang, Z.-M. Su, C. Wang, Expediting the Conversion of Li_2_S_2_ to Li_2_S Enables High-Performance Li-S Batteries. ACS Nano 15, 7318–7327 (2021). 10.1021/acsnano.1c00556 33784808

[38] M. Zhen, X. Wang, Q. Yang, Z. Zhang, Z. Hu, Z. Li, Z. Wang, Nitrogen-Doped TiO_2-x_(B)/MXene Heterostructures for Expediting Sulfur Redox Kinetics and Suppressing Lithium Dendrites. Adv. Sci. 11, e2406475 (2024). 10.1002/advs.202406475 PMC11423229 39041888

[39] W. Cai, G. Li, K. Zhang, G. Xiao, C. Wang, K. Ye, Z. Chen, Y. Zhu, Y. Qian, Conductive Nanocrystalline Niobium Carbide as High-Efficiency Polysulfides Tamer for Lithium-Sulfur Batteries. Adv. Funct. Mater. 28, 1704865 (2017).

[40] Z. Li, F. Zhang, T. Cao, L. Tang, Q. Xu, H. Liu, Y. Wang, Highly stable lithium-sulfur batteries achieved by a SnS/porous carbon nanosheet architecture modified celgard separator. Adv. Funct. Mater. 30, 2006297 (2020).

[41] B. Wang, Y. Ren, Y. Zhu, S. Chen, S. Chang, X. Zhou, P. Wang, H. Sun, X. Meng, S. Tang, Construction of Co_3_O_4_/ZnO heterojunctions in hollow N-Doped carbon nanocages as microreactors for lithium-sulfur full batteries. Adv. Sci. 10, e2300860 (2023). 10.1002/advs.202300860 PMC10323615 37078796

[42] C. Dong, C. Ma, C. Zhou, Y. Yu, J. Wang, K. Yu, C. Shen, J. Gu, K. Yan, A. Zheng, M. Gong, X. Xu, L. Mai, Engineering d-p Orbital Hybridization with P, S Co-Coordination Asymmetric Configuration of Single Atoms Toward High-Rate and Long-Cycling Lithium-Sulfur Battery. Adv. Mater. 36, e2407070 (2024). 10.1002/adma.202407070 39091051

[43] J. Feng, C. Zhang, W. Liu, S. Yu, L. Wang, T. Wang, C. Shi, X. Zhao, S. Chen, S. Chou, J. Song, Enabling Efficient Anchoring-Conversion Interface by Fabricating Double-Layer Functionalized Separator for Suppressing Shuttle Effect. Angew. Chem. Int. Ed. Engl. 63, e202407042 (2024). 10.1002/anie.202407042 39004938

[44] Z. Li, Y. Zhou, Y. Wang, Y.-C. Lu, Solvent-Mediated Li_2_S Electrodeposition: A Critical Manipulator in Lithium-Sulfur Batteries. Adv. Energy Mater. 9, 1802207 (2019).

[45] F. Y. Fan, W. C. Carter, Y.-M. Chiang, Mechanism and Kinetics of Li_2_S Precipitation in Lithium-Sulfur Batteries. Adv. Mater. 27, 5203–5209 (2015). 10.1002/adma.201501559 26257297

[46] K. Yang, F. Zhao, J. Li, H. Yang, Y. Wang, Y. He, Designing Organic-Inorganic Hybrid Materials to Construct a Complementary Interface with Versatility for Li-S Batteries. Adv. Funct. Mater. 34, 2410236 (2024).

[47] Q. Liu, X. Han, Z. Zheng, P. Xiong, R.-G. Jeong, G. Kim, H. Park, J. Kim, B.-K. Kim, H. S. Park, Crystallinity Regulated Functional Separator Based on Bimetallic Ni_x_Fe_y_ Alloy Nanoparticles for Facilitated Redox Kinetics of Lithium-Sulfur Batteries. Adv. Funct. Mater. 32, 2207094 (2022).

[48] T. Li, H. Hao, J. Wang, Z. Yuan, Z. Yu, H. Liu, Engineering covalent organic frameworks to enhance interfacial lithium-ion flux redistribution in dendrite-free lithium-sulfur batteries. J. Mater. Chem. A 13, 7939–7948 (2025).

[49] T. Li, C. Yang, H. Hao, Z. Yuan, Z. Yu, H. Liu, MXene porous engineering as lithium-ion kinetic pump for durable lithium-sulfur batteries. J. Alloys Compd. 1022, 180032 (2025).

[50] S. Chen, J. Luo, N. Li, X. Han, J. Wang, Q. Deng, Z. Zeng, S. Deng, Multifunctional LDH/Co_9_S_8_ heterostructure nanocages as high-performance lithium-sulfur battery cathodes with ultralong lifespan. Energy Storage Mater. 30, 187–195 (2020).

[51] M. Zheng, C. Guo, Z. Luo, J. Wu, X. Tang, L. Li, Q. Sun, Q. Ouyang, B. Shi, H. Nie, J.-J. Shao, G. Zhou, Molybdenum disulfide (MoS_2_)/porous silica nanosheet composite barrier for polysulfide shuttling inhibition in lithium-sulfur batteries. Compos. B: Eng. 264, 110898 (2023).

[52] J. Xu, S. An, X. Song, Y. Cao, N. Wang, X. Qiu, Y. Zhang, J. Chen, X. Duan, J. Huang, W. Li, Y. Wang, Towards High Performance Li-S Batteries via Sulfonate-Rich COF-Modified Separator. Adv. Mater. 33, e2105178 (2021). 10.1002/adma.202105178 34622528

[53] B. Zhang, C. Luo, G. Zhou, Z.-Z. Pan, J. Ma, H. Nishihara, Y.-B. He, F. Kang, W. Lv, Q.-H. Yang, Lamellar MXene Composite Aerogels with Sandwiched Carbon Nanotubes Enable Stable Lithium-Sulfur Batteries with a High Sulfur Loading. Adv. Funct. Mater. 31, 2100793 (2021).

[54] Y. Zhang, J. Liu, J. Wang, Y. Zhao, D. Luo, A. Yu, X. Wang, Z. Chen, Engineering oversaturated Fe-N_5_ multifunctional catalytic sites for durable lithium-sulfur batteries. Angew. Chem. Int. Ed. Engl. 60, 26622–26629 (2021). 10.1002/anie.202108882 34463010

[55] L. Zuo, Q. Ma, P. Xiao, Q. Guo, W. Xie, D. Lu, X. Yun, C. Zheng, Y. Chen, Upgrading the Separators Integrated with Desolvation and Selective Deposition toward the Stable Lithium Metal Batteries. Adv. Mater. 36, e2311529 (2024). 10.1002/adma.202311529 38154114

[56] H. Tang, N. Hu, L. Ma, H. Weng, D. Huang, J. Zhu, H. Yang, Z. Chen, X. Yin, J. Xu, H. He, Interfacial Dual-Modulation via Cationic Electrostatic Shielding and Anionic Preferential Adsorption toward Planar and Reversible Zinc Electrodeposition. Adv. Funct. Mater. 34, 2402484 (2024).

[57] Y. Chen, K. Zou, X. Dai, H. Bai, S. Zhang, T. Zhou, C. Li, Y. Liu, W. K. Pang, Z. Guo, Polysulfide filter and dendrite inhibitor: highly graphitized wood framework inhibits polysulfide shuttle and lithium dendrites in Li-S batteries. Adv. Funct. Mater. 31, 2102458 (2021).

[58] X. Dong, W. Gu, X. Tong, G. Liu, J. Sun, H. Li, X. Gu, T. Zhu, S. Zhang, In Situ Growth Strategy to Construct “Four-In-One” Separators with Functionalized Polyphosphazene Coatings for Safe and Stable Lithium-Sulfur Batteries. Small 20, e2311471 (2024). 10.1002/smll.202311471 38429237

[59] J. Xu, A. Zhu, Z. Zheng, Y. Qi, Y. Cheng, Y. Cao, B. Peng, L. Ma, Y. Wang, Building Li-S batteries with enhanced temperature adaptability via a redox-active COF-based barrier-trapping electrocatalyst. J. Energy Chem. 101, 702–712 (2025).

[60] C.-X. Bi, N. Yao, X.-Y. Li, Q.-K. Zhang, X. Chen, X.-Q. Zhang, B.-Q. Li, J.-Q. Huang, Unveiling the Reaction Mystery Between Lithium Polysulfides and Lithium Metal Anode in Lithium-Sulfur Batteries. Adv. Mater. 36, e2411197 (2024). 10.1002/adma.202411197 39149771

[61] Y. Zhang, C. Kang, W. Zhao, Y. Song, J. Zhu, H. Huo, Y. Ma, C. Du, P. Zuo, S. Lou, G. Yin, D-P hybridization-induced “trapping-coupling-conversion” enables high-efficiency Nb single-atom catalysis for Li-S batteries. J. Am. Chem. Soc. 145, 1728–1739 (2023). 10.1021/jacs.2c10345 36640116

[62] J. Xu, L. Xu, Z. Zhang, B. Sun, Y. Jin, Q. Jin, H. Liu, G. Wang, Heterostructure ZnSe-CoSe_2_ embedded with yolk-shell conductive dodecahedral as two-in-one hosts for cathode and anode protection of lithium-sulfur full batteries. Energy Storage Mater. 47, 223–234 (2022).

[63] H. Wang, H. Guo, Z. Huang, W. Liu, M. Li, J. Yao, J. Cui, Y. Wang, M. Ren, Bidirectional enhancement of Li_2_S redox reaction by NiSe_2_/CoSe_2_-rGO heterostructured bi-functional catalysts. J. Colloid Interface Sci. 660, 458–468 (2024). 10.1016/j.jcis.2024.01.115 38246049

[64] G. Liu, Q. Zeng, Z. Fan, S. Tian, X. Li, X. Lv, W. Zhang, K. Tao, E. Xie, Z. Zhang, Boosting sulfur catalytic kinetics by defect engineering of vanadium disulfide for high-performance lithium-sulfur batteries. Chem. Eng. J. 448, 137683 (2022).

[65] J.-L. Yang, S.-X. Zhao, Y.-M. Lu, X.-T. Zeng, W. Lv, G.-Z. Cao, In-situ topochemical nitridation derivative MoO_2_–Mo_2_N binary nanobelts as multifunctional interlayer for fast-kinetic Li-Sulfur batteries. Nano Energy 68, 104356 (2020).

[66] X. Dong, W. Gu, X. Tong, G. Liu, J. Sun, H. Li, X. Gu, T. Zhu, S. Zhang, In Situ Growth Strategy to Construct “Four-In-One” Separators with Functionalized Polyphosphazene Coatings for Safe and Stable Lithium-Sulfur Batteries. Small 20, e2311471 (2024). 10.1002/smll.202311471 38429237

[67] L. Ma, Y. Zhang, S. Zhang, L. Wang, C. Zhang, Y. Chen, Q. Wu, L. Chen, L. Zhou, W. Wei, Integrating Energy Band Alignment and Oxygen Vacancies Engineering of TiO_2_ Anatase/Rutile Homojunction for Kinetics-Enhanced Li-S Batteries. Adv. Funct. Mater. 33, 2305788 (2023).

[68] L. Wu, C. Cai, X. Yu, Z. Chen, Y. Hu, F. Yu, S. Zhai, T. Mei, L. Yu, X. Wang, Scalable 3D Honeycombed Co_3_O_4_ Modified Separators as Polysulfides Barriers for High-Performance Li–S Batteries. ACS Appl. Mater. Interfaces 14, 35894–35904 (2022). 10.1021/acsami.2c07263 35881975

[69] H. Li, Q. Jin, D. Li, X. Huan, Y. Liu, G. Feng, J. Zhao, W. Yang, Z. Wu, B. Zhong, X. Guo, B. Wang, Mo_2_C-Embedded Carambola-like N, S-Rich Carbon Framework as the Interlayer Material for High-Rate Lithium–Sulfur Batteries in a Wide Temperature Range. ACS Appl. Mater. Interfaces 12, 22971–22980 (2020). 10.1021/acsami.0c04842 32347703

