# Supplementary material for: Nanoconfinement-cooperated catalysis enables rapid tandem redox kinetics for wide-temperature lithium-sulfur batteries
Source: Sci Adv. 2026 Oct 14;12(42):eaej0353. doi: 10.1126/sciadv.aej0353 (PMC13665964; doi:10.1126/sciadv.aej0353)
Supplement: Supplementary file 1 [file sciadv.aej0353_sm.pdf]

Science Advances
AAAS

Supplementary Materials for

Nanoconfinement-cooperated catalysis enables rapid tandem redox kinetics for wide-temperature lithium-sulfur batteries

Tianli Li *et al.*

Corresponding author: Pu Hu, hupu@wit.edu.cn; Hanxing Liu, lhxhp@whut.edu.cn; Jian Wang, jian.wang@kit.edu; Yaobing Wang, wangyb@fjirsm.ac.cn

Sci. Adv. **12**, eaej0353 (2026)
DOI: 10.1126/sciadv.aej0353

This PDF file includes:

Supplementary Text
Figs. S1 to S48
Tables S1 to S3
References

Materials characterization

X-ray diffraction (XRD, Bruker D8) was utilized to analyze the crystalline phases of the as-fabricated materials. Structural microphotographs and elemental mapping were acquired using transmission electron microscopy (TEM, Talos-F200S) and scanning electron microscopy (SEM, JSM-IT300). Ultraviolet-visible (UV-vis) spectrophotometer (Perkin Elmer Lambda) was used to study the absorption spectra of diverse materials. The Raman spectra of the samples were evaluated using a Raman spectrometer (Raman, InVia). TOF-SIMS (Time-of-Flight Secondary Ion Mass Spectrometry) (ION-TOF GmbH) was employed to visualize the three-dimensional spatial distribution of polysulfides. The specific surface area was determined through the analysis of N_2 adsorption-desorption isotherms on an ASAP 2460 instrument. The elemental composition, chemical bonds, and valence states within the samples were analyzed utilizing XPS, ESCALAB-250Xi instrument. The contact angle of various separators was evaluated by employing a contact angle instrument (JC2000C optical).

Theoretical calculations

The first-principles density functional theory (DFT) calculations were carried out using the Vienna Ab initio simulation package (VASP). The exchange-correlation effects were depicted by the Perdew-Burke-Ernzerhof (PBE) functional within the generalized gradient approximation (GGA) strategy. The core-valence interactions were described using the projector augmented wave (PAW) method. After convergence testing, 450 eV cut-off value was set for plane-wave basis expansion. The force convergence threshold was set at 0.02 eV/Å for geometric optimization. A 15 Å vacuum layer was established along the Z direction to prevent any interactions between periodic images. The DFT-D3 methodology was employed to account for the van der Waals interactions. The charge density difference was derived through calculating the charge difference between adsorbent and substrate. The calculation details of the Gibbs free energy change (ΔG) were defined by the following equation (*52*):

$$\Delta G = \Delta E + \Delta ZPE - T\Delta S$$

where ΔE denotes the electronic energy difference between the free-standing and

the adsorbed states of the intermediates, ΔZPE represents the zero-point energy difference, ΔS stands for the variation of entropy, T is the room temperature. The binding energy between S_8 and Li_2S_x (x = 8, 6, 4, 2, 1) was calculated by the following equation:

$$E_{\mathrm{ads}} = E_{\mathrm{ad/sub}} - E_{\mathrm{ad}} - E_{\mathrm{sub}}$$

where $E_{\mathrm{ad/sub}}$ represents the total energy of the Li_2S_x adsorbed systems, E_{ad} and E_{sub} are the energies of the surface and the isolated Li_2S_x molecule.

Molecular dynamics (MD) investigations were conducted using the Forcite module, wherein the electrolyte composition was defined by a 1:5:7 molar ratio of LiTFSI/DME/DOL to replicate a 1 M concentration (*53*). The modeled architecture comprised 100 LiTFSI units, 500 DME molecules, and 700 DOL molecules within a cubic cell of 55.9 Å at 308 K. Interatomic interactions were described using the COMPASS III force field with a non-bonded cutoff distance of 15.5 Å. To attain thermodynamic stability, the system underwent an initial 1 ns equilibration under the isothermal-isobaric (NPT) ensemble to normalize density and box volume, followed by a 5 ns duration in the canonical (NVT) ensemble. Data regarding the Li-O radial distribution function (RDF) (g(r)) and coordination number (n(r)) were extracted from the stabilized NVT trajectories.

$$\mathrm{g}(r) = \frac{dn(\mathrm{r})}{4\pi \mathrm{d}r\rho r}$$

$$\mathrm{n}(r) = \int_0^r 4\pi r^2 \mathrm{g}(r)\, \rho(r)\, \mathrm{d}r$$

where the CN is quantified based on the local atomic density ρ(r). The same computational parameters and equilibration protocols were strictly maintained for the analysis of the TPBD-electrolyte solid-liquid interface to ensure methodological uniformity.

COMSOL Multiphysics simulations

To perform the finite element simulations, COMSOL Multiphysics was utilized, specifically employing the "Tertiary Current Distribution" interface to resolve the potential distribution, current density, and ion concentration fields. The transport of

ionic species was governed by Fick's first law for diffusion and the Nernst-Planck relationship for electromigration (*54*).

$$\frac{\partial c_i}{\partial t} = \nabla \bullet \left(D_i \nabla c_i + z_i u_{m,j} F c_i \nabla_{\varphi} \right)$$

$$u_{m,j} = \frac{D_i}{RT}$$

In the numerical model, c_i represents the electrolyte ion concentration, t denotes time, and D_i, z_i, and u_i refer to the diffusion coefficient, charge number, and mobility of the respective ionic species, respectively. F is the Faraday constant, φ is the polarization voltage, R is the universal gas constant, and T is the absolute temperature.

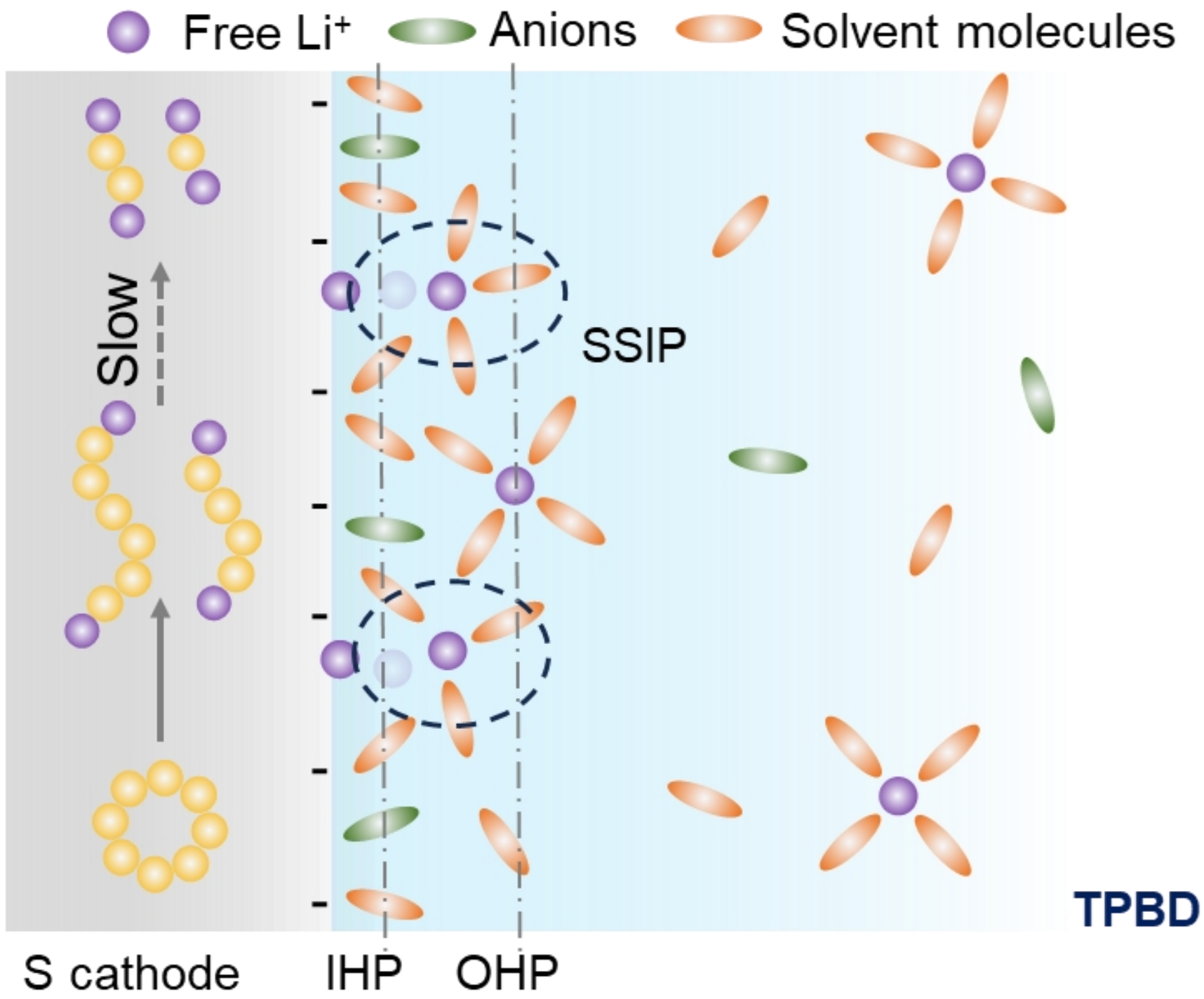

Fig. S1. Schematic of Li^+ desolvation shell evolution of TPBD.

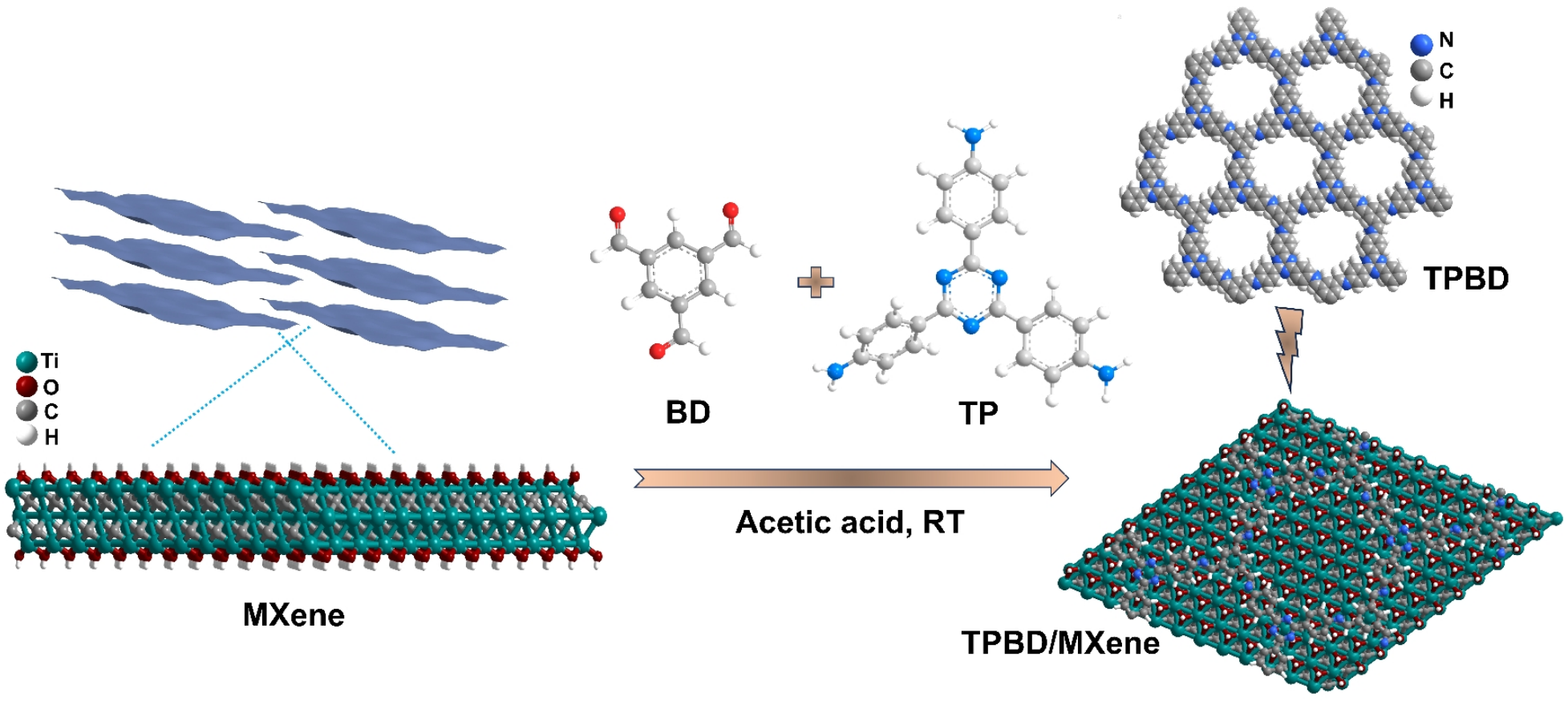

Fig. S2. The manufacturing process of TPBD/MXene hybrid material.

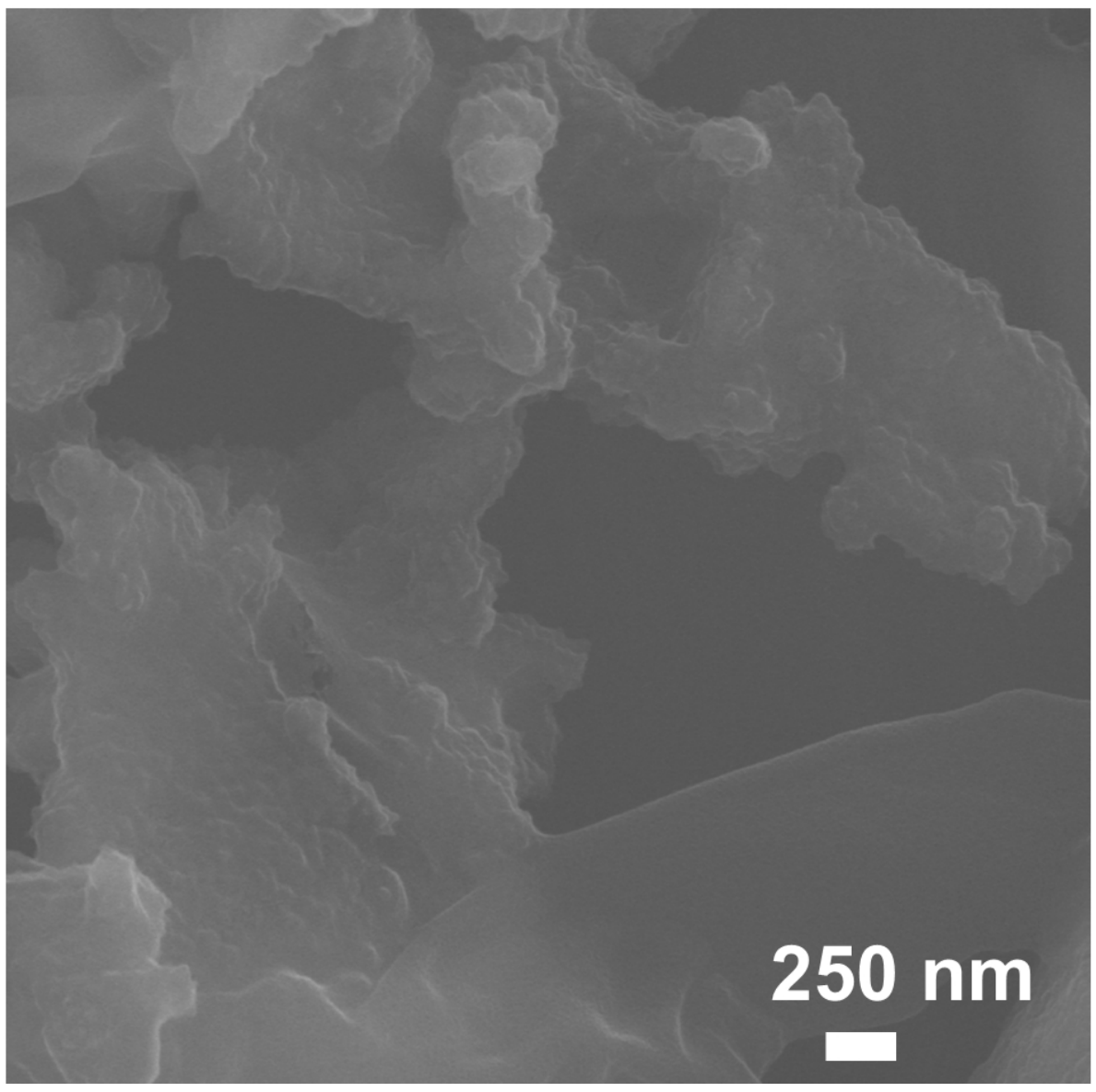

Fig. S3. The morphological characterizations. SEM image of the TPBD/MXene hybrid material.

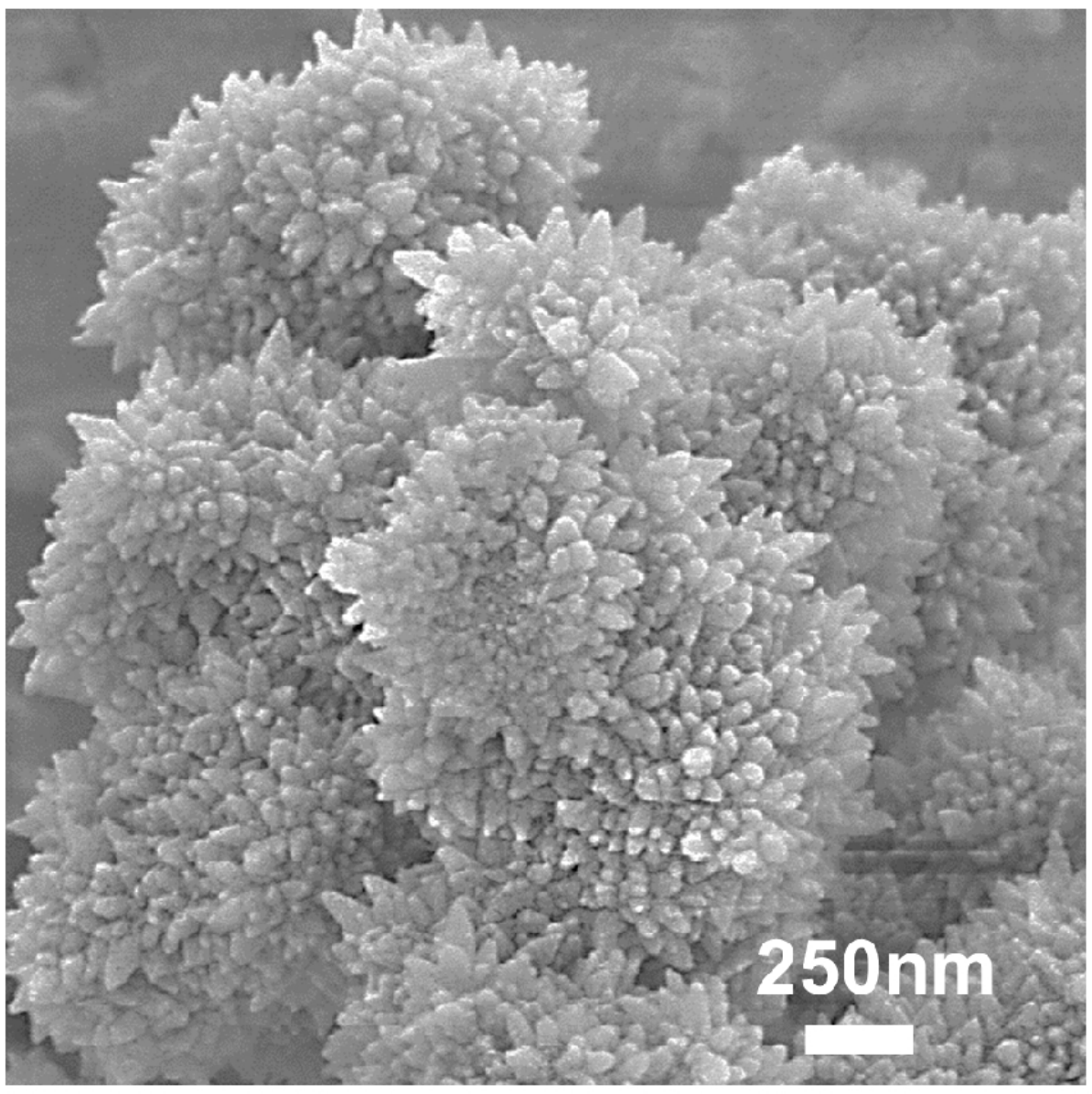

Fig. S4. The morphological characterizations. SEM image of the TPBD material.

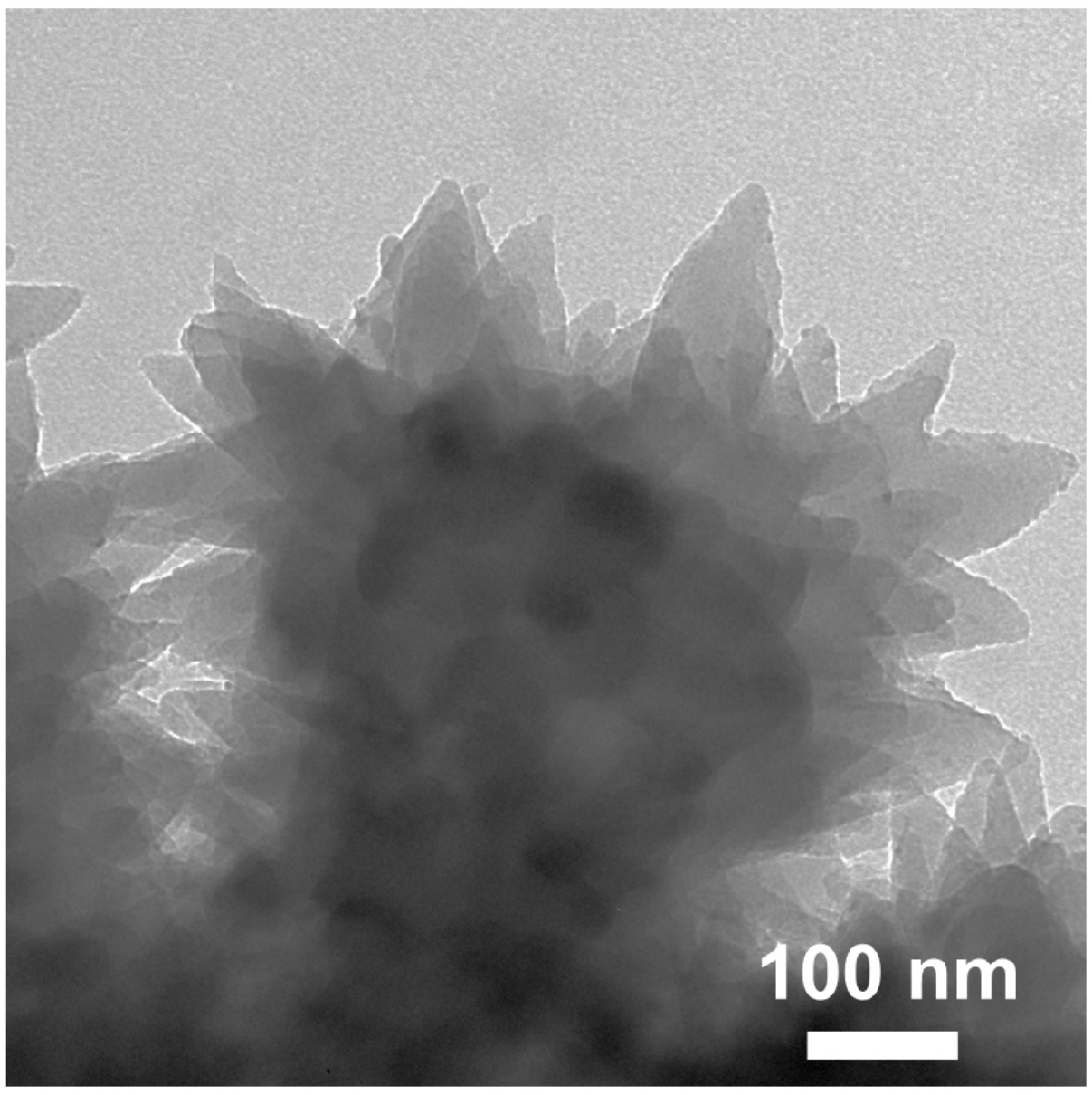

Fig. S5. The morphological characterizations. TEM image of the TPBD material.

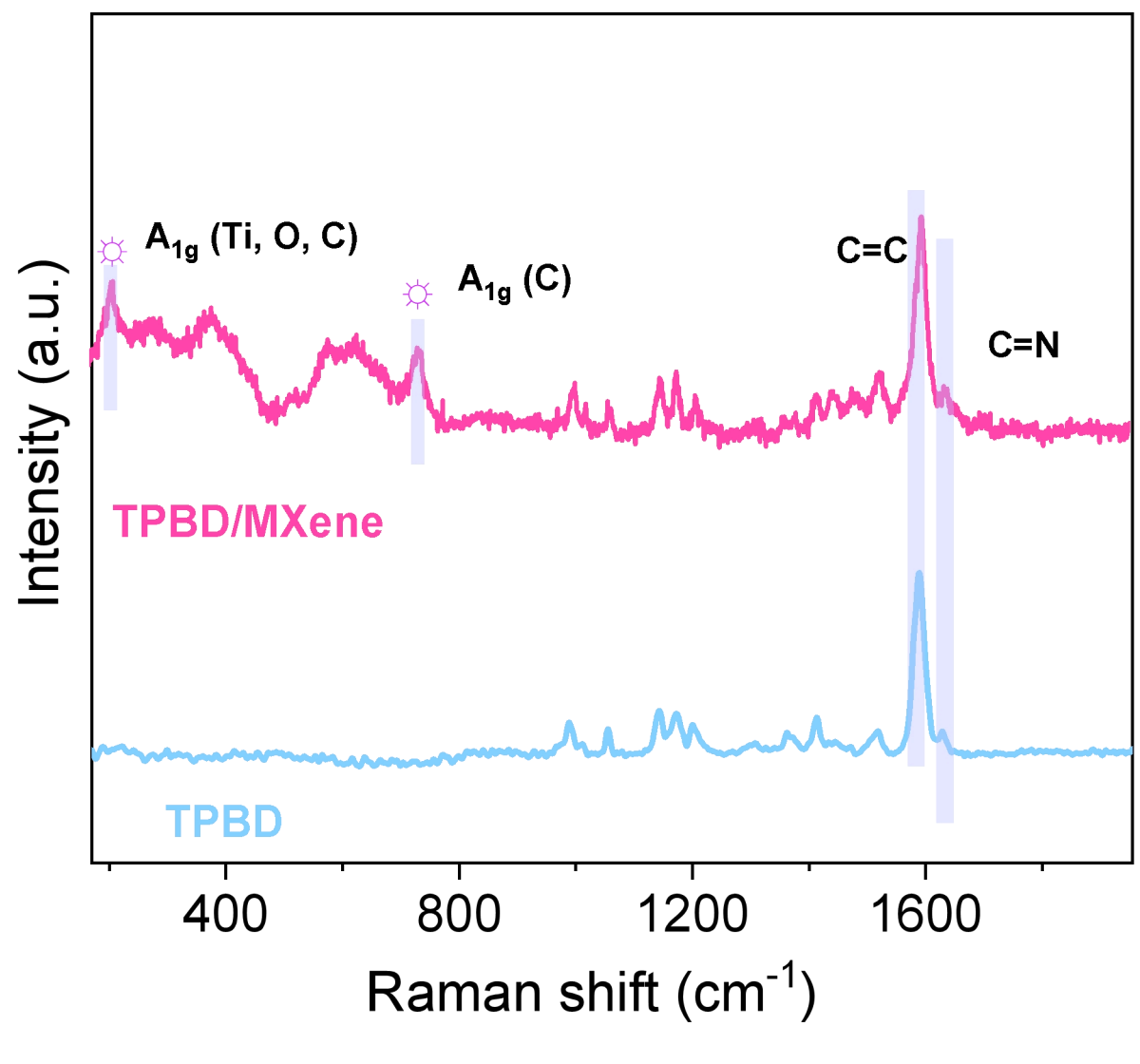

Fig. S6. The structural characterizations. Raman spectroscopy of TPBD/MXene.

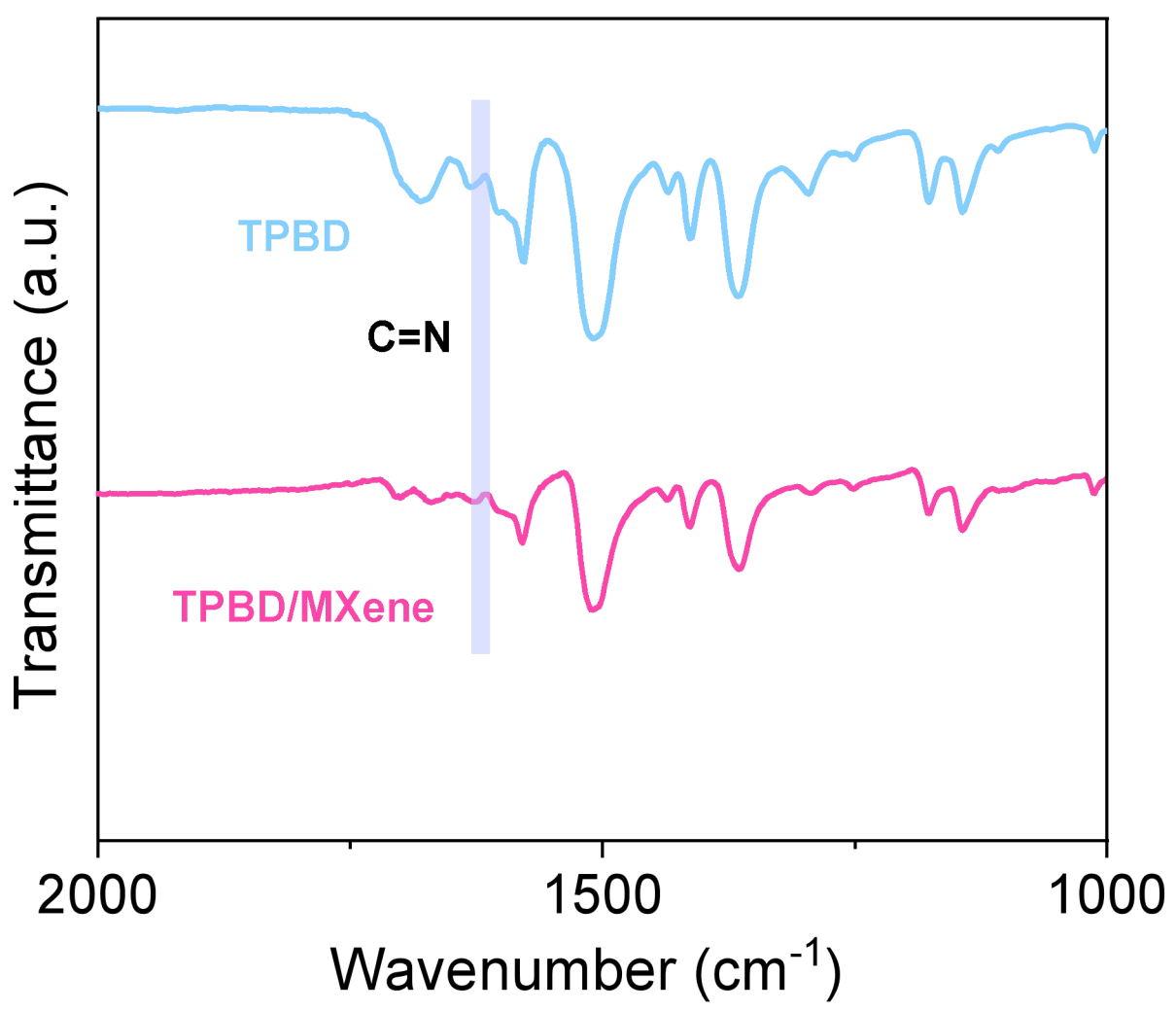

Fig. S7. **The structural characterizations.** FTIR patterns of TPBD/MXene.

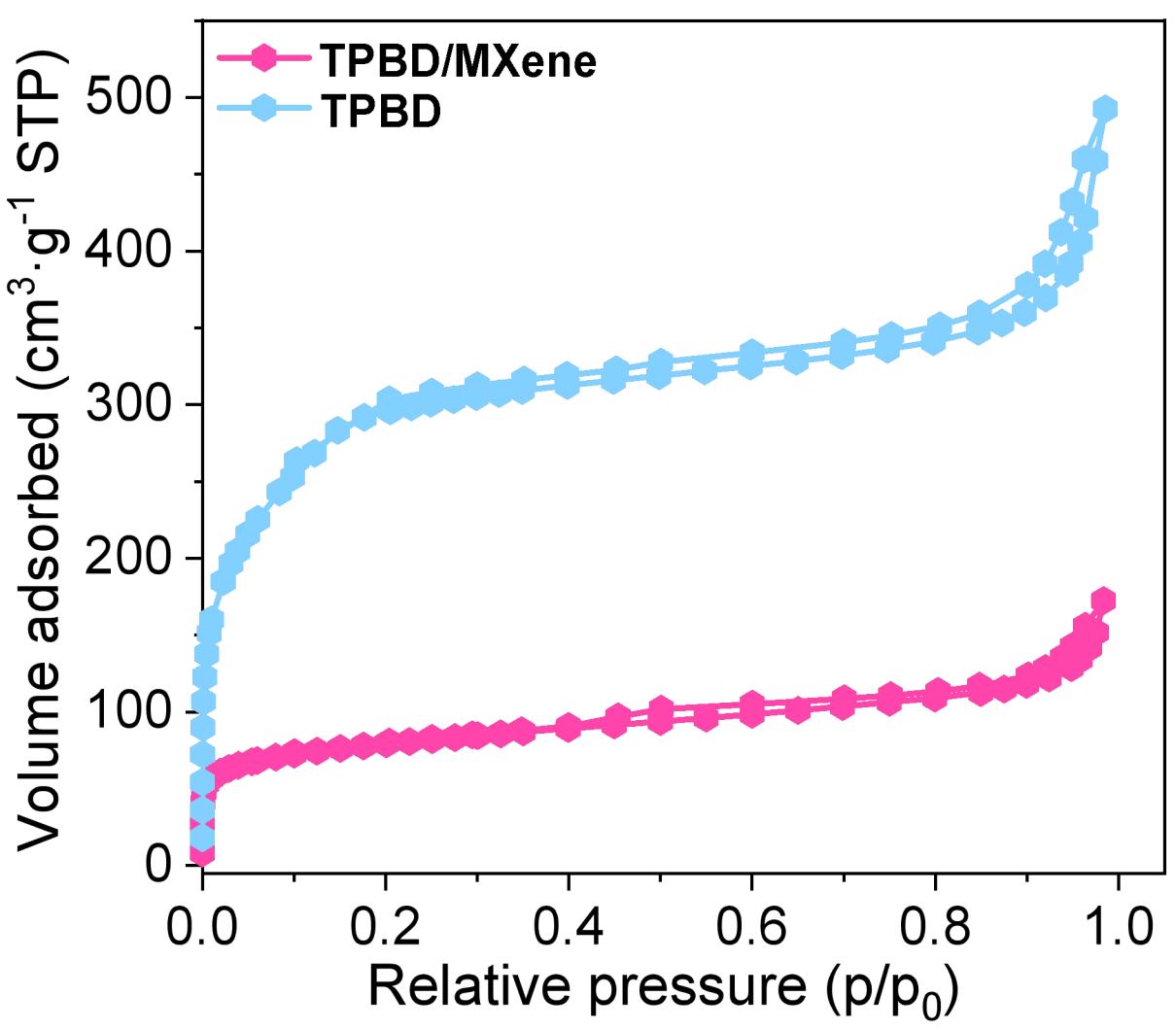

Fig. S8. N_2 adsorption-desorption isotherms. N_2 adsorption and desorption profiles of TPBD/MXene and TPBD.

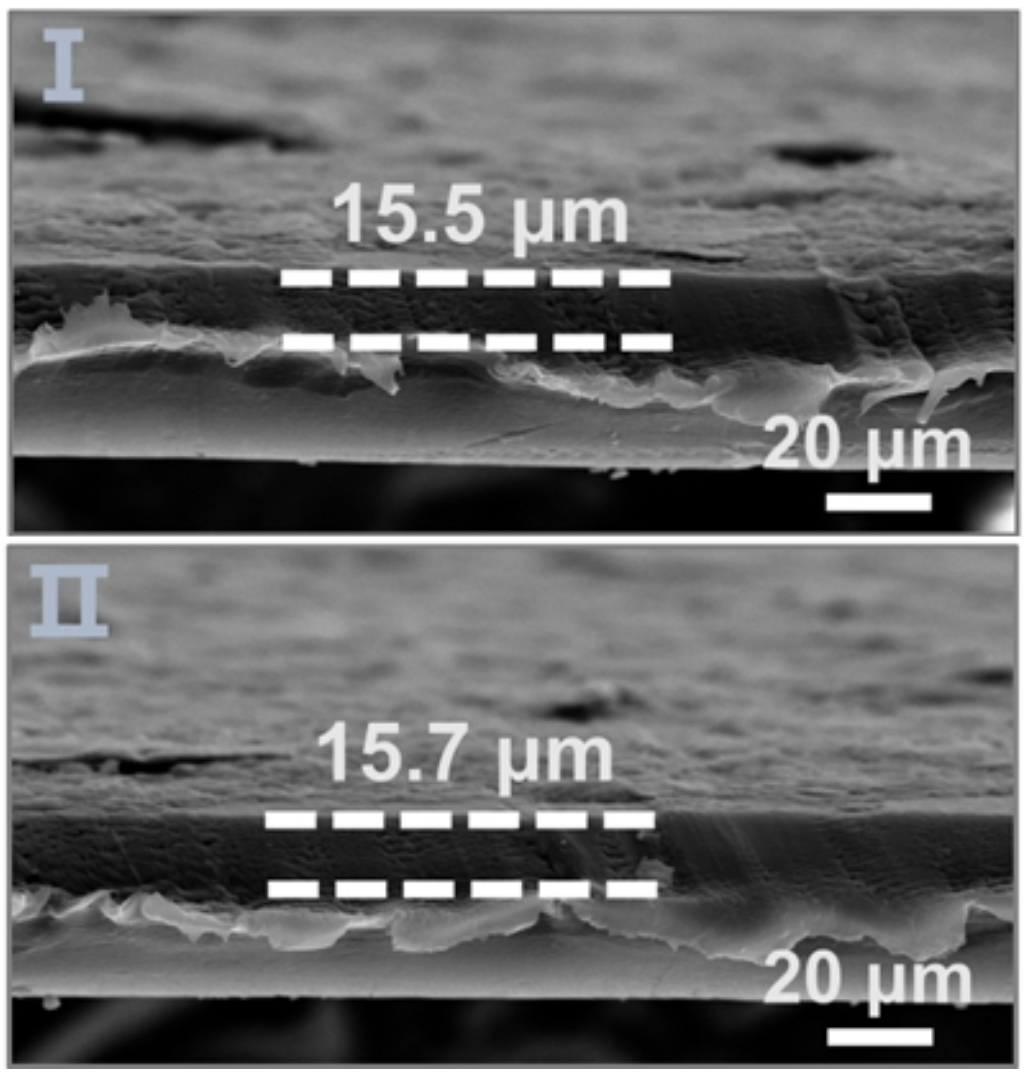

Fig. S9. The morphological characterizations. Cross-sectional SEM images of **(I)** TPBD/MXene and **(II)** TPBD.

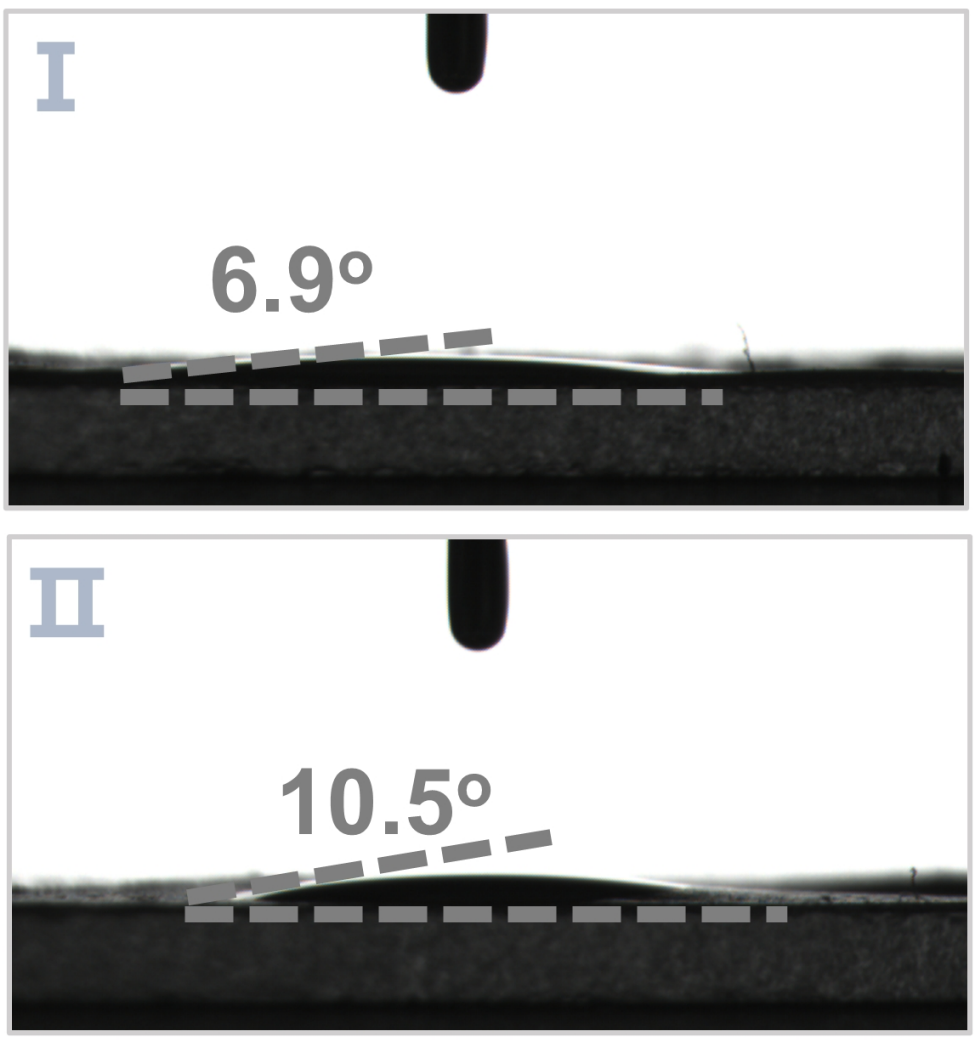

Fig. S10. Contact angle results. Contact angle test results of **(I)** TPBD/MXene and **(II)** TPBD.

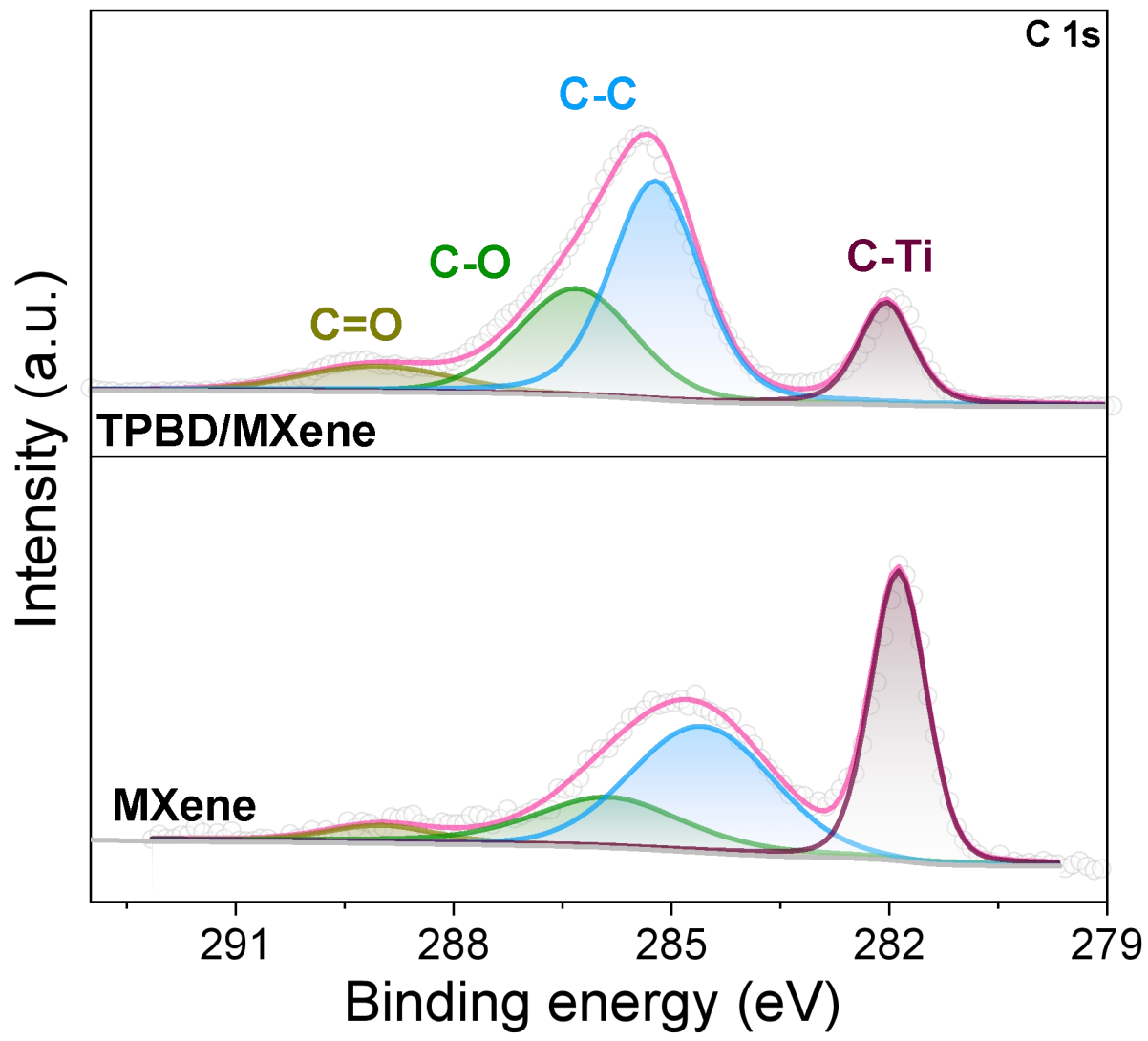

Fig. S11. C 1s XPS spectra of TPBD/MXene and MXene materials.

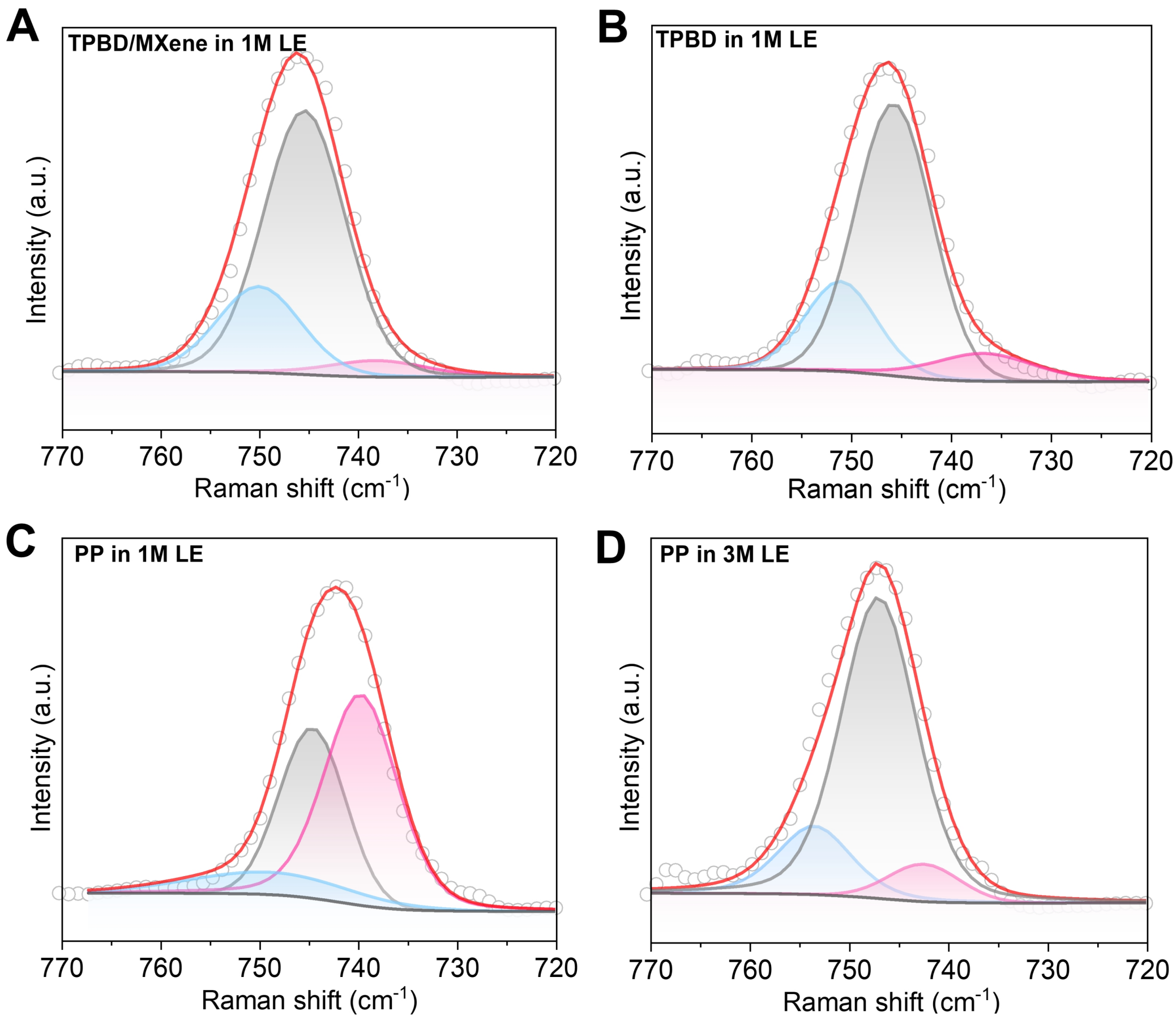

Fig. S12. Raman analysis. Raman spectra and fitting results of **(A)** TPBD/MXene in 1M LE, **(B)** TPBD in 1M LE, **(C)** PP in 1M LE, **(D)** PP in 3M LE.

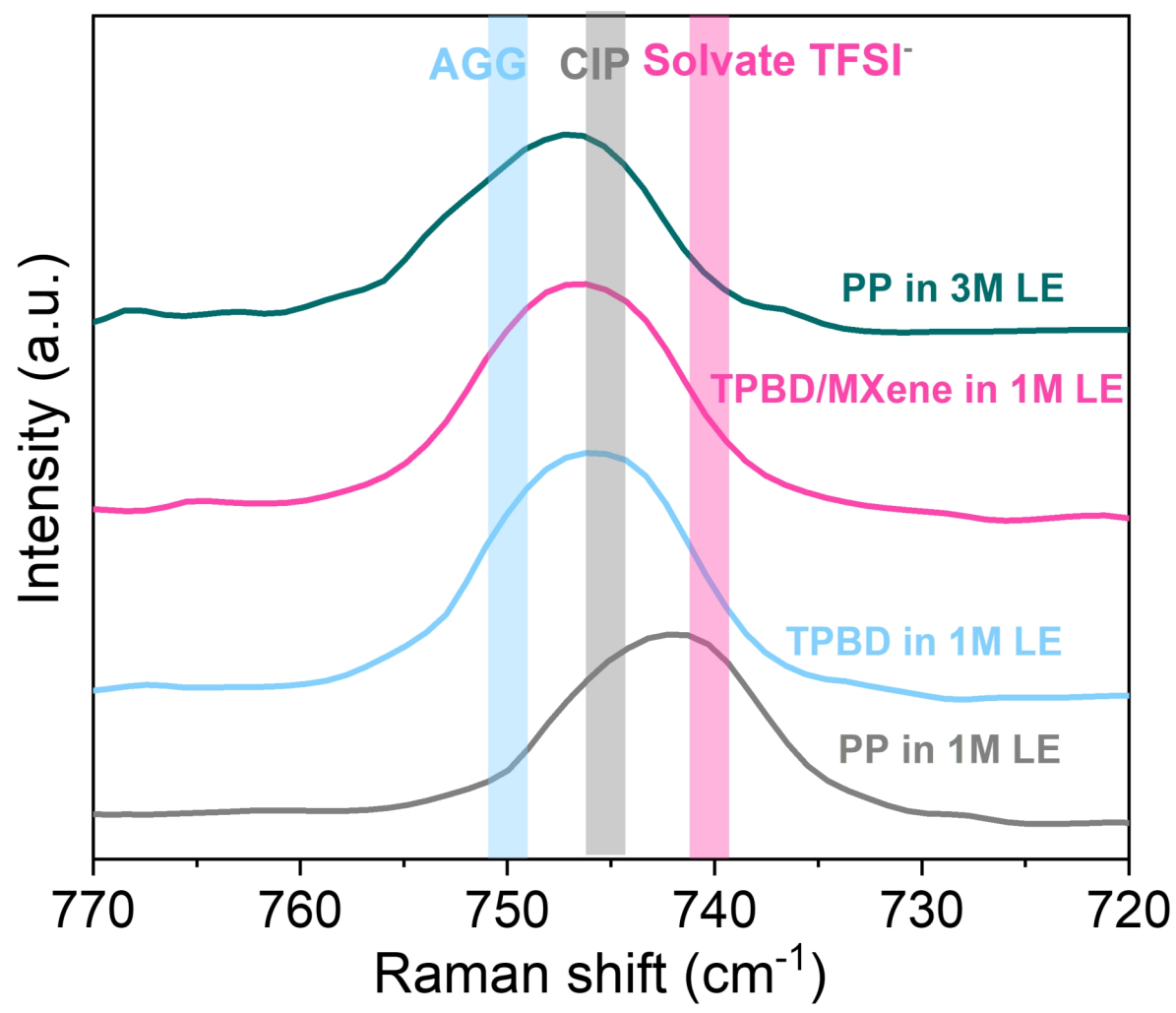

Fig. S13. Raman comparison of ether electrolyte systems. Raman spectral comparison for the ether electrolyte with versus without modified separator.

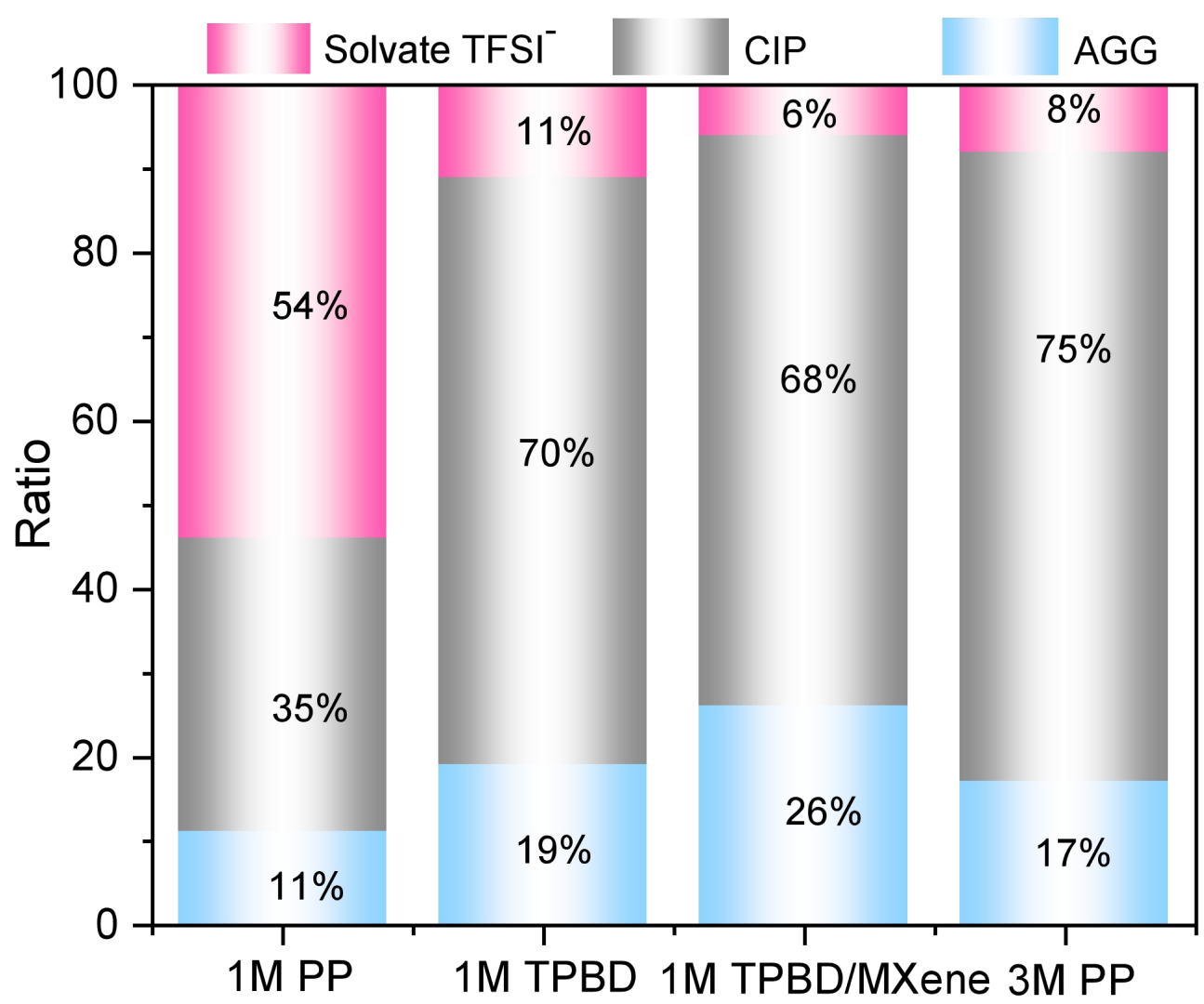

Fig. S14. Ionic species distribution across different systems. Quantitative distribution of free $TFSI^-$, CIPs, and AGGs across the investigated systems.

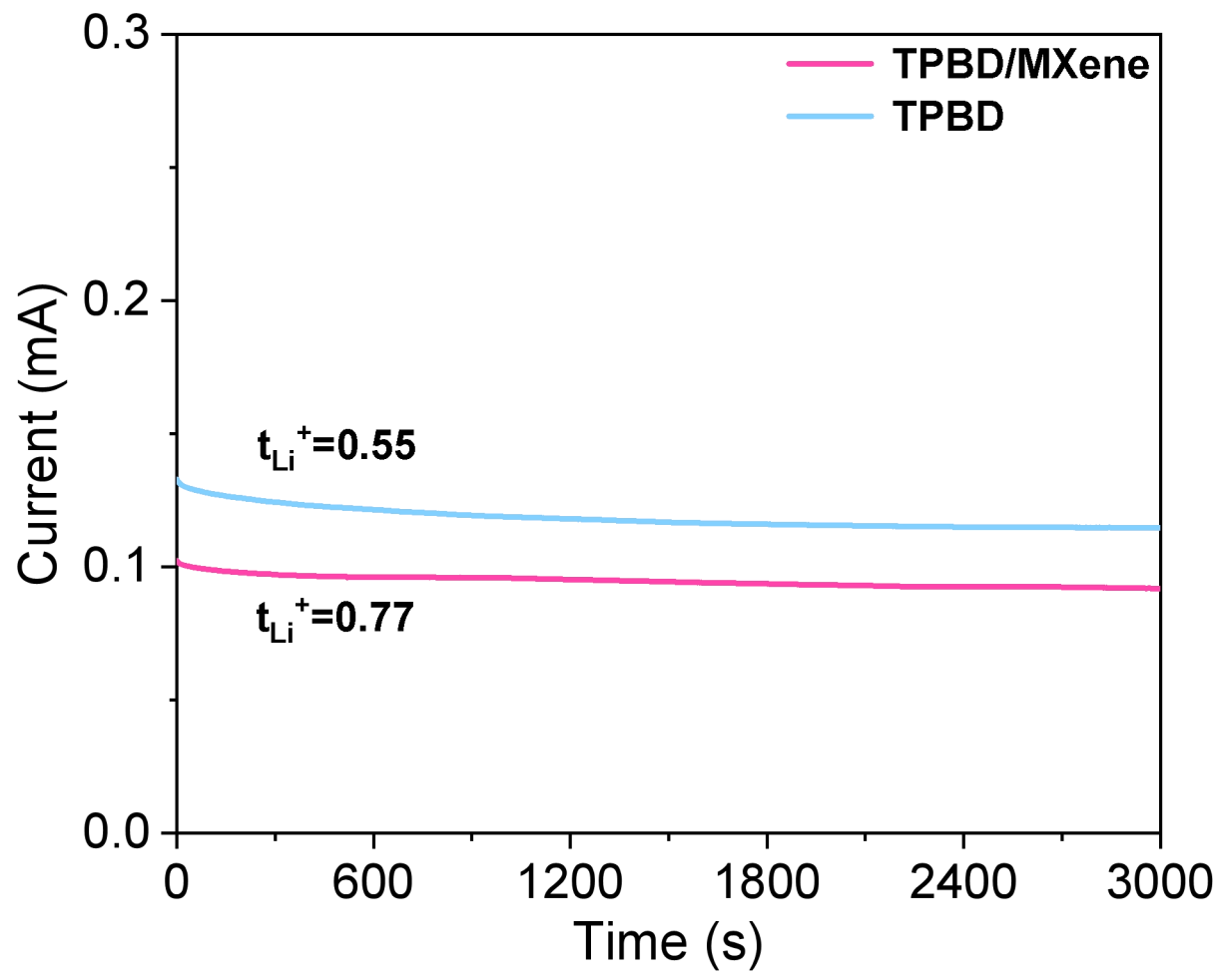

Fig. S15. Li⁺ transference number comparison. The Li⁺ transference number in batteries employing TPBD/MXene and TPBD-modified separators.

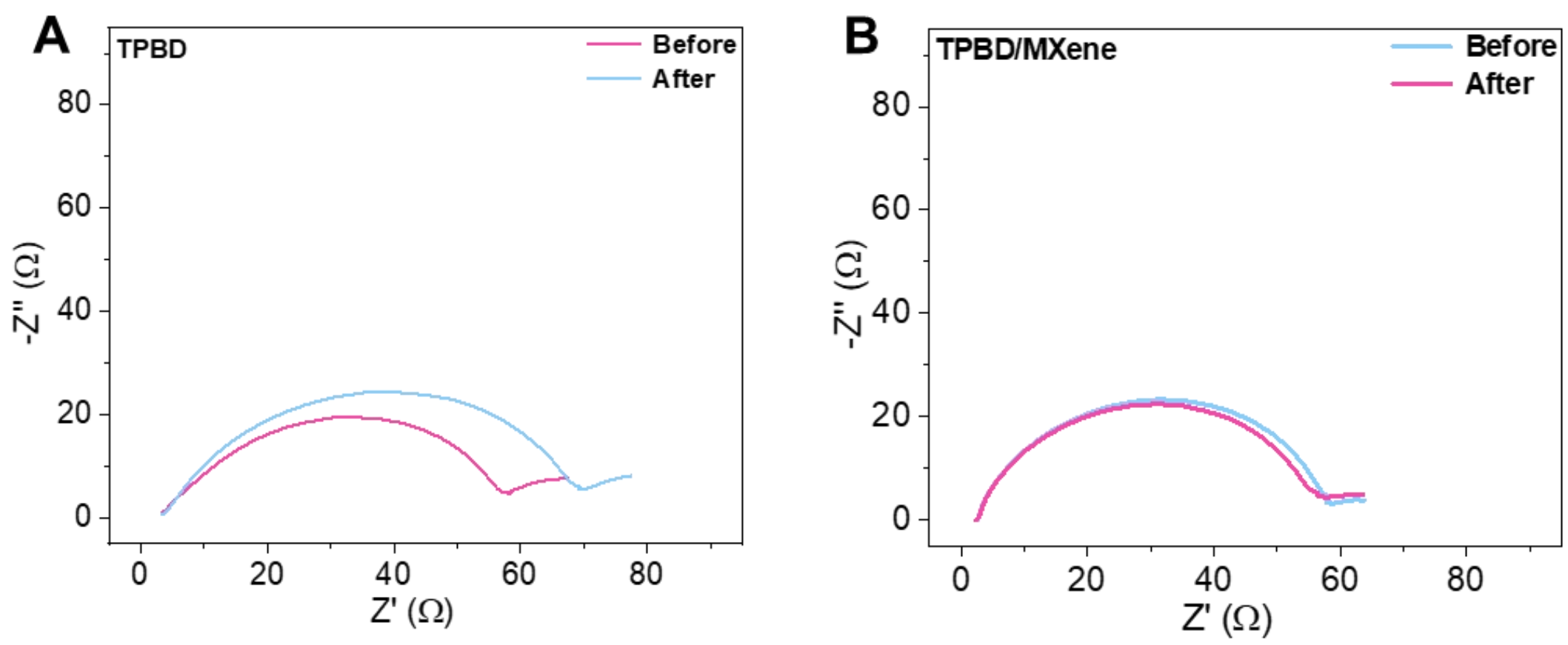

Fig. S16. EIS comparison of symmetric cells with different separators. EIS spectra of steel symmetric cells assembled with **(A)** TPBD and **(B)** TPBD/MXene modified separators before and after polarization.

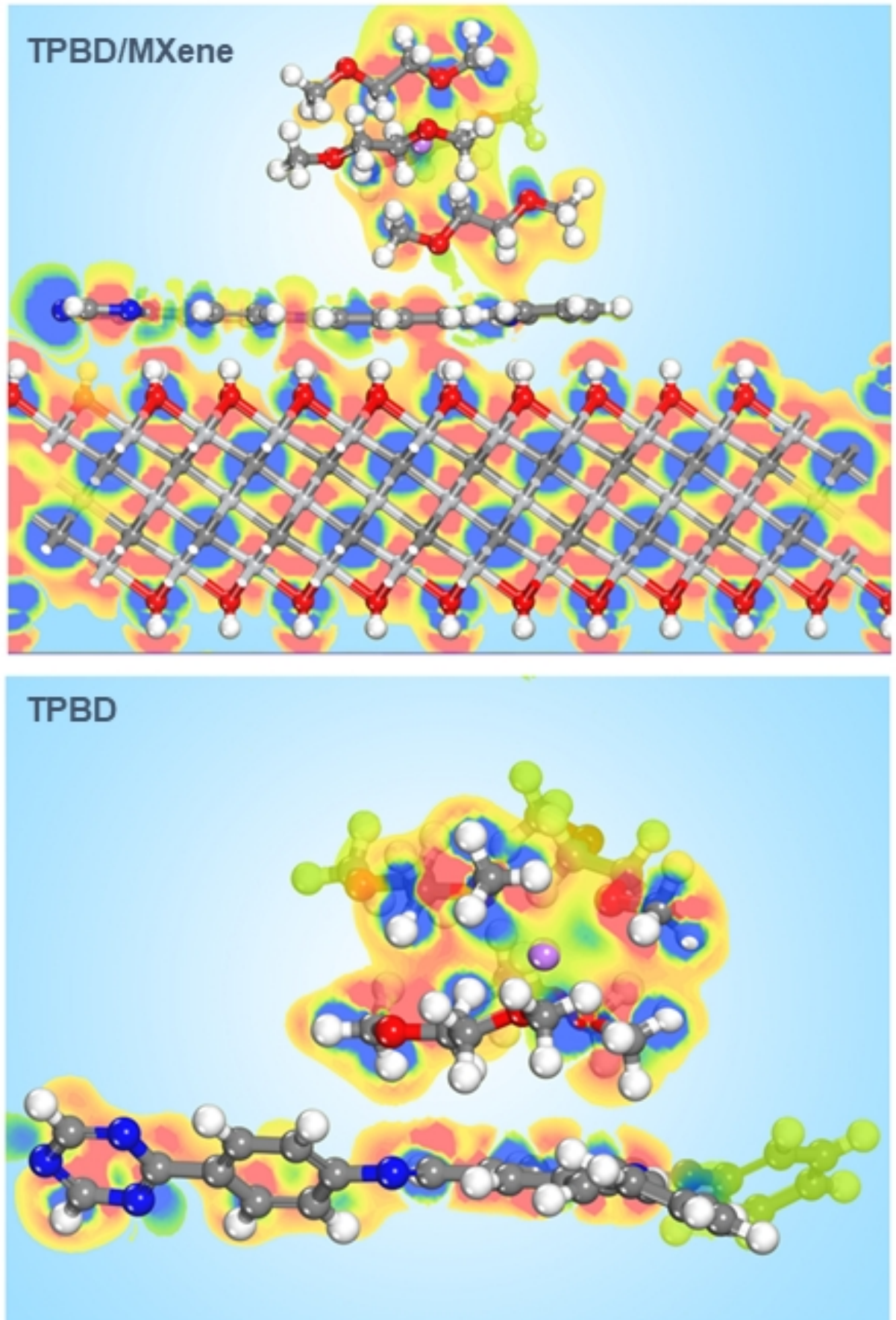

Fig. S17. Charge density difference maps of Li(DME)$_4^+$ on two substrates. Top-view differential charge density maps of Li(DME)$_4^+$ adsorbed on TPBD/MXene and TPBD.

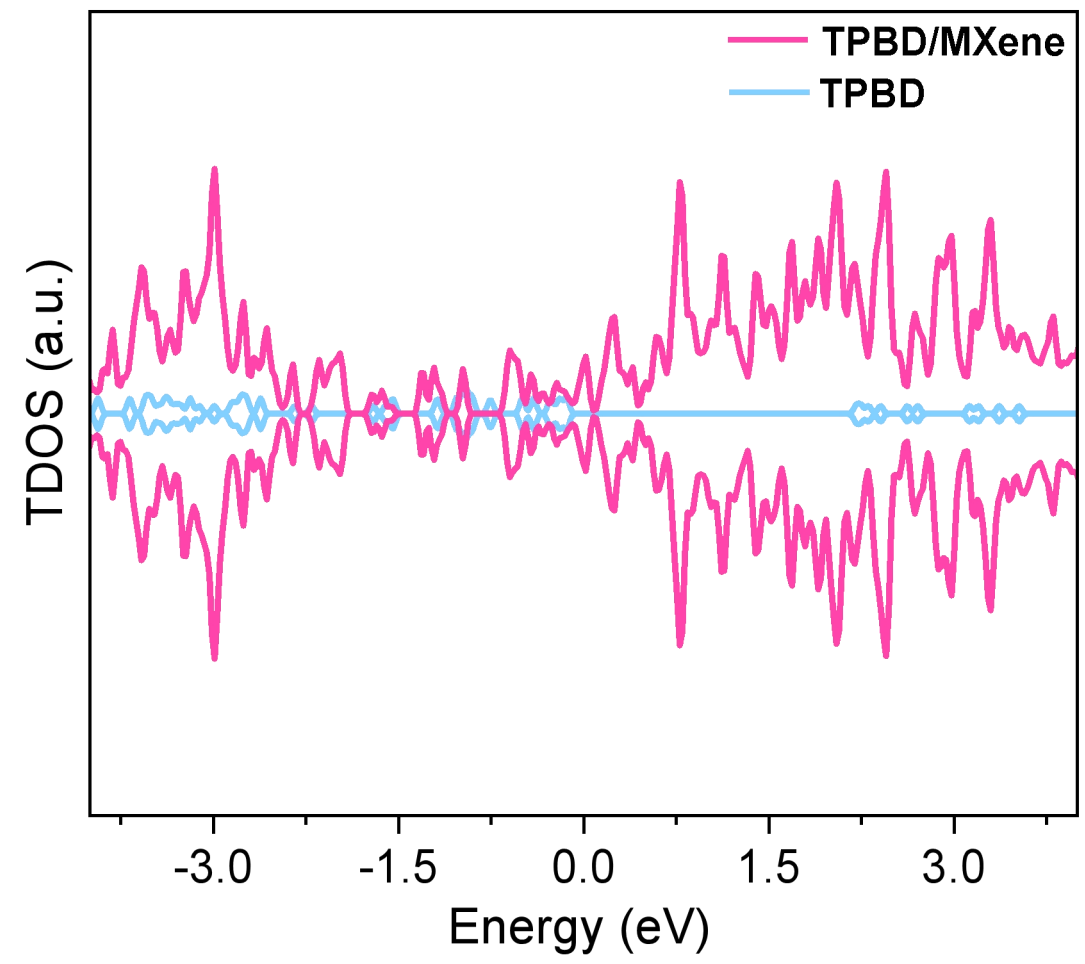

Fig. S18. Electronic structure. DOS of TPBD/MXene and TPBD.

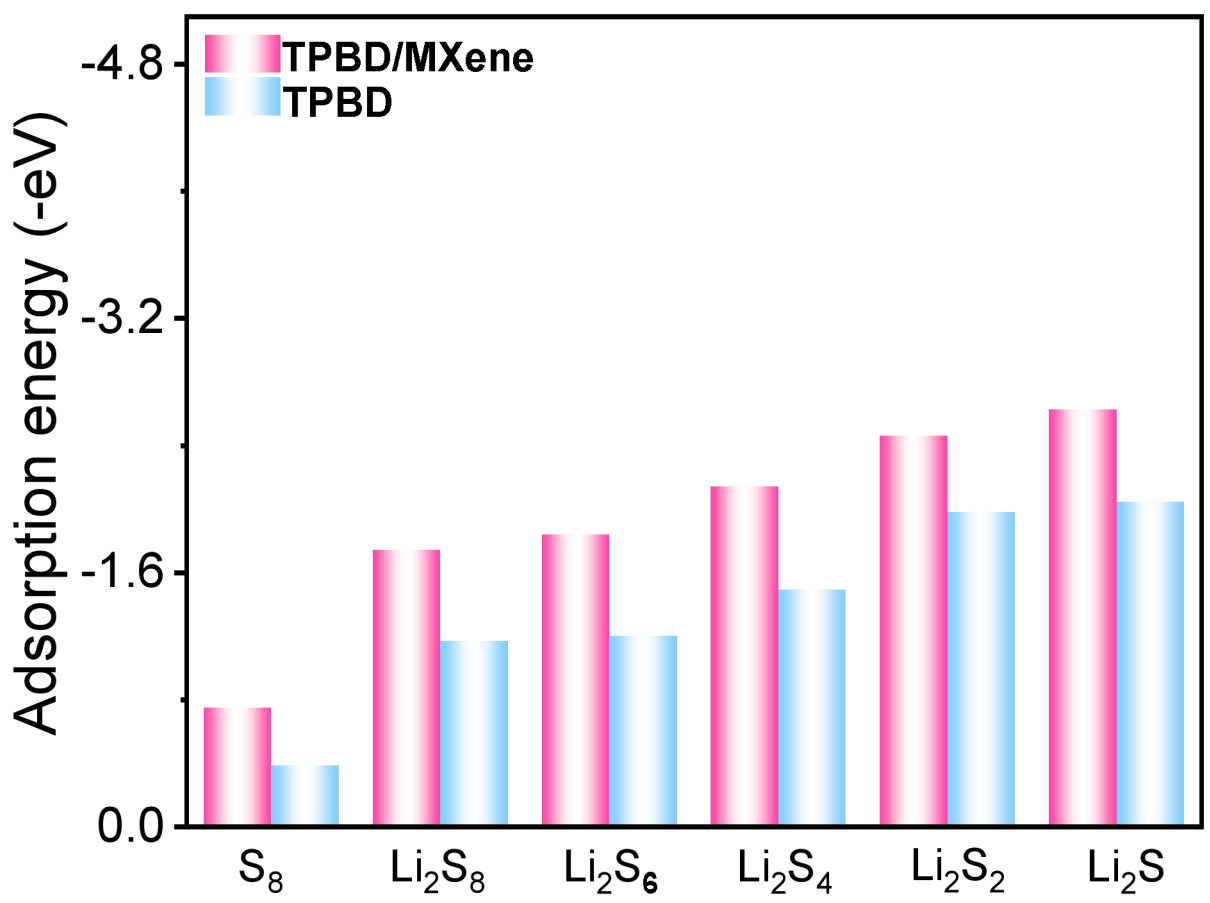

Fig. S19. Interaction energy between substrate and sulfur species. Adsorption energy calculation for diverse sulfur species (S_8, Li_2S_8, Li_2S_6, Li_2S_4, Li_2S_2, and Li_2S).

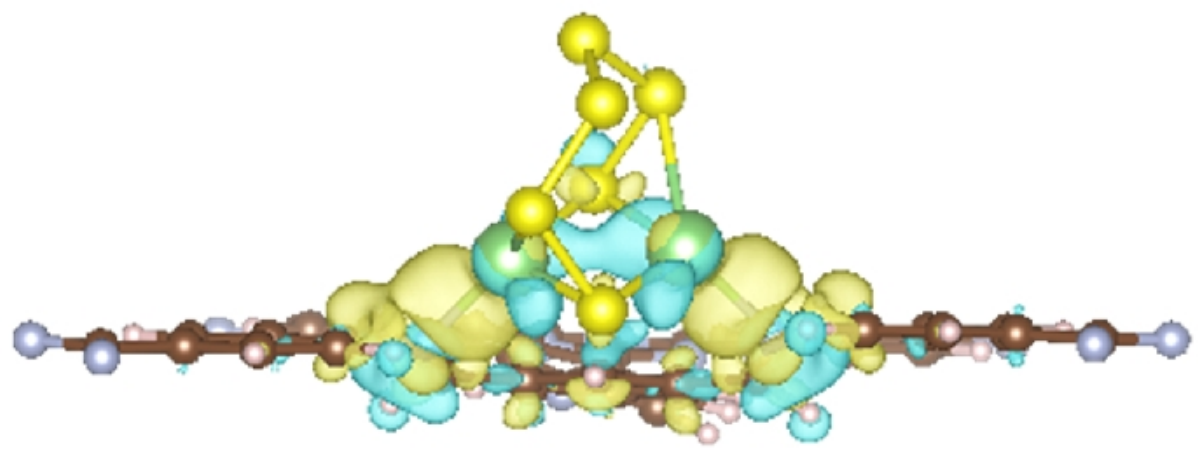

Fig. S20. Charge redistribution. Charge difference after binding to Li_2S_6 on the TPBD surface.

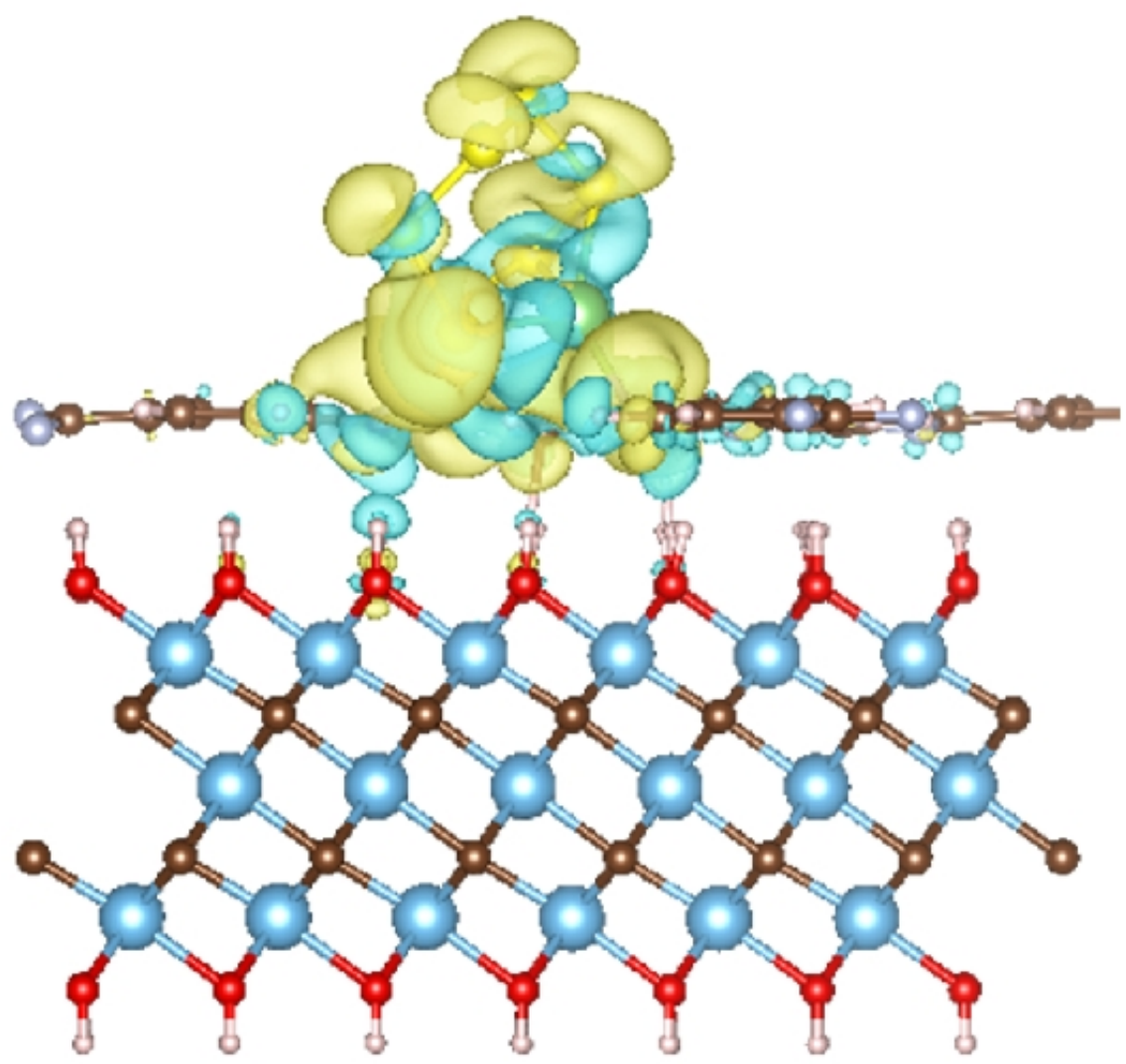

Fig. S21. Differential charge density. Charge difference after binding to Li_2S_6 on the TPBD/MXene surface.

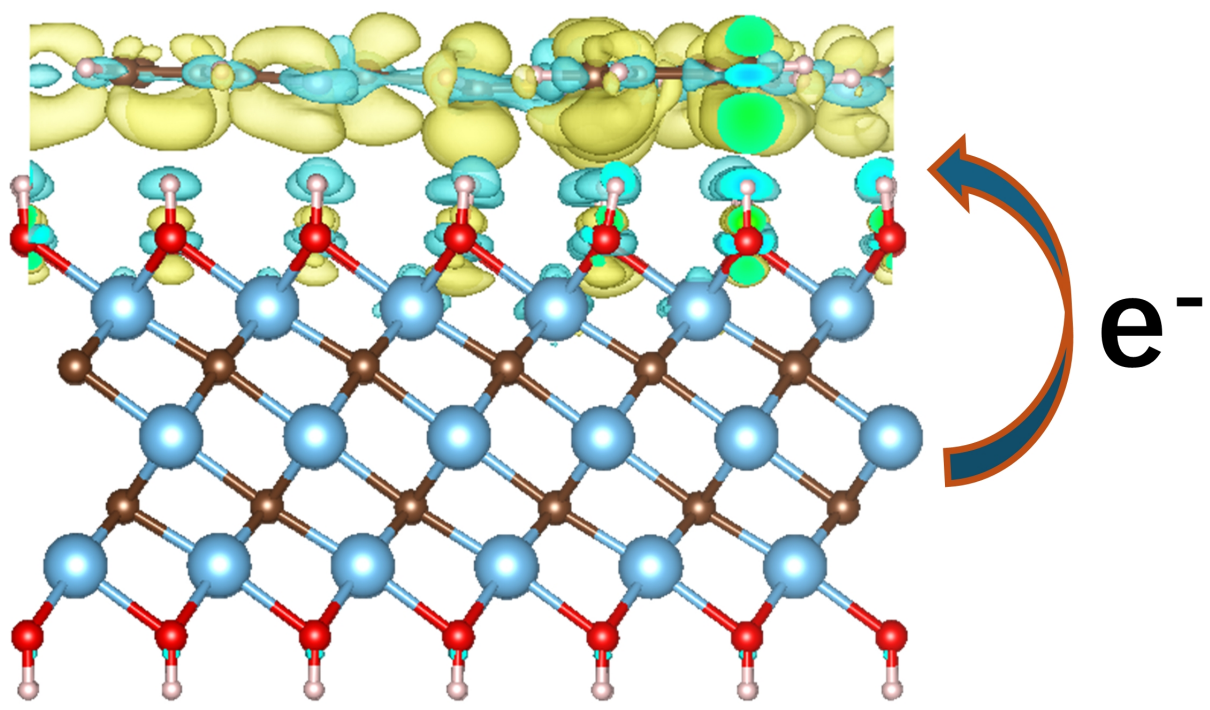

Fig. S22. Charge density difference at the TPBD/MXene interface. Differential charge density diagram illustrating the interaction between TPBD and MXene.

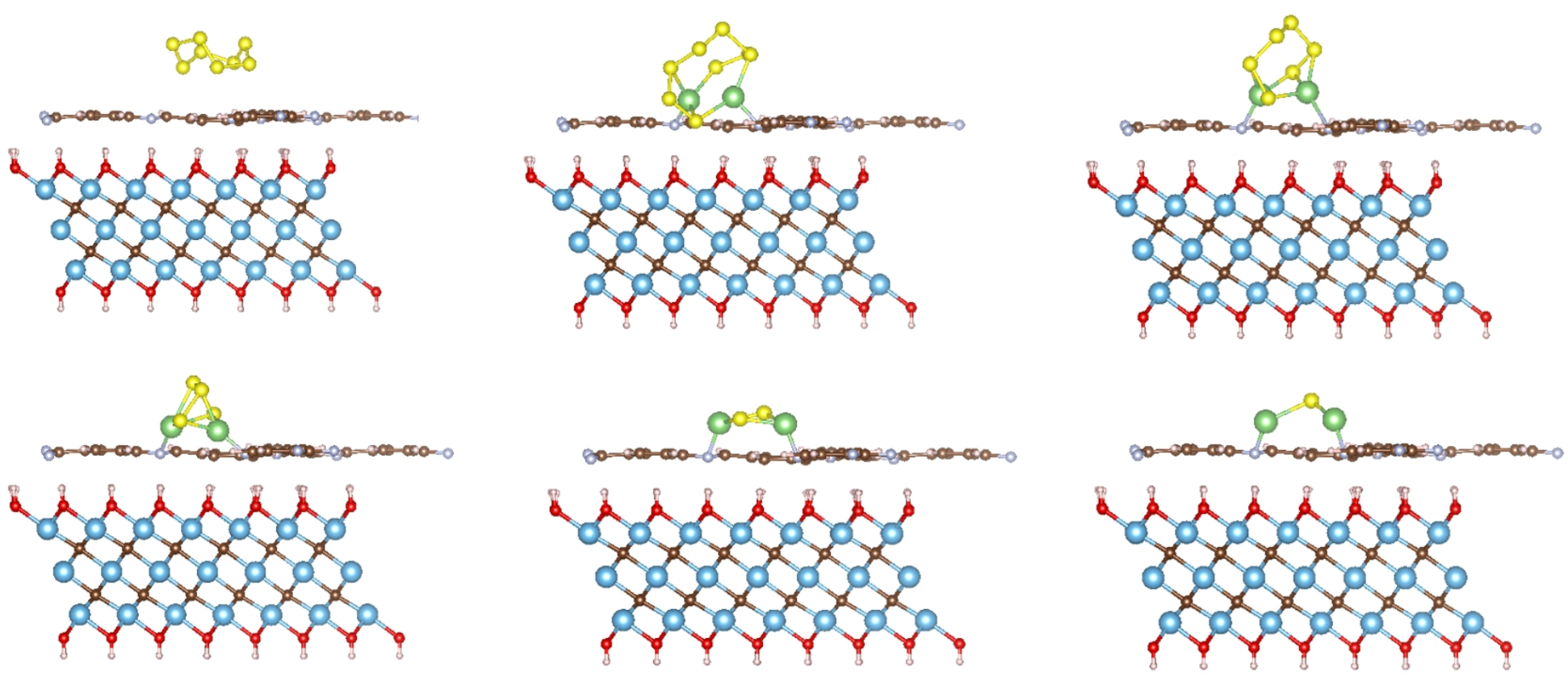

Fig. S23. Optimized geometries. Optimized geometries of LiPSs (Li_2S_8, Li_2S_6, Li_2S_4, Li_2S_2, Li_2S) and S_8 adsorbed on the TPBD/MXene surface from DFT calculations.

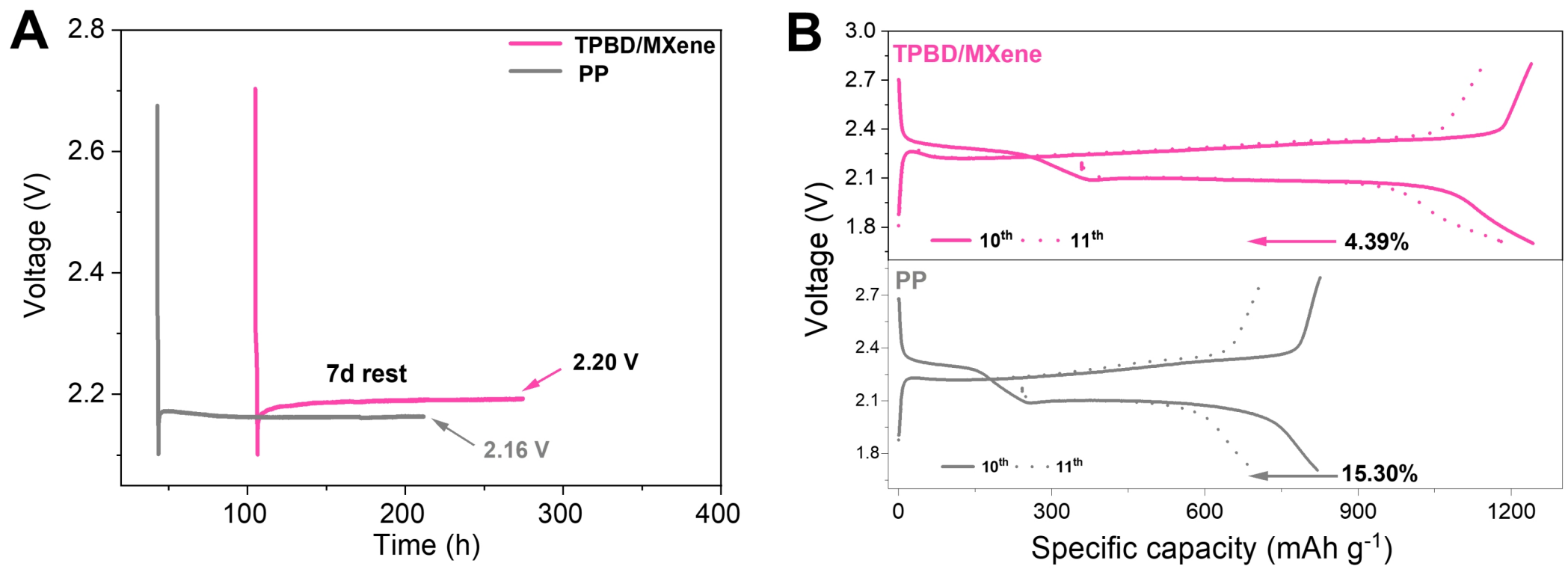

Fig. S24. Self-discharge behavior and capacity retention comparison. (A) Self-discharge tests, **(B)** A comparative analysis of the capacity retention rates after standing for TPBD/MXene and PP.

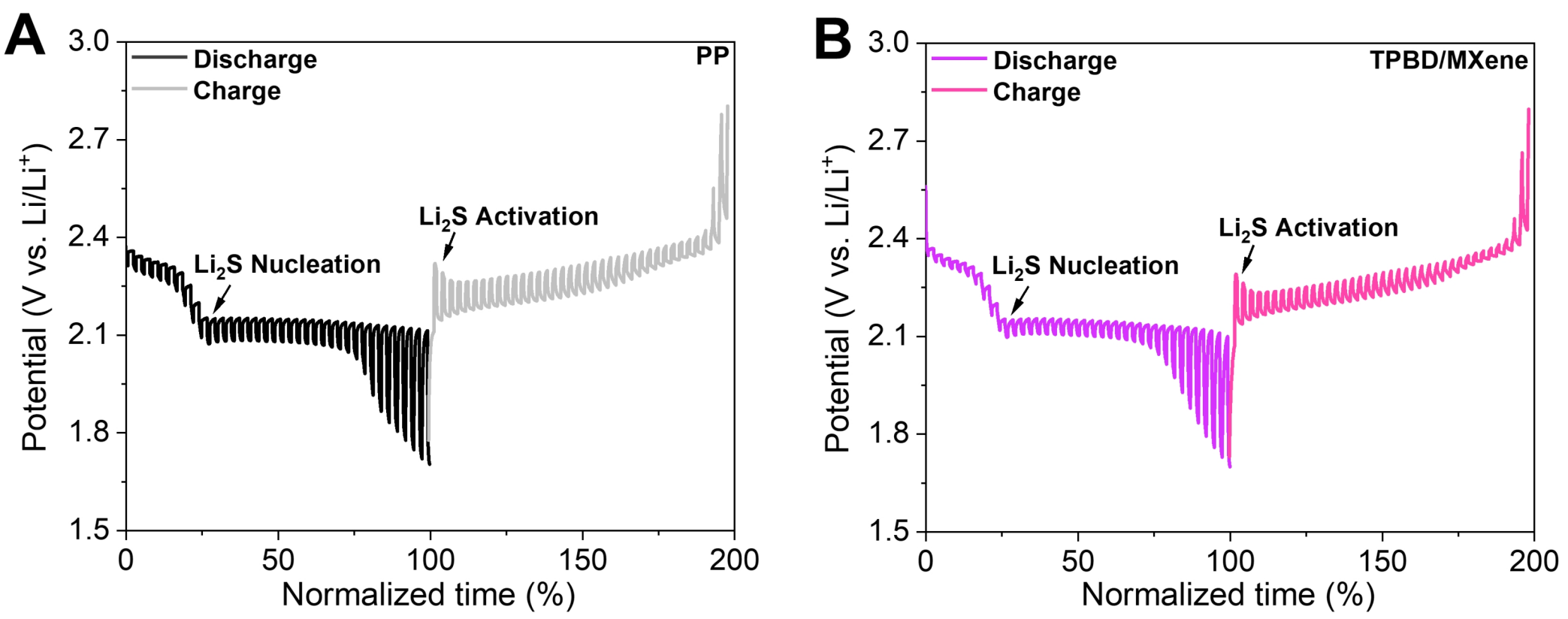

Fig. S25. GITT profiles. GITT curves for **(A)** PP and **(B)** TPBD/MXene separators at 0.1 C.

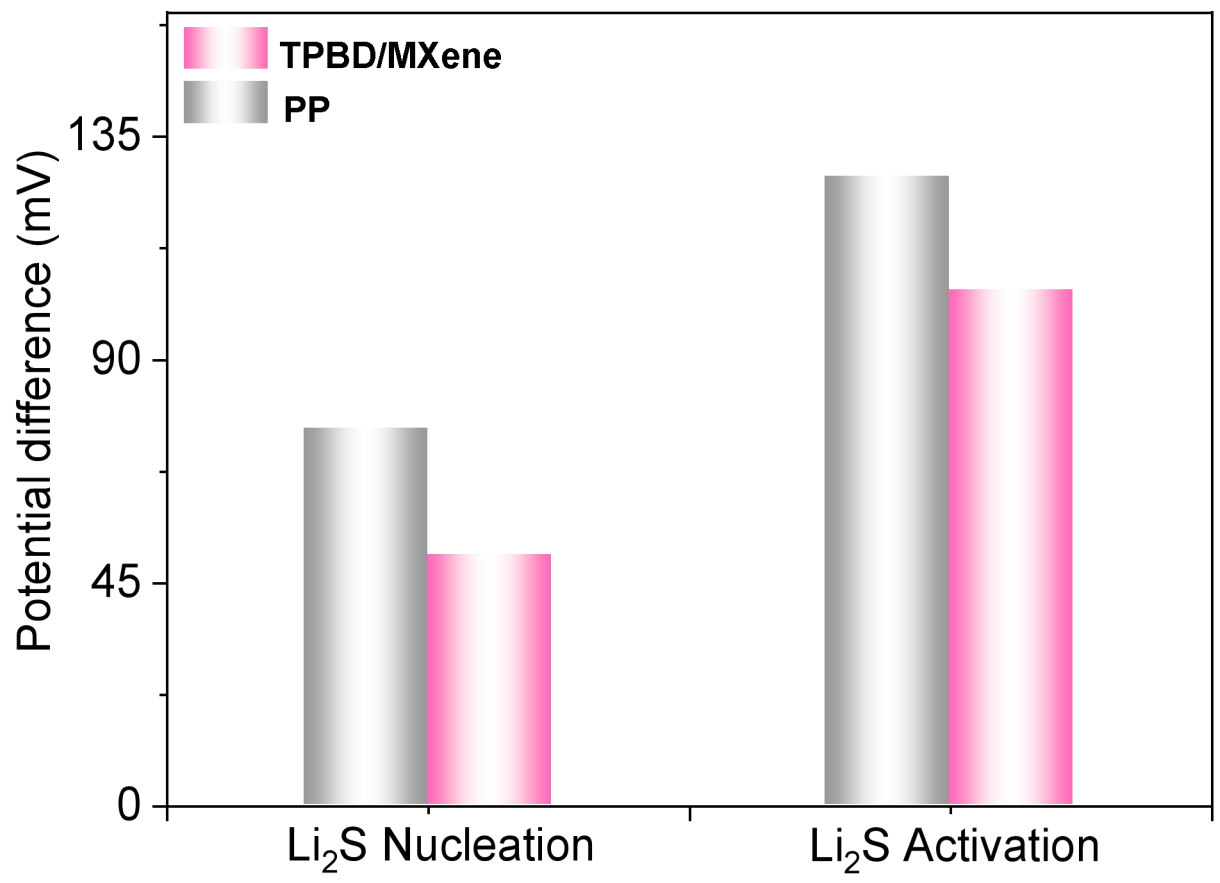

Fig. S26. Voltage gap at Li_2S activation and nucleation sites. The voltage difference between QOCV and CCV at Li_2S activation site and Li_2S nucleation site.

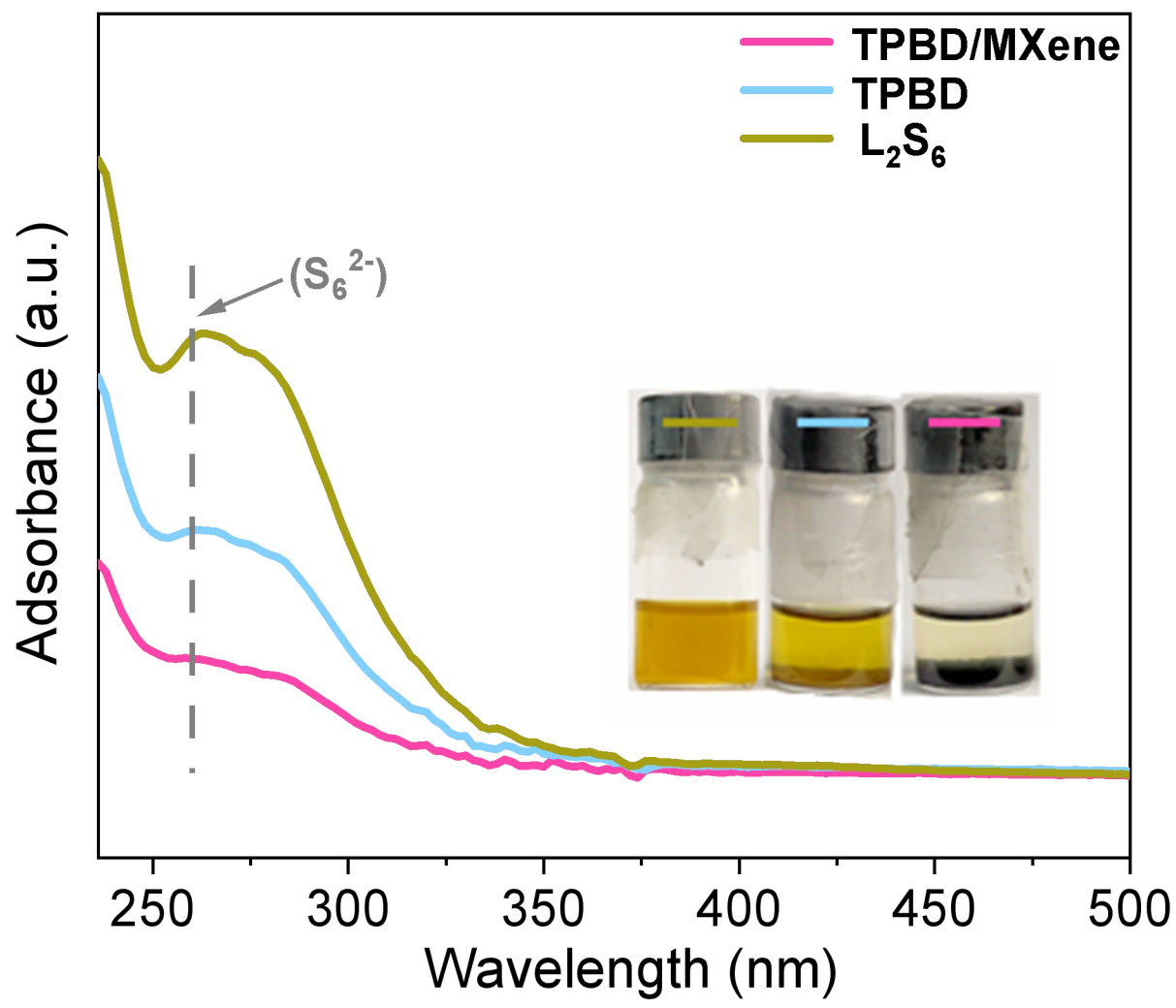

Fig. S27. Polysulfide adsorption monitored by UV-vis and digital imaging. UV-vis spectroscopic curves of TPBD/MXene and TPBD after adsorption of polysulfide (Inset: digital photos of adsorption experiments).

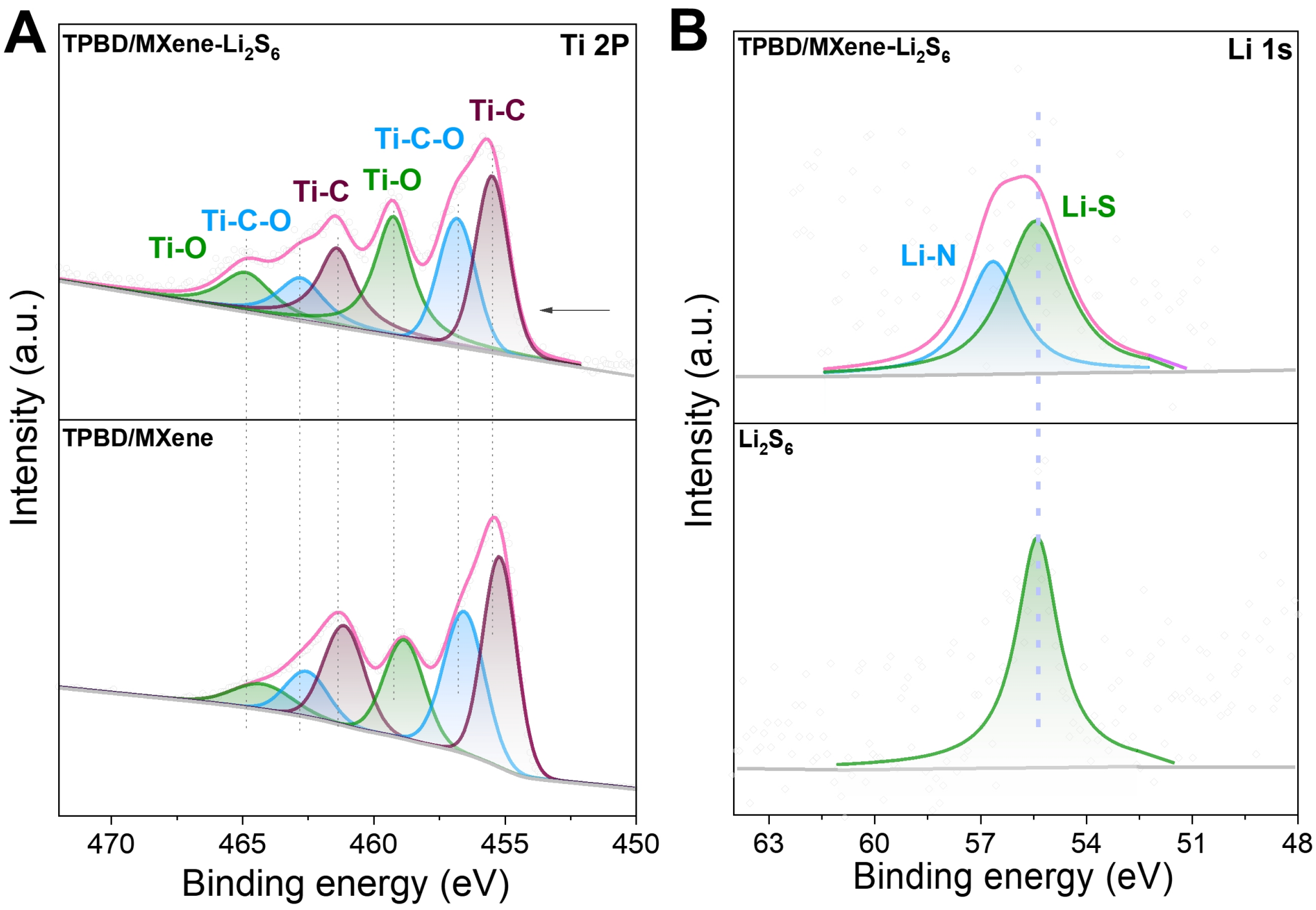

Fig. S28. XPS analysis. XPS spectra of **(A)** Ti 2p and **(B)** Li 1s of TPBD/MXene before and after polysulfide adsorption.

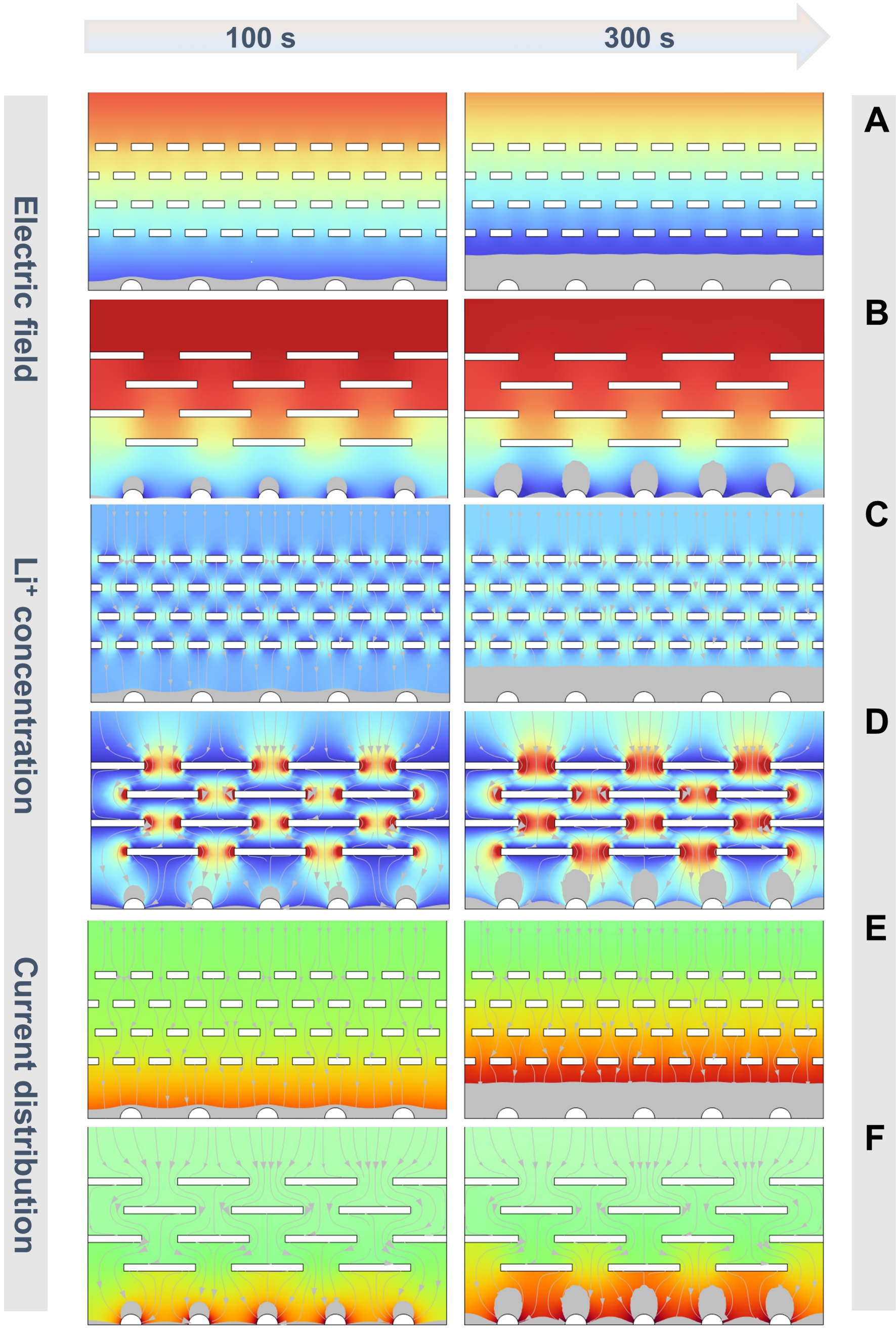

Fig. S29. COMSOL simulation of modified separator for suppressing lithium dendrites. electric field distribution, Li^+ concentration, and current distribution with **(A, C, E)** TPBD/MXene separator and **(B, D, F)** TPBD separator.

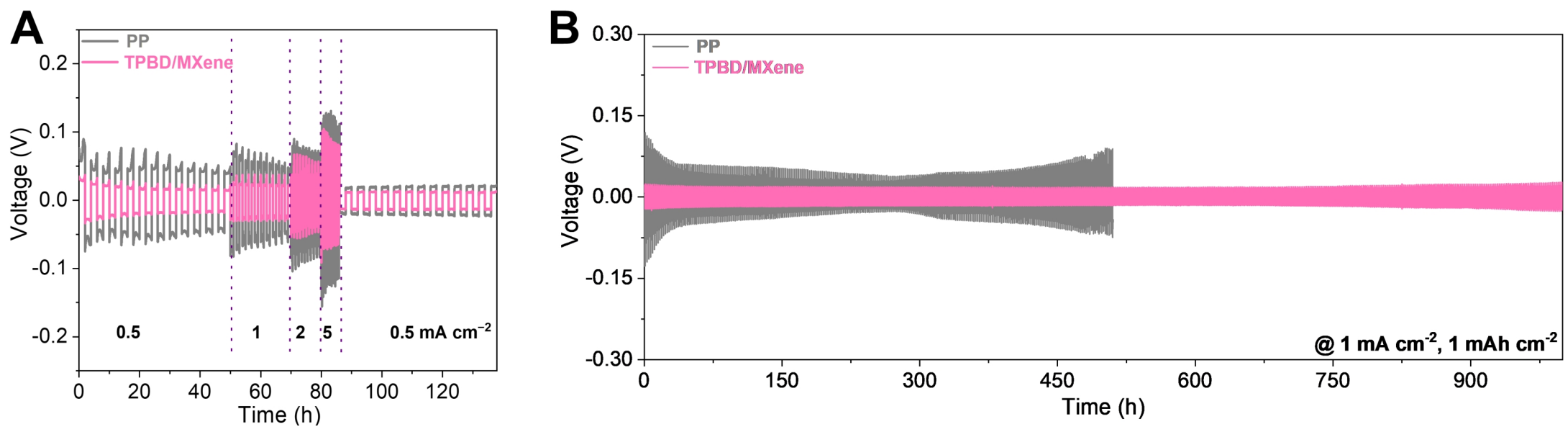

Fig. S30. Rate performance and cycling stability of Li//Li symmetric cells. (A) Rate capability and **(B)** cyclability of TPBD/MXene and PP separators-based Li//Li symmetric batteries.

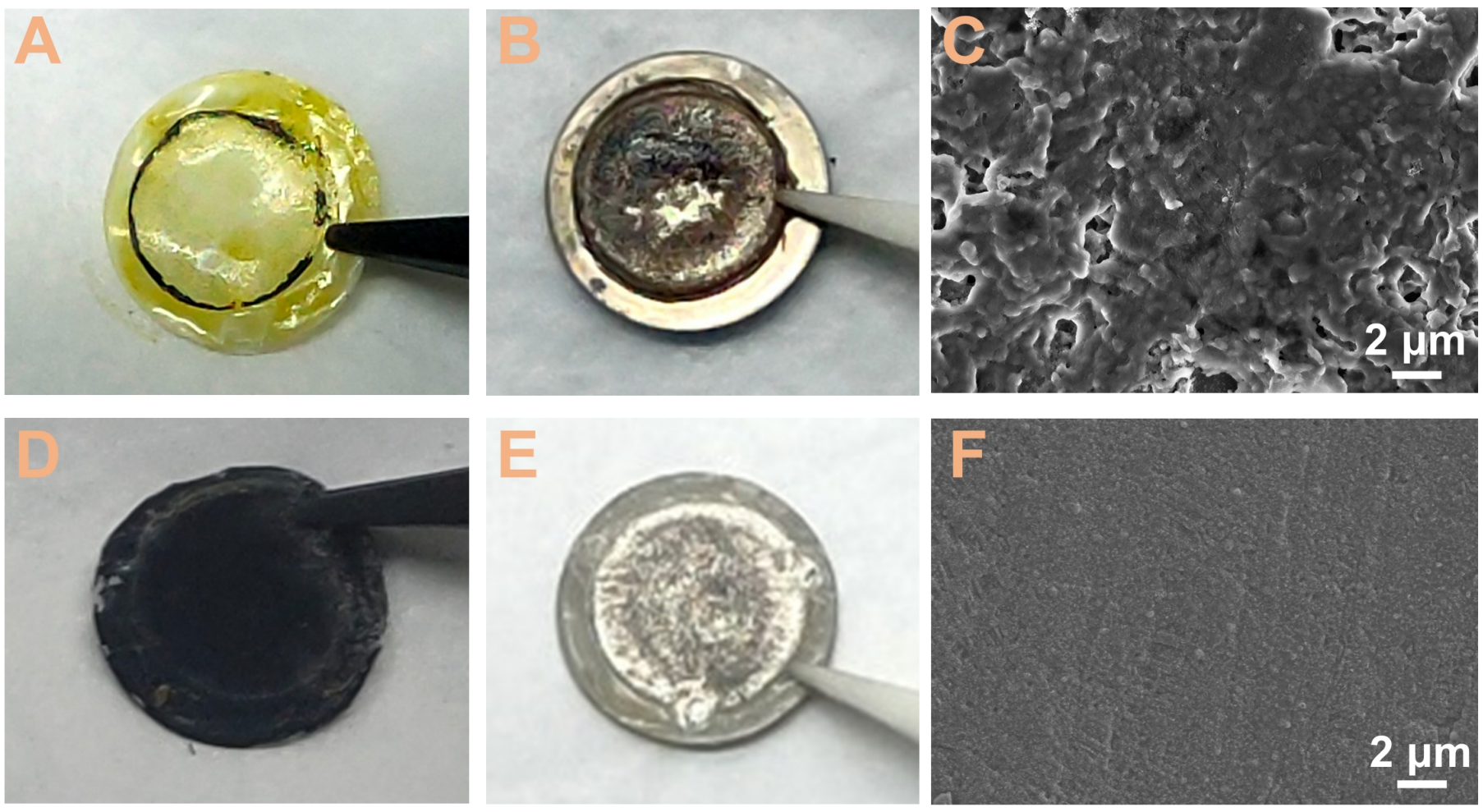

Fig. S31. Morphology of separator and lithium anode after cycling. Photographs of the separators and lithium anode of **(A, B)** PP and **(D, E)** TPBD/MXene. SEM photographs of the lithium anode after 100 cycles with **(C)** PP and **(F)** TPBD/MXene separators.

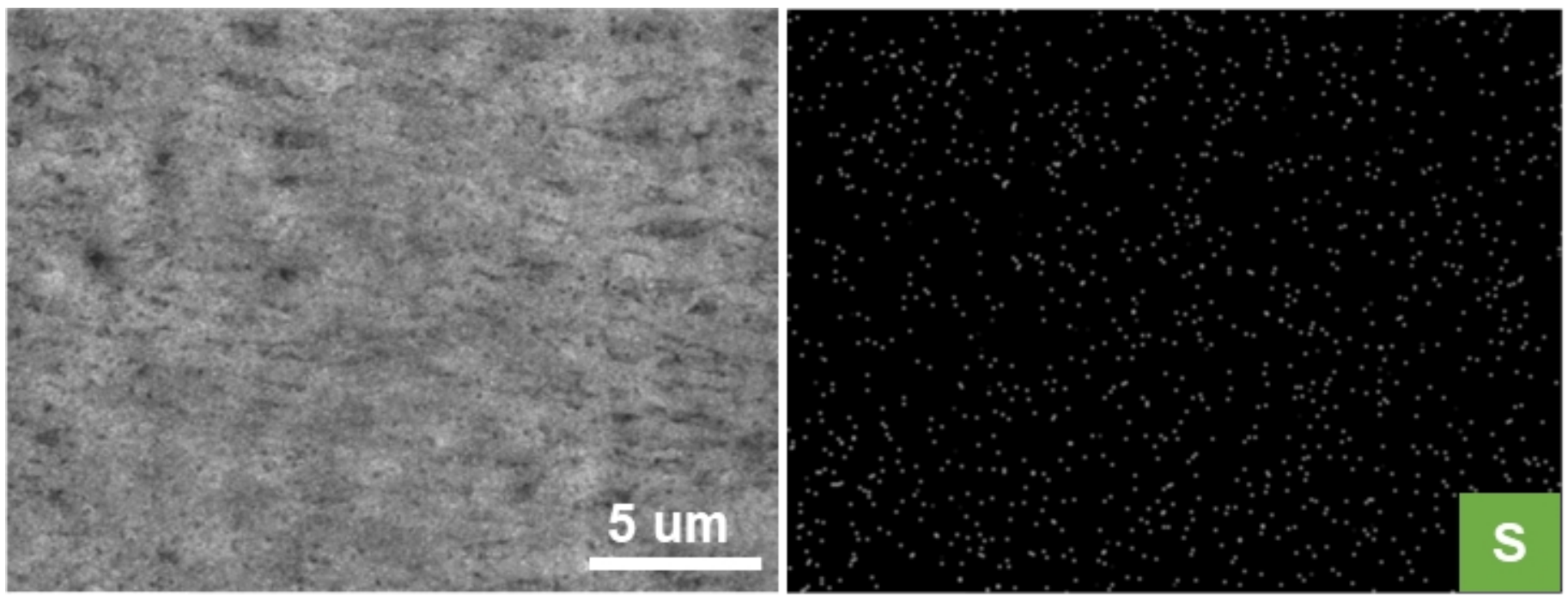

Fig. S32. Spatial distribution of sulfur on cycled lithium anode. Sulfur mapping image of the lithium anode paired with the TPBD/MXene modified separator.

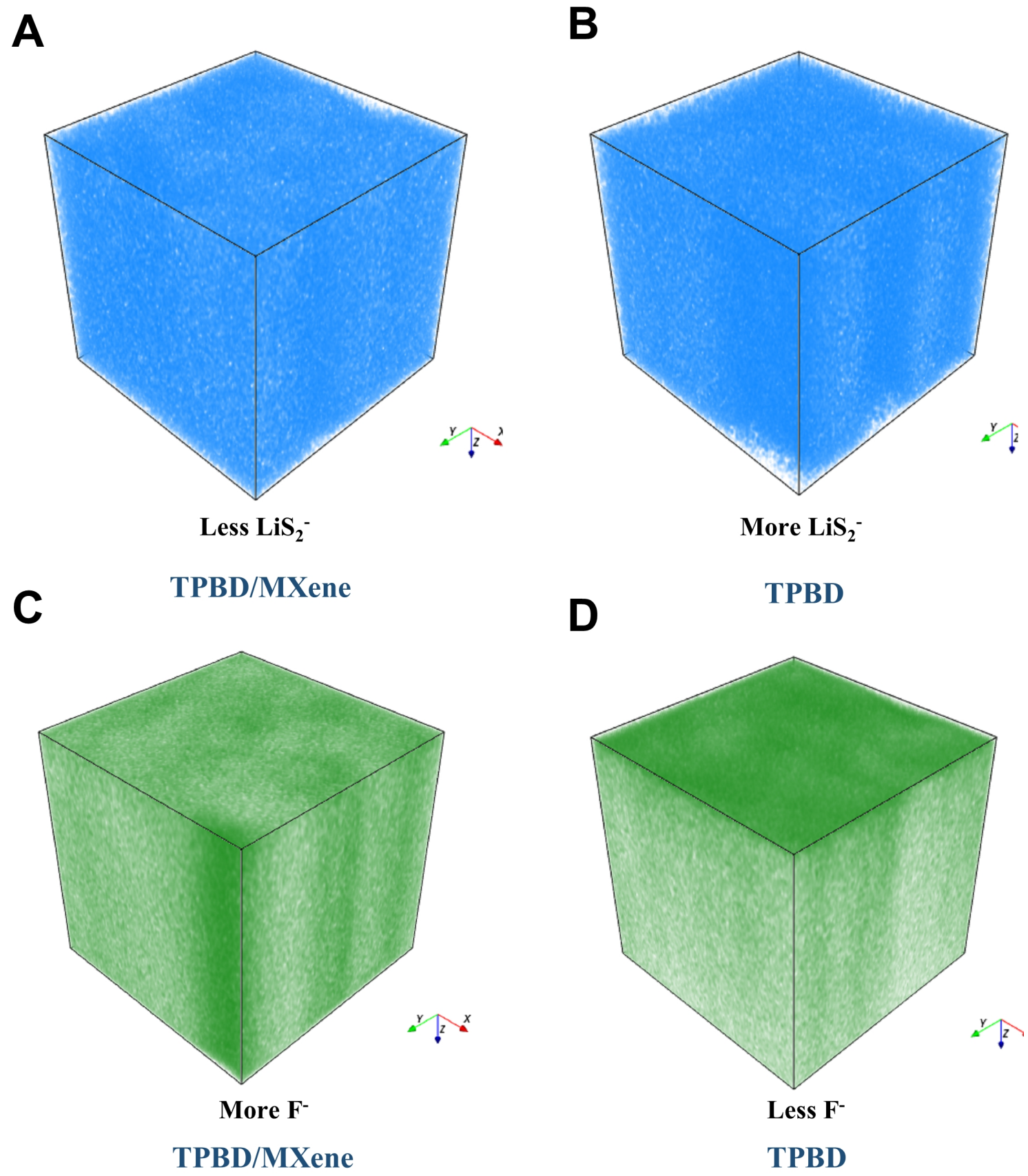

Fig. S33. 3D distribution of LiS_2^- and F^- species. 3D rendering reconstruction of **(A, B)** LiS_2^- and **(C, D)** F^- species with TPBD/MXene and TPBD systems.

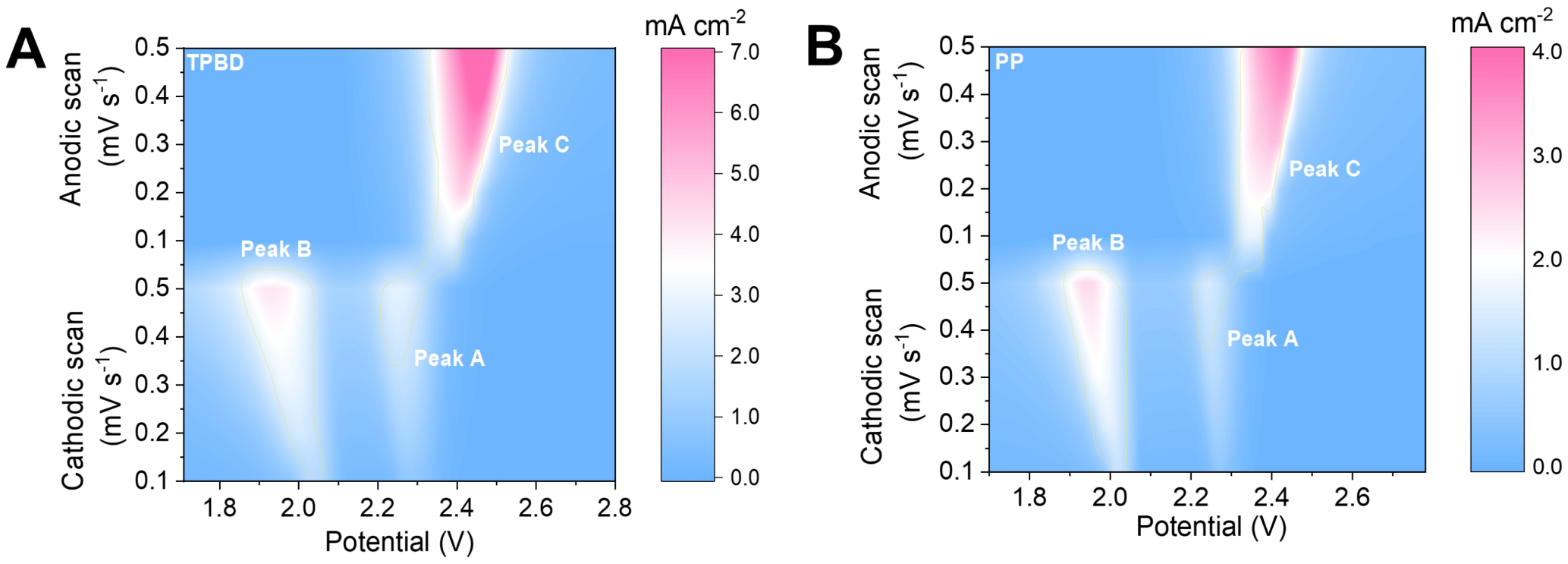

Fig. S34. CV contour maps of batteries with TPBD and PP separators. Contour plots of CV curves at different scan rates for batteries with **(A)** TPBD and **(B)** PP separators.

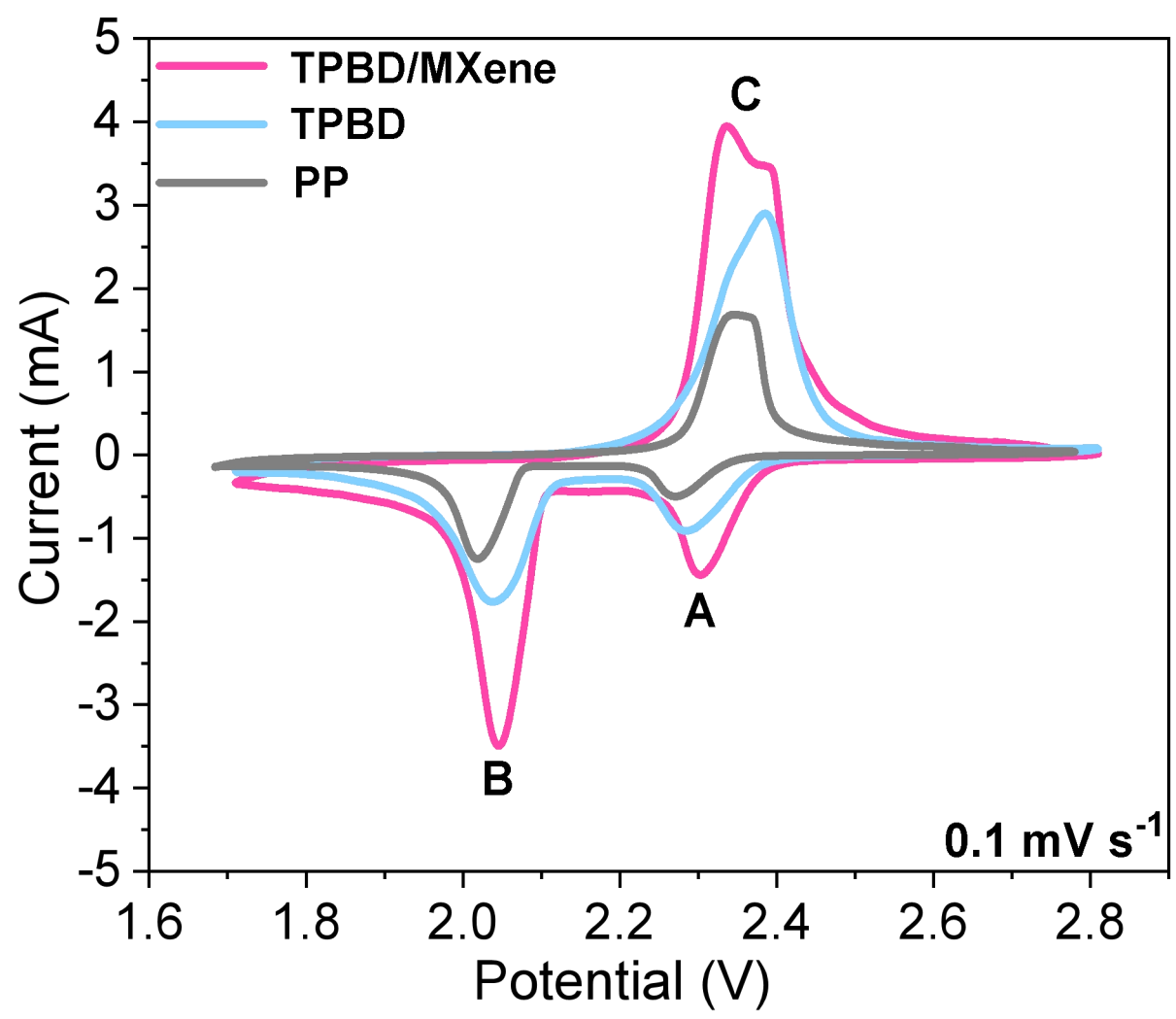

Fig. S35. CV comparison of three separators. The CV profiles of TPBD/MXene heterostructure, TPBD, and PP at a scan rate of 0.1 mV s^{-1}.

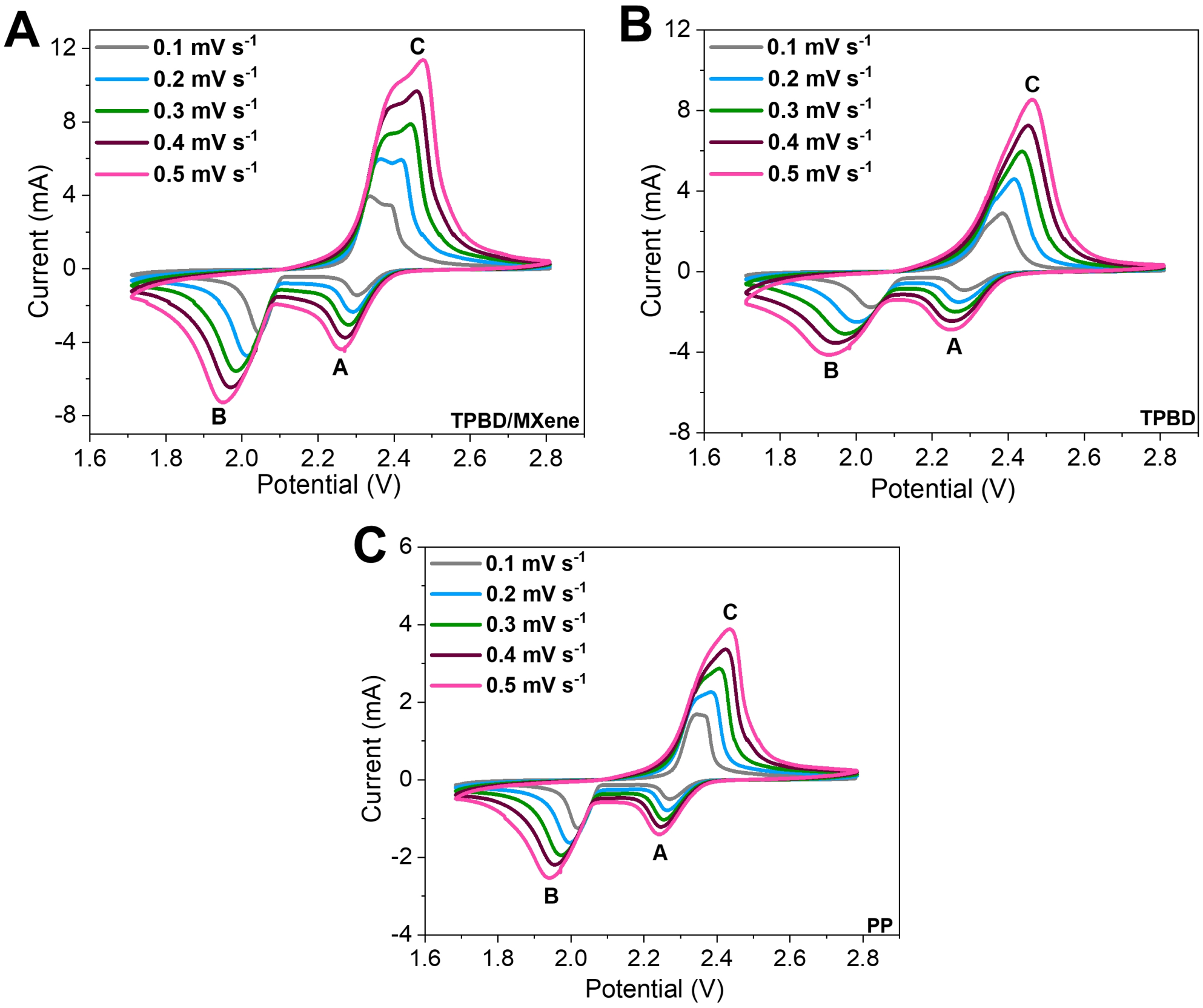

Fig. S36. Comparative CV of three separators at varying scan rates. The CV spectra of **(A)** TPBD/MXene heterostructure, **(B)** TPBD, and **(C)** PP with scan rates ranging from 0.1 to 0.5 mV s^{-1}.

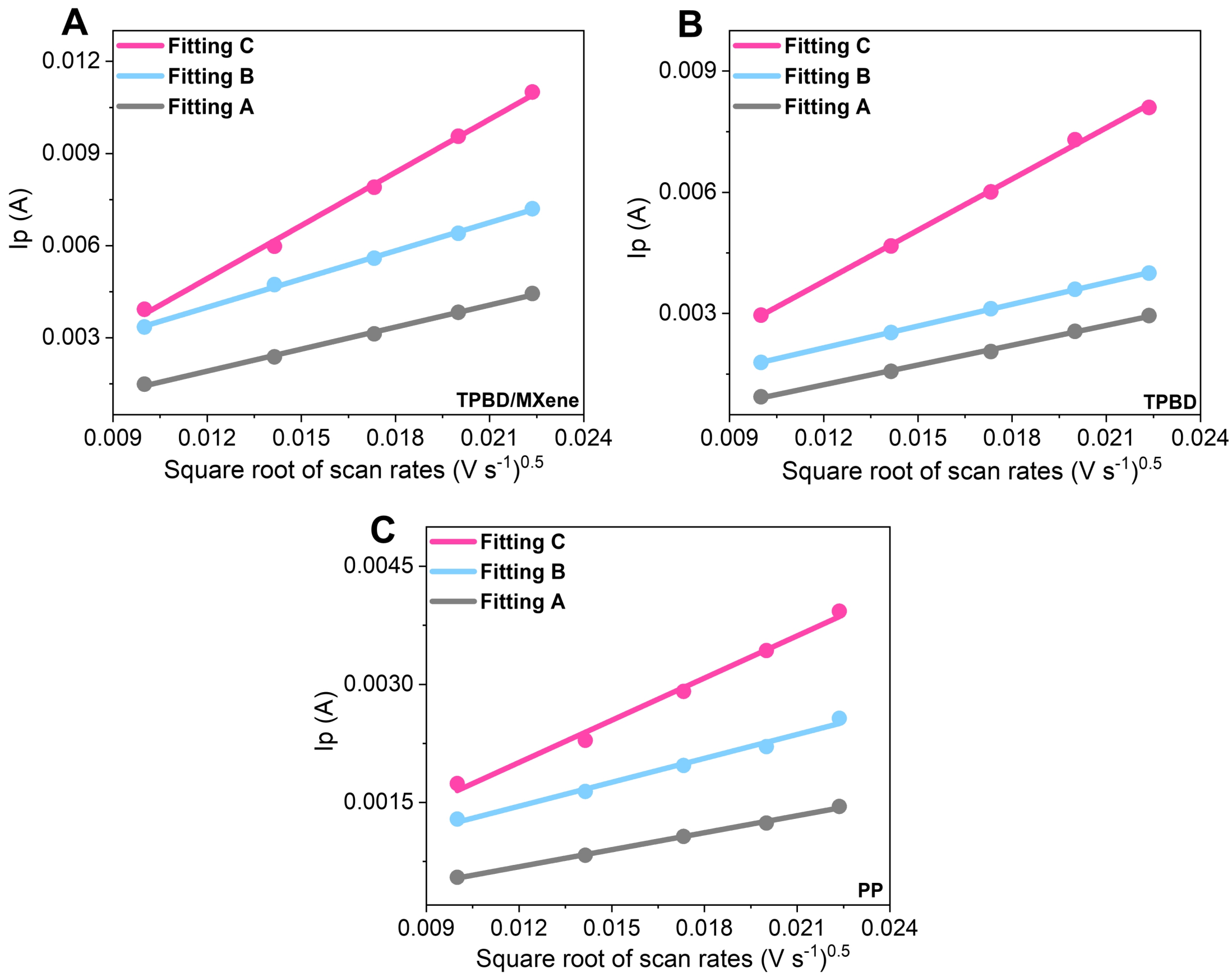

Fig. S37. Linear fitting of peak currents to scan rate square roots. The fitted profiles of peak currents versus square root of scan rates with the batteries of **(A)** TPBD/MXene, **(B)** TPBD, and **(C)** PP separators.

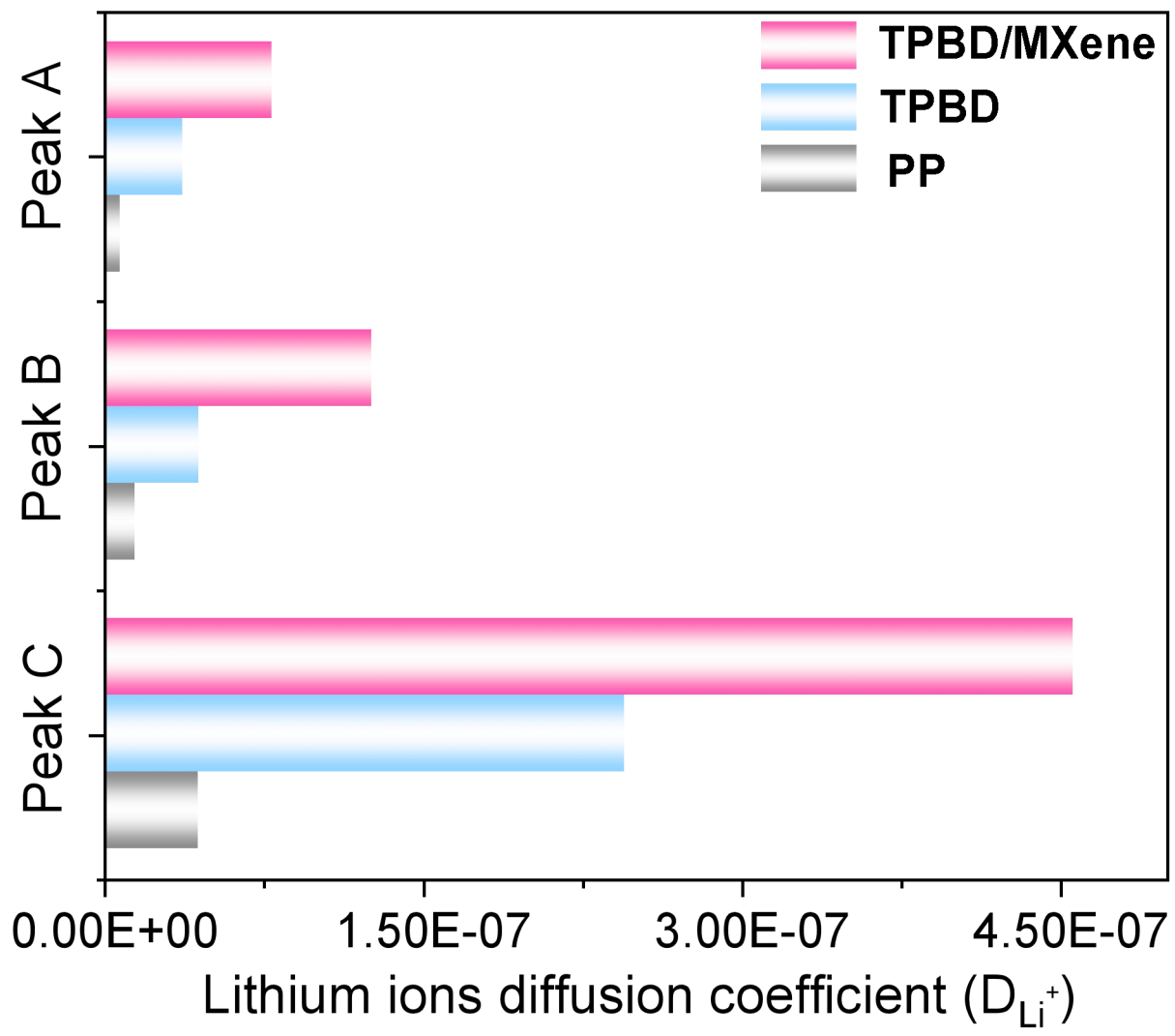

Fig. S38. lithium ions diffusion coefficients of peaks A, B, and C. The diffusion coefficients of lithium ions for C, A, and B peaks.

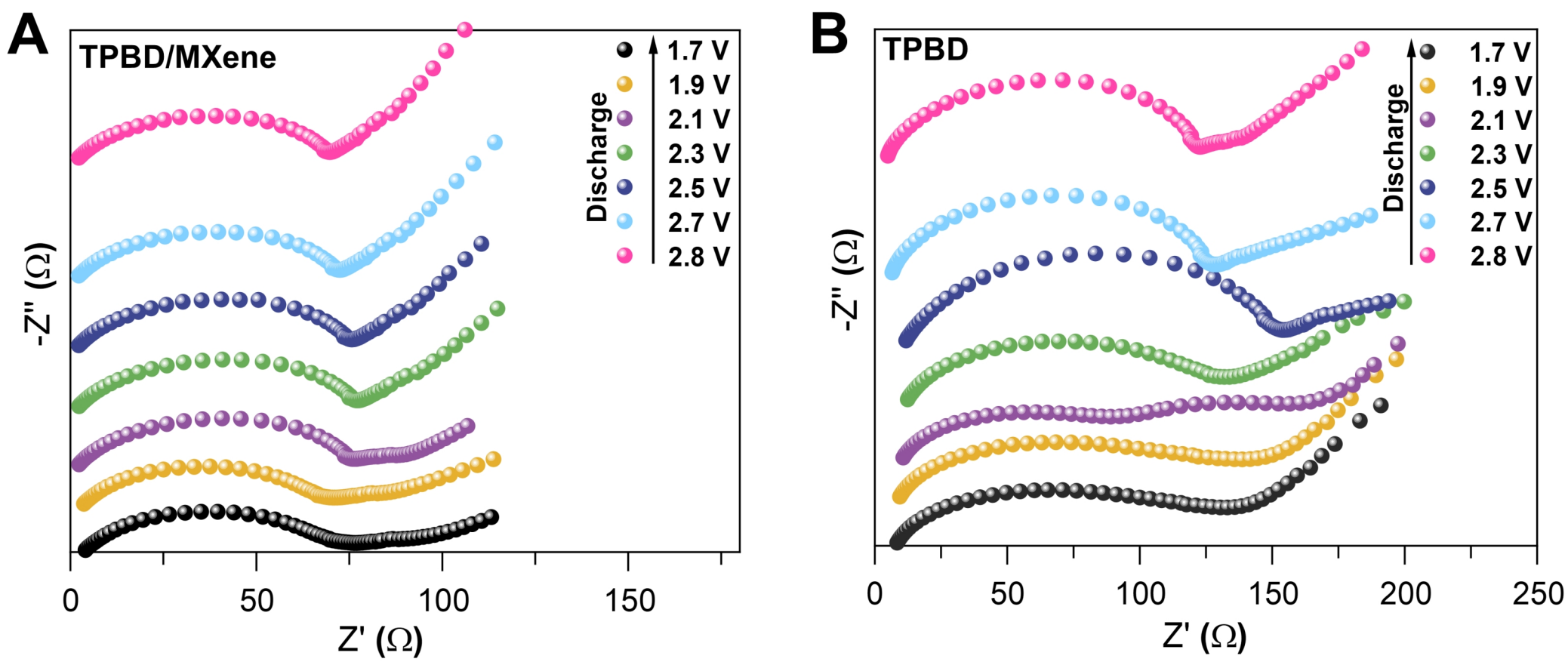

Fig. S39. Dynamic EIS analysis upon discharging. Operando EIS monitoring of impedance evolution during discharge for batteries employing **(A)** TPBD/MXene and **(B)** TPBD separators.

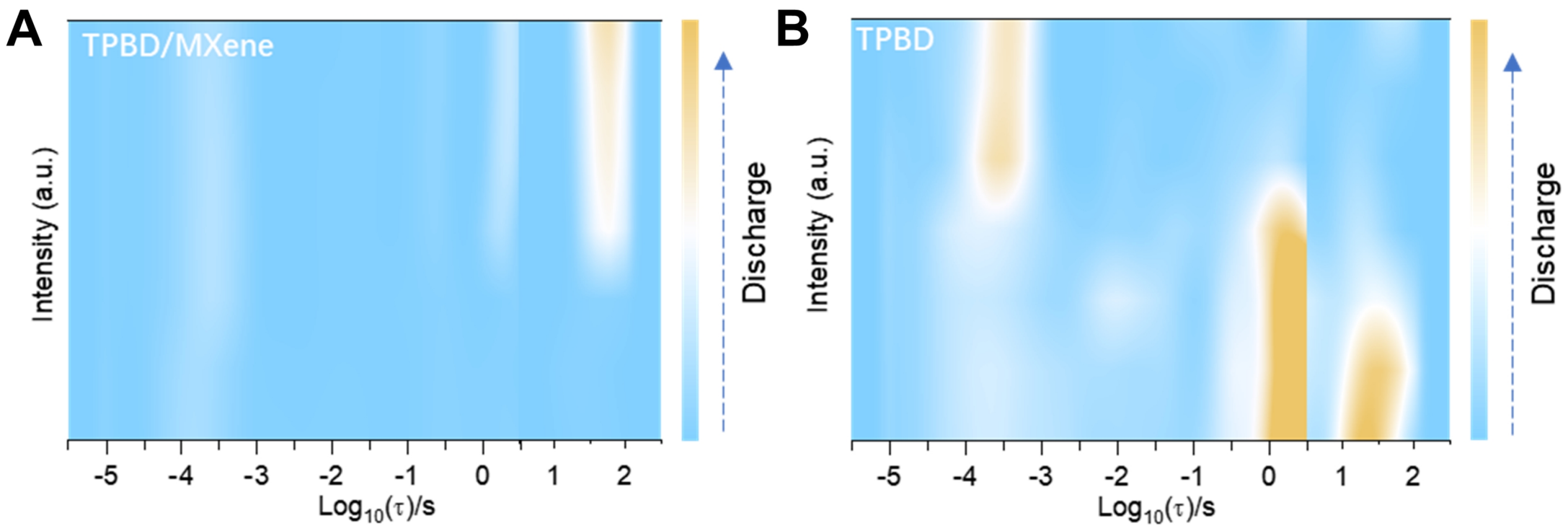

Fig. S40. DRT spectral intensity versus discharge rate.

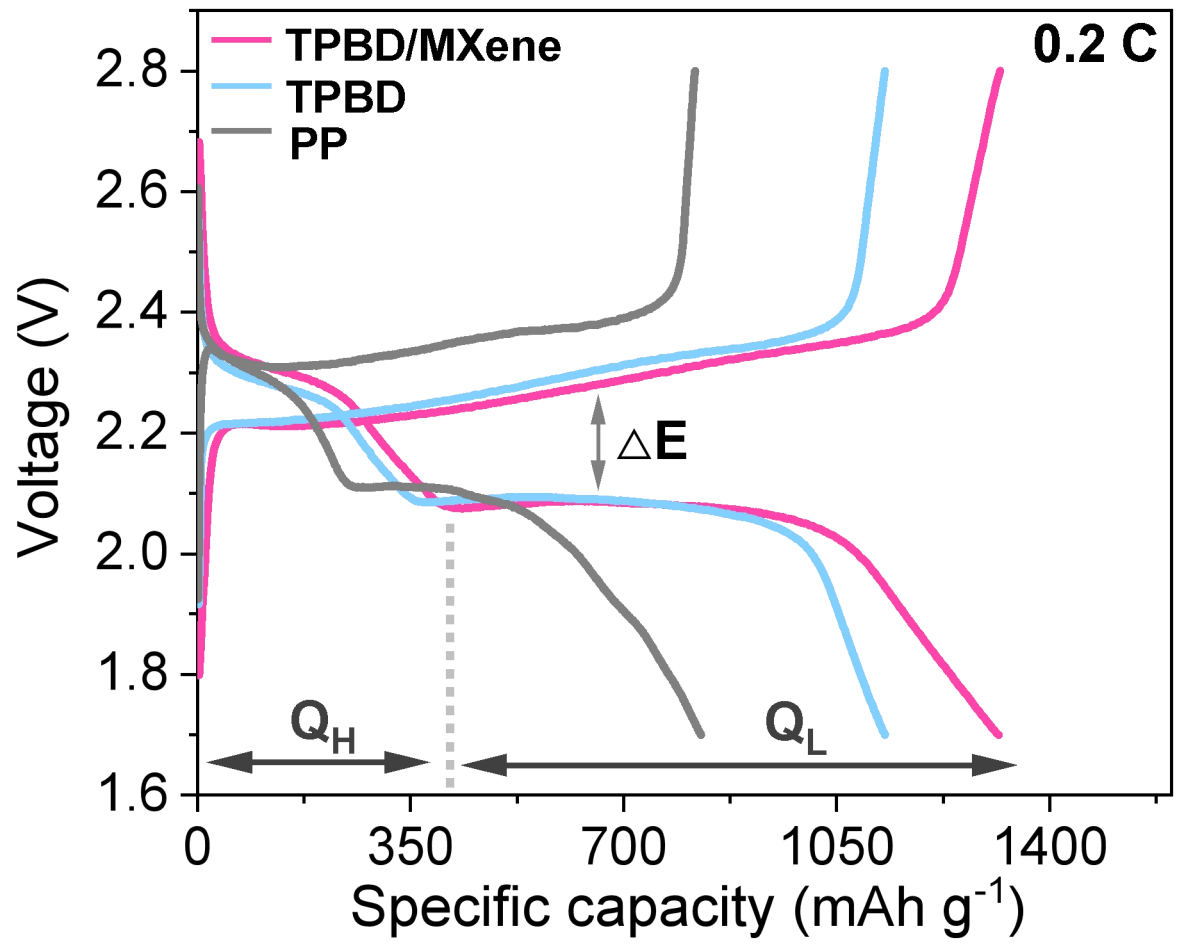

Fig. S41. Galvanostatic charge-discharge profiles at 0.2 C.

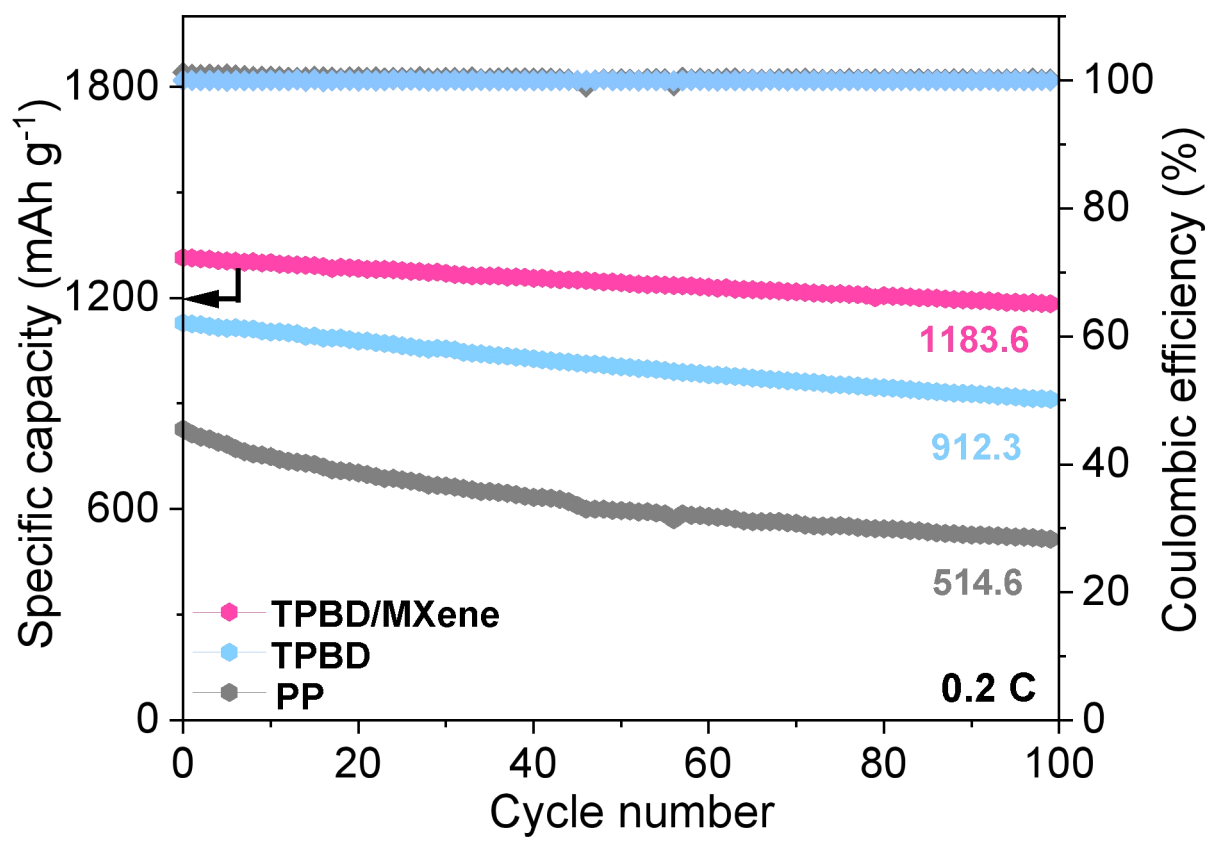

Fig. S42. Cycle performance of three separators at 0.2 C. Cycling performance of batteries with TPBD/MXene, TPBD, and PP separators at 0.2 C.

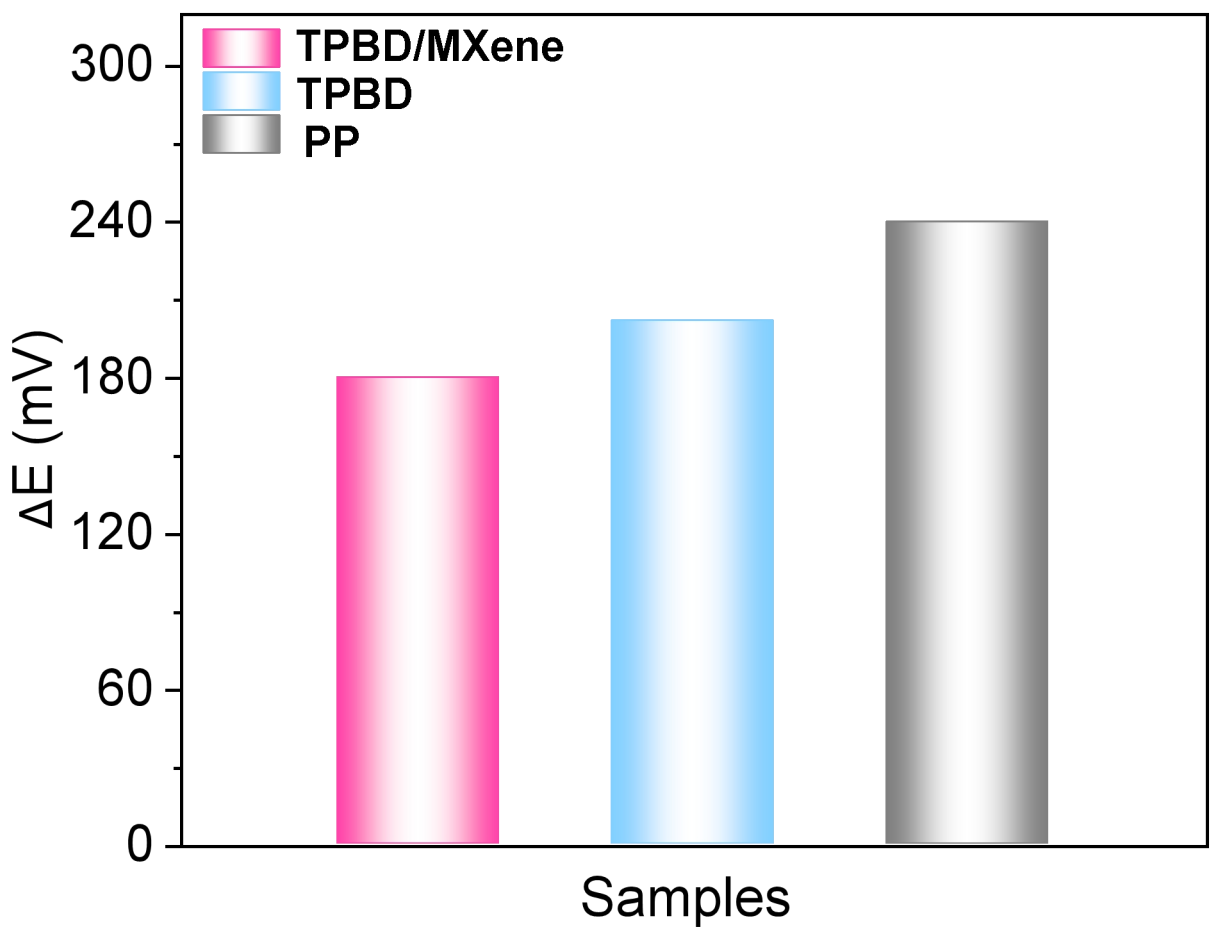

Fig. S43. ΔE values derived from GCD curves. The ΔE value derived from the analysis of the galvanostatic charge-discharge curves of TPBD/MXene, TPBD, and PP.

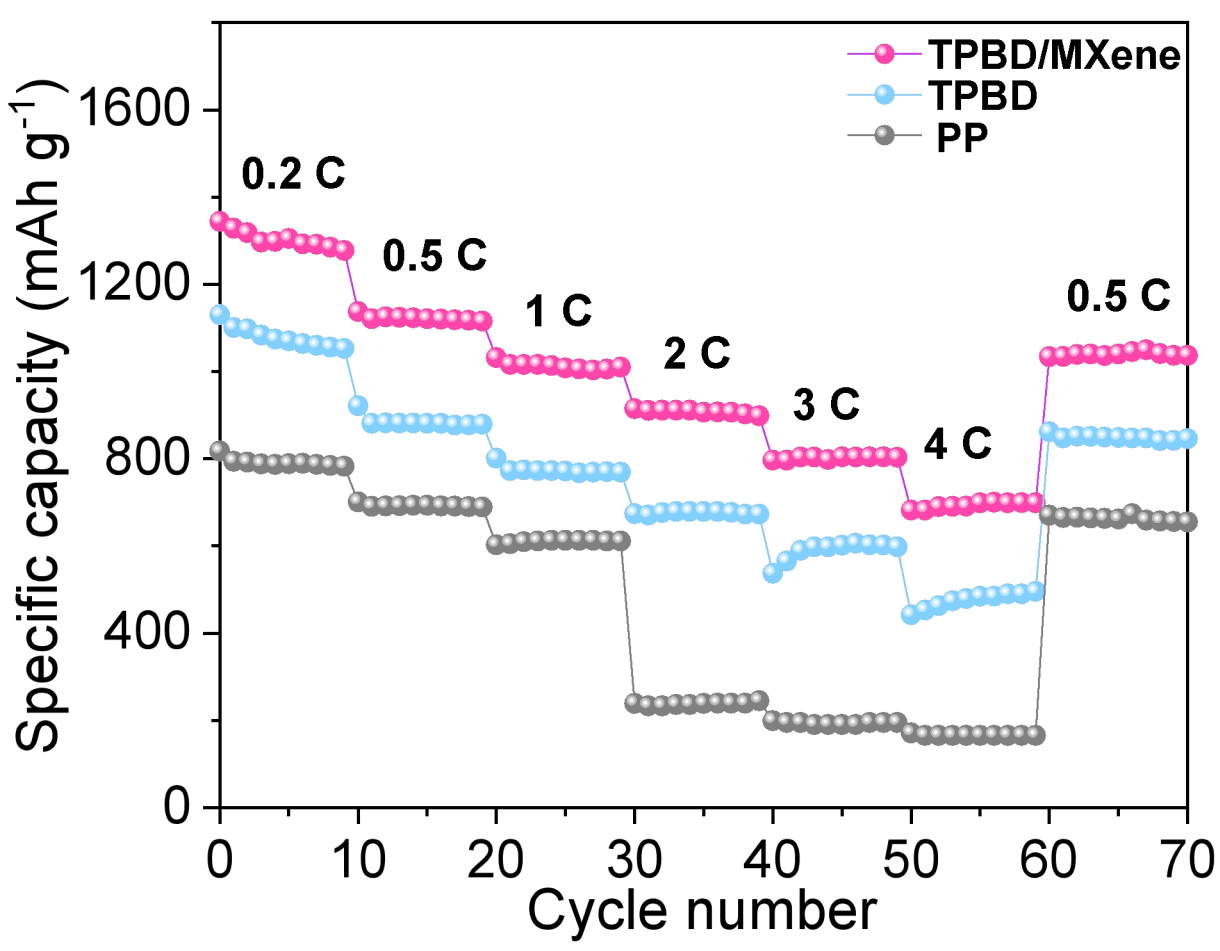

Fig. S44. Rate capability comparison of three separators. Rate capability of batteries employing TPBD/MXene, TPBD, and PP separators.

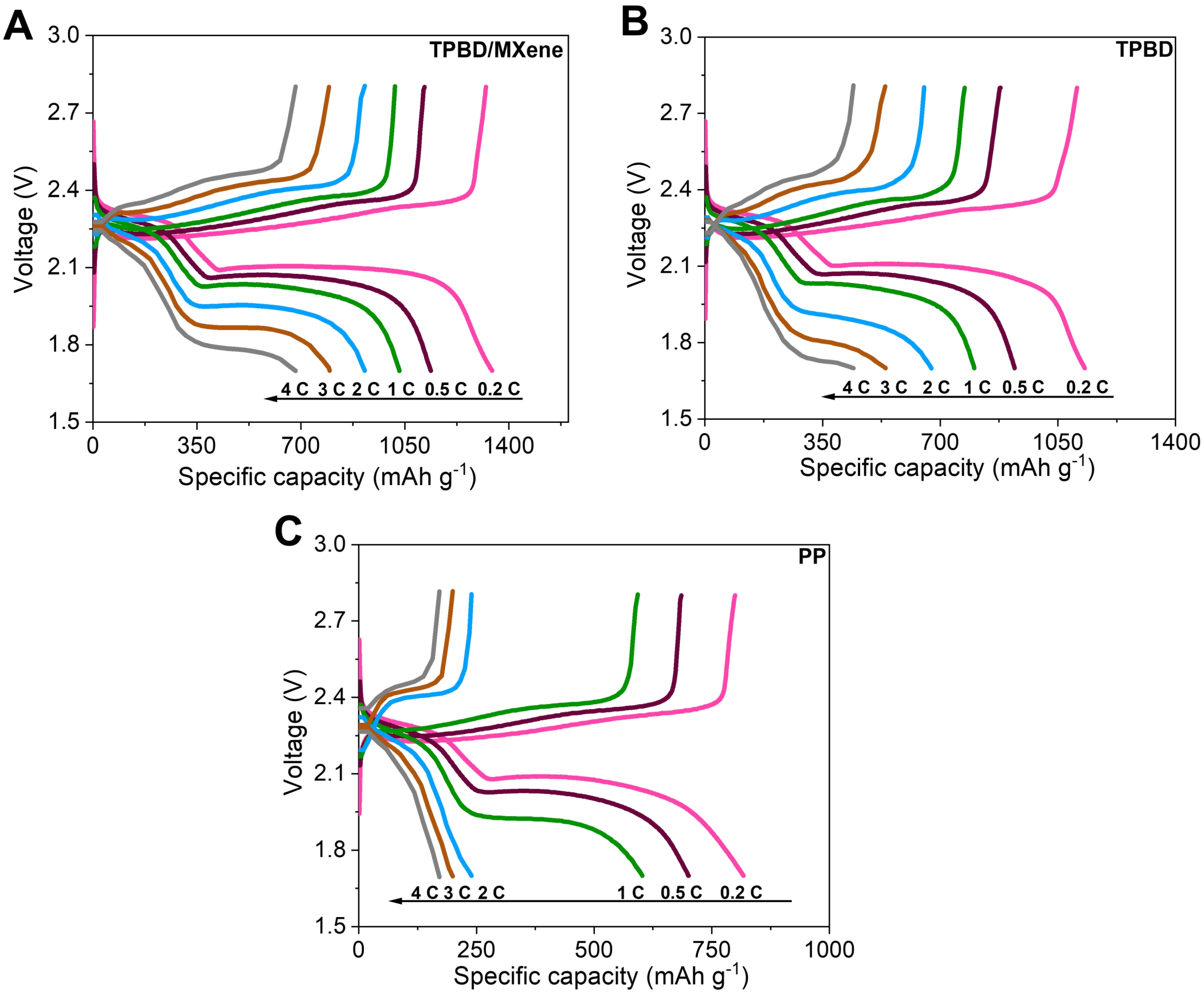

Fig. S45. Charge-discharge curves of three separators under various current rates.

Galvanostatic charge-discharge profiles at various current rates of batteries employing **(A)** TPBD/MXene, **(B)** TPBD, and **(C)** PP separators.

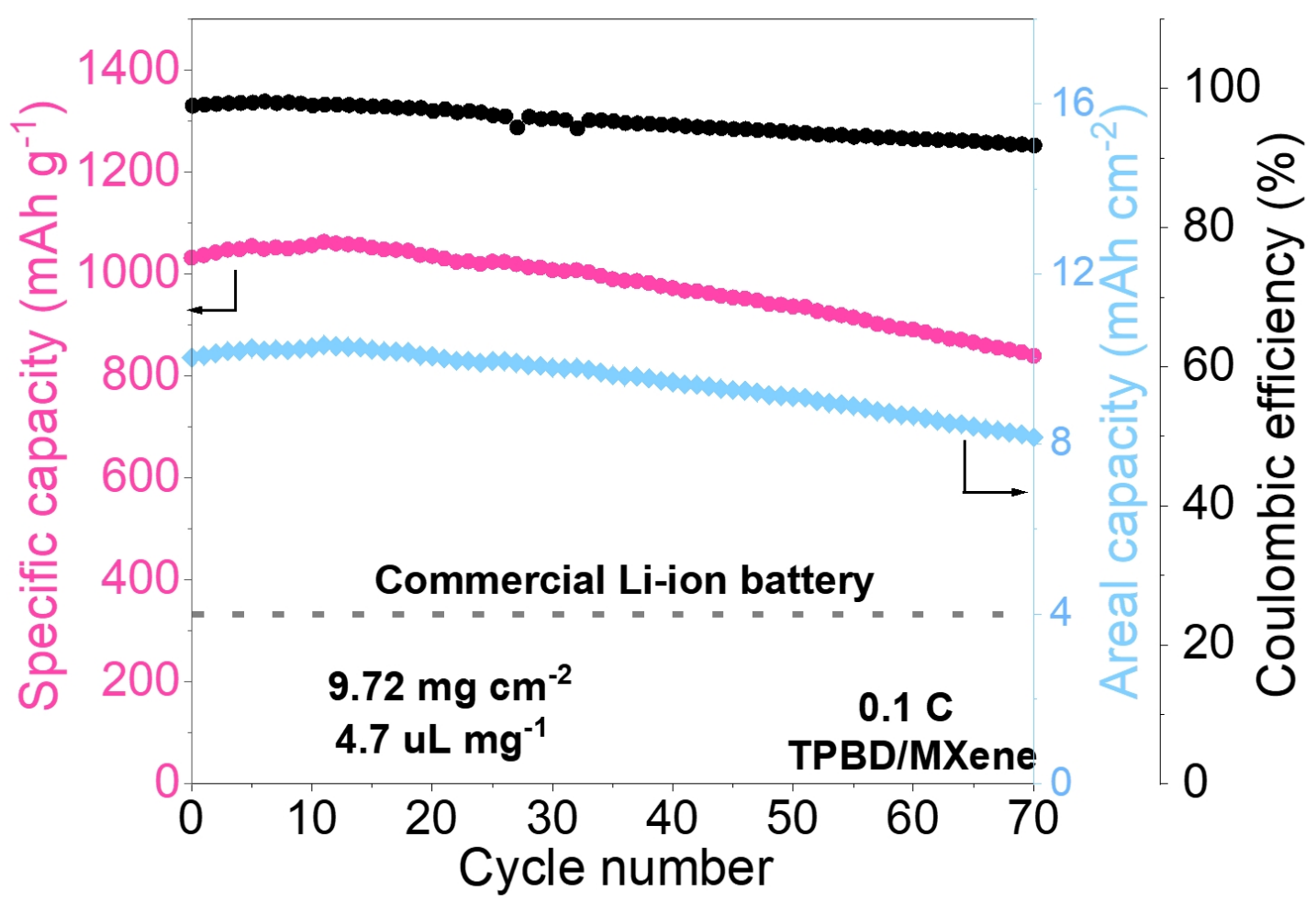

Fig. S46. Cycling behavior with 9.72 mg cm^{-2} sulfur loading.

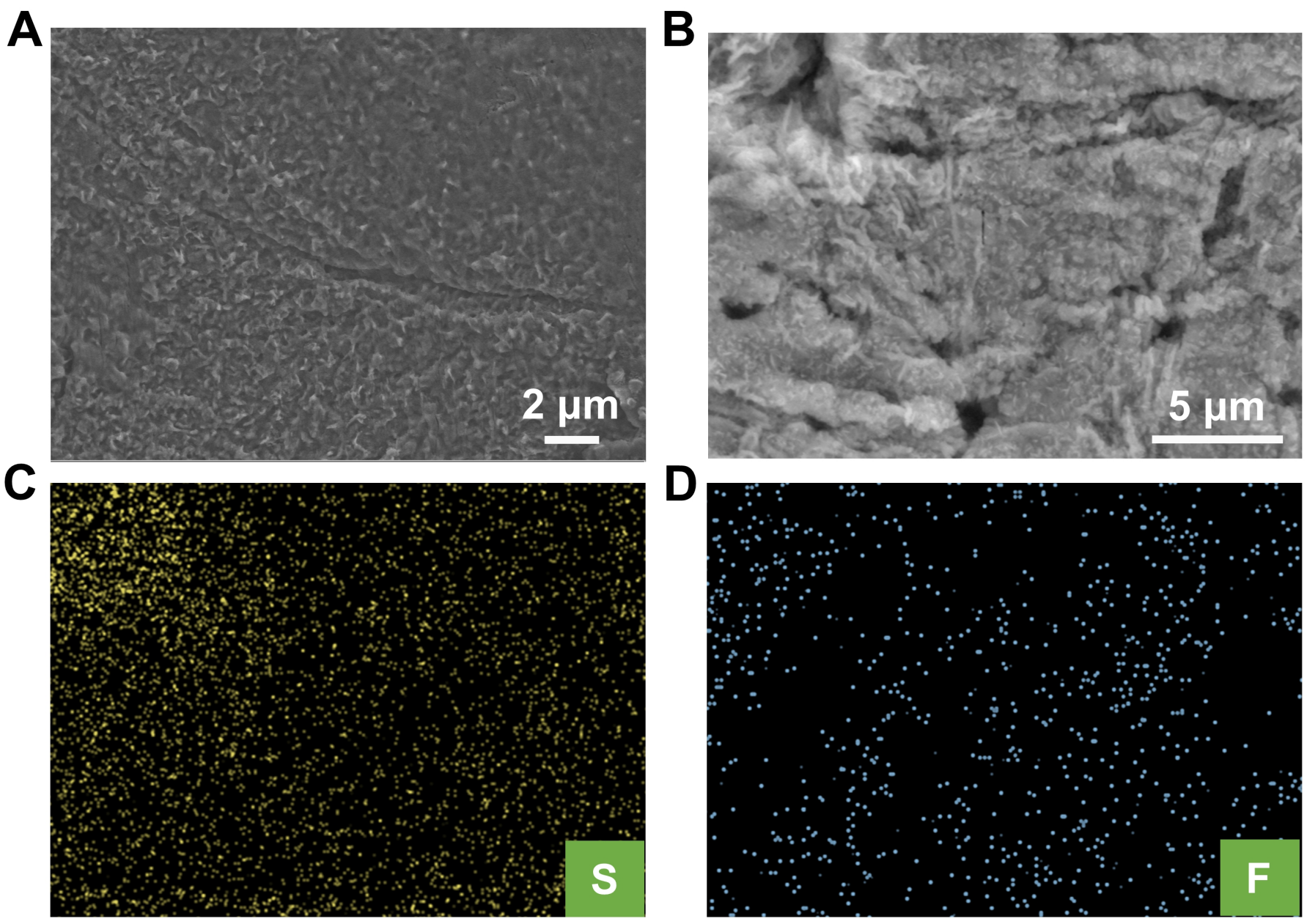

Fig. S47. SEM and EDS characterization. (A) SEM image of the Li anode before failure. **(B-D)** EDS elemental maps of S and F on the Li anode surface after cell failure.

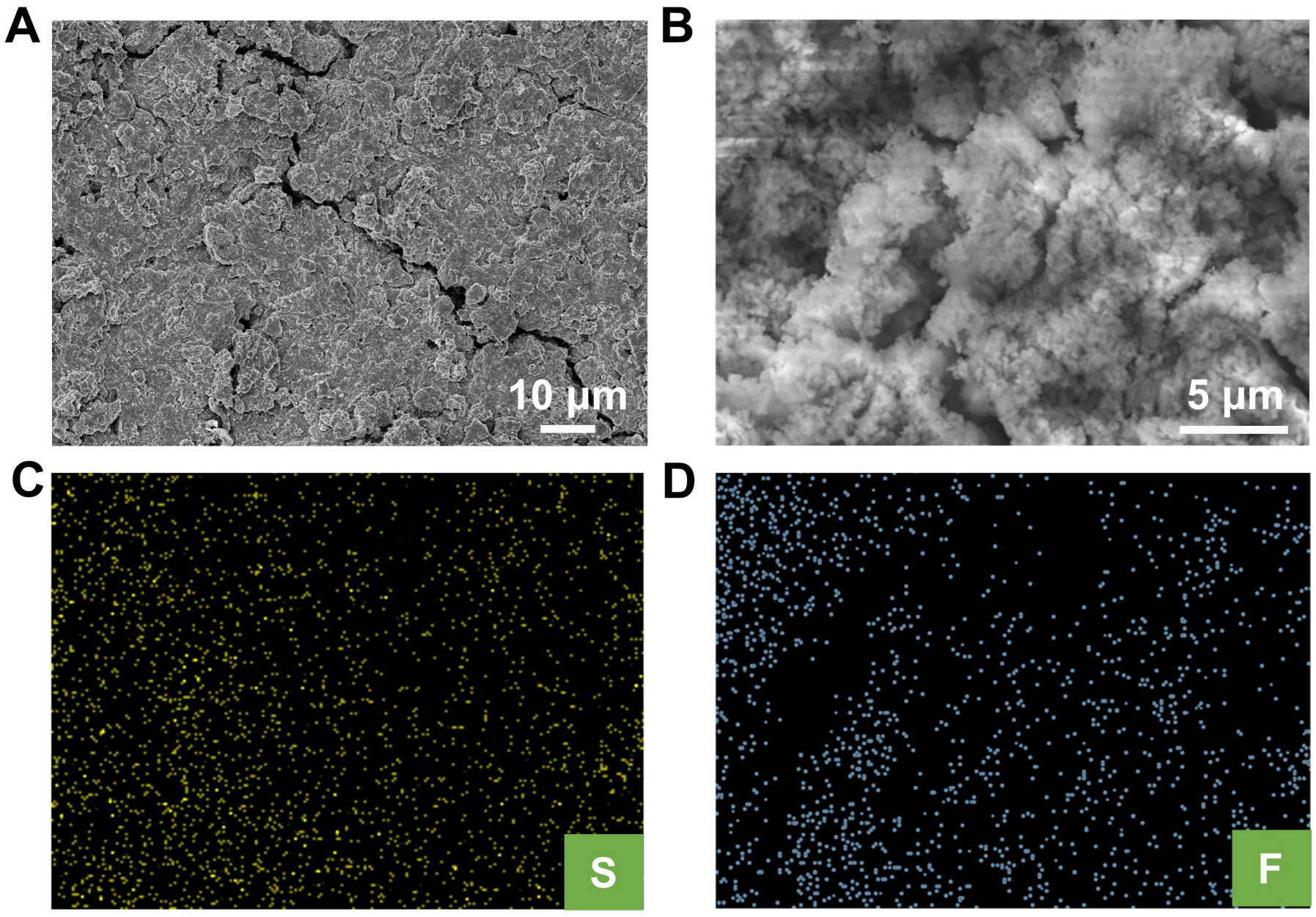

Fig. S48. SEM and EDS characterization. (A) SEM image of the TPBD/MXene separator before failure. **(B-D)** EDS elemental maps of S and F on the separator after cell failure.

Table S1. Summary of lithium-ion diffusion coefficient for TPBD/MXene, TPBD, and PP separators.

Modified separators	PP	TPBD	TPBD/MXene
D_{Li^+} at peak C ($cm^2\ s^{-1}$)	4.400×10^{-8}	2.453×10^{-7}	4.569×10^{-7}
D_{Li^+} at peak B (cm^2 s-1)	1.411×10^{-8}	4.439×10^{-8}	1.258×10^{-7}
D_{Li^+} at peak A (cm^2 s-1)	7.126×10^{-9}	3.661×10^{-8}	7.865×10^{-8}

Table S2. Comparative analysis of TPBD/MXene's long-circulating performance with latest reported research findings.

Separators	Coating loading (mg cm^{-2})	Sulfur area loading	Rate capacity (mAh g^{-1})/C rate	Capacity decay rate/Cycle number /C rate	Ref.
$NiSe_2/CoSe_2$-G/S	N/a	1	902/1 C	0.087 %/500/1C	(*63*)
D-VS_2	0.5	1.2	911.5/1 C	0.07%/1000/1 C	(*64*)
MoO_2–Mo_2N	0.51	0.8–1	732.5/1 C	0.028%/ 500/1 C	(*65*)
InC-HCTB	N/a	1.15	947.6/1 C	0.0436%/800/1 C	(*66*)
A/R-TiO_2	0.23	1.4	920/1 C	0.055%/800/1 C	(*67*)
L3DCO/3PS	0.49	1.4	1188/1 C	0.1%/300/1 C	(*68*)
N, S–Mo_2C/C-ACF	0.5	0.9-1.3	900/1 C	0.08%/600/1 C	(69)
**TPBD/MXene**	**0.28**	**1.87**	**1070.9/1 C**	**0.02%/1000/1 C**	**This work**

Table S3. Summary of key electrochemical metrics under various operational conditions.

Test Condition	Sulfur Loading (mg cm^{-2})	E/S (μL mg^{-1})	Temperature (°C)	Initial Capacity	Cycle number	C rate
Coin cell (Low loading)	2	20	25	1070.9 mAh g^{-1}	1000	1 C
Coin cell (High loading)	9.72	4.7	25	10.03 mAh cm^{-2}	70	0.1 C
Coin cell (Low Temp)	8.36	6.5	0	7.93 mAh cm^{-2}	259	0.1 C
Coin cell (High Temp)	7.51	6.2	50	1205.4 mAh g^{-1}	80	0.05 C
Pouch cell	5	5	25	1216.6 mAh g^{-1}	100	0.1 C

REFERENCES

1. S. Kim, W.-G. Lim, H. Jung, Y. C. Jeong, C.-Y. Park, S. B. Yang, C. H. Lee, D. Wang, K. Sohn, J. W. Han, J. Lee, Protective catalytic layer powering activity and stability of electrocatalyst for high-energy lithium-sulfur pouch cell. *Nat. Commun.* **16**, 1649 (2025).

2. H. Chen, Y. Qiu, Z. Cai, W. Liang, L. Liu, M. Li, X. Hou, F. Chen, X. Zhou, T. Cheng, L. He, J. Wang, X. Zhang, S. Dou, L. Li, Topological Insulator Heterojunction with Electric Dipole Domain to Boost Polysulfide Conversion in Lithium-Sulfur Batteries. *Angew. Chem. Int. Ed. Engl.* **64**, e202423357 (2025).

3. Y. He, D. Xiong, M. Chen, W. Zhang, S. Liu, Y. Ye, M. Wang, Y. Chen, Q. Tang, X. Peng, C. Wang, H. Zhan, H. Liu, M. Liu, J. Su, H. Shu, J. Wang, X. Wang, Modulating Ion-Dipole and Dipole–Dipole Interactions for Stable Wide-Temperature-Range Lithium–Sulfur Batteries Enabled by Quantum-Dot Catalysts. *Angew. Chem. Int. Ed. Engl.* **64**, e202512168 (2025).

4. T. Li, Y. Liu, J. Wang, H. Hao, Z. Yu, H. Liu, Constructing metal telluride-grafted MXene as electron "donor-acceptor" heterostructure for accelerating redox kinetics of high-efficiency Li-S batteries. *J. Mater. Chem. A* **12**, 12691–12701 (2024).

5. M. H. Parekh, H. Rao, D. Jokhakar, V. P. Parikh, M. Palanisamy, V. G. Pol, Polysulfide shuttle mitigation through a tailored separator for critical temperature energy-dense lithium–sulfur batteries. *Sustain. Energy Fuels* **6**, 5591–5599 (2022).

6. D. A. Gribble, P. F. Cheng, V. G. Pol, P. R. Kidambi, Size-selective nanoporous atomically thin graphene separators for lithium–sulfur batteries. *ACS Appl. Mater. Interfaces* **17**, 55554–55563 (2025).

7. K. Kim, P. J. Kim, J. P. Youngblood, V. G. Pol, Surface functionalization of carbon architecture with nano-MnO_2 for effective polysulfide confinement in lithium-sulfur batteries. *ChemSusChem* **11**, 2375–2381 (2018).

8. W. Wang, X. Wang, H. Yang, Z. Yu, W. Zhang, H. Huang, Z. Zhao, X. Zheng, L. Liu, Y. Deng, Y. Li, Design rules for anion-doped catalysts revealed by p-p-s orbital coupling in Li-S chemistry. *Nat. Commun.* **16**, 10895 (2025).

9. Y. Huang, J. Li, Y. Zhang, L. Lin, Z. Sun, G. Gao, B. Sa, L. Wang, L. Ma, S. Lee, M.-S. Wang, D.-L. Peng, K. Amine, Q. Xie, Energizing Robust Sulfur/Lithium Electrochemistry via Nanoscale-Asymmetric-Size Synergism. *J. Am. Chem. Soc.* **147**, 4752–4765 (2025).

10. T. Li, Y. Liu, J. Wang, H. Hao, Z. Yu, H. Liu, Electronic redistribution and fast delivery enabled by $Co_{0.5}Ni_{0.5}Te_2$ grafting 3D MXene heterostructure for accelerating High-Durability sulfur redox kinetics. *Chem. Eng. J.* **487**, 150502 (2024).

11. F. Li, H. Yuan, Y. Wang, Z. Xue, M. He, J. Wang, F. Wu, M. Huang, Y. Xiang, A. Hu, W. Chen, T. Wu, X. Sun, Tailoring Li-Accelerated Motif Enables Lithium Stabilization and Polysulfide Conversion for Long-Cycling Li–S Batteries. *Adv. Funct. Mater.* **35**, e11078 (2025).

12. T. Lai, K. Liao, A. Manthiram, Modulating Li^+ and Polysulfide Solvation with Low-Density Moderately Solvating Electrolytes for Lithium–Sulfur Batteries. *Angew. Chem. Int. Ed. Engl.* **65**, e22940 (2026).

13. J. Wang, J. Zhang, Y. Zhang, H. Li, P. Chen, C. You, M. Liu, H. Lin, S. Passerini, Atom-Level Tandem Catalysis in Lithium Metal Batteries. *Adv. Mater.* **36**, e2402792 (2024).

14. J. Zhang, F. Liu, R. He, Q. Guan, N. Tian, J. Wu, Z. Cao, S. Yin, Y. Zhang, L. Jia, X. Li, C. You, H. Liu, M. Liu, Y. Miao, H. Lin, J. Wang, Taming Interfacial Ion-Dipole Interactions With d-Orbital Delocalized Electron Catalysis Expediates Low-Temperature Li Metal Batteries. *Adv. Mater.* **38**, e10894 (2025).

15. M. He, L. Zhu, Y. Liu, Y. Jia, Y. Hao, G. Ye, X. Hong, Z. Xiao, Y. Ma, J. Chen, M. B. Shafqat, Q. Pang, Highly Solvating Electrolytes with Core-Shell Solvation Structure for Lean-Electrolyte Lithium-Sulfur Batteries. *Angew. Chem. Int. Ed. Engl.* **64**, e202415053 (2024).

16. L. Liao, F. Wang, J. He, L. Ma, Q. Liu, Y. Deng, Ultrathin TA-MXene Modification Layer with Sub-Nanometer Interlayer Channels Suppresses Polysulfide Shuttling and Accelerates Li^+ Desolvation. *Adv. Funct. Mater.*, **36**, e12612 (2025).

17. F. Zhu, J. Wang, Y. Zhang, H. Tu, X. Xia, J. Zhang, H. He, H. Lin, M. Liu, Low-Temperature Lithium Metal Batteries Achieved by Synergistically Enhanced Screening Li^+ Desolvation Kinetics. *Adv. Mater.* **37**, e2411601 (2024).

18. J. Wang, J. Zhang, X. Cheng, Y. Zhang, H. Li, Q. Guan, F. Wu, H. Li, D. Wang, M. Liu, Y. Zhang, Q. Xiao, S. Passerini, H. Lin, Electrode/Electrolyte Interface Studies of Rechargeable Li Batteries with Interface-Specific Sum Frequency Generation Spectroscopy. *J. Am. Chem. Soc.* **147**, 44633–44651 (2025).

19. W. Weng, V. G. Pol, K. Amine, Ultrasound Assisted Design of Sulfur/Carbon Cathodes with Partially Fluorinated Ether Electrolytes for Highly Efficient Li/S Batteries. *Adv. Mater.* **25**, 1608–1615 (2013).

20. B. C. Xiong, M. S. B. Reddy, S. Das, W. Stasko, G. Freeman, V. G. Pol, Suppressing Shuttle Effect through Solvent Coordination and Nitrate Synergy Enables Stable Lithium-Sulfur Batteries. *ACS Appl. Mater. Interfaces* **18**, 26105–26115 (2026).

21. Y. H. Zhuang, H. Yang, Y. H. Li, Y. Zhao, H. H. Min, S. Cui, X. D. Shen, H. Y. Chen, Y. F. Wang, J. Wang, Curvature-Induced Electron Delocalization Activates the Bifunctional Catalytic Activity of COF/MXene for High-Performance Lithium-Sulfur Batteries. *ACS Nano* **19**, 11058–11074 (2025).

22. K. Yang, F. Zhao, J. Li, H. Y. Yang, Y. C. Wang, Y. B. He, Designing Organic-Inorganic Hybrid Materials to Construct a Complementary Interface with Versatility for Li-S Batteries. *Adv. Funct. Mater.* **34**, 2410236 (2024).

23. C. L. Wei, Y. S. Wang, Y. C. Zhang, L. W. Tan, Y. Qian, Y. Tao, S. L. Xiong, J. K. Feng, Flexible and stable 3D lithium metal anodes based on self-standing MXene/COF frameworks for high-performance lithium-sulfur batteries. *Nano Res.* **14**, 3576–3584 (2021).

24. P. Dong, Y. Wang, A. Zhang, T. Cheng, X. Xi, J. Zhang, Platinum Single Atoms Anchored on a Covalent Organic Framework: Boosting Active Sites for Photocatalytic Hydrogen Evolution. *Acs Catalysis* **11**, 13266–13279 (2021).

25. T. Li, W. Liu, Y. Liu, J. Wang, H. Hao, Z. Yu, H. Liu, Covalent organic frameworks integrated MXene as selective "ion-sieving" heterostructure catalyst for kinetics-reinforced Li-S batteries. *Chem. Eng. J.* **498**, 155817 (2024).

26. Y. Lin, H. Cui, C. Liu, R. Li, S. Wang, G. Qu, Z. Wei, Y. Yang, Y. Wang, Z. Tang, H. Li, H. Zhang, C. Zhi, H. Lv, A covalent organic framework as a long-life and high-rate anode suitable for both aqueous acidic and alkaline batteries. *Angew. Chem. Int. Ed. Engl.* **62**, e202218745 (2023).

27. X. Yan, B. Wang, J. Ren, X. Long, D. Yang, An Unsaturated Bond Strategy to Regulate Active Centers of Metal-Free Covalent Organic Frameworks for Efficient Oxygen Reduction. *Angew. Chem. Int. Ed. Engl.* **61**, e202209583 (2022).

28. X. Kang, T. He, S. Niu, J. Zhang, R. Zou, F. Zhu, F. Ran, Precise Design of a 0.8 nm Pore Size in a Separator Interfacial Layer Inspired by a Sieving Effect toward Inhibiting Polysulfide Shuttling and Promoting Li^+ Diffusion. *Nano Lett.* **24**, 10007–10015 (2024).

29. X. Dong, W. Gu, X. Tong, G. Liu, J. Sun, H. Li, X. Gu, T. Zhu, S. Zhang, In situ growth strategy to construct "four-in-one" separators with functionalized polyphosphazene coatings for safe and stable lithium-sulfur batteries. *Small* **20**, e2311471 (2024).

30. Z. Ye, Y. Jiang, L. Li, F. Wu, R. Chen, Enhanced catalytic conversion of polysulfide using 1D CoTe and 2D MXene for heat-resistant and lean-electrolyte Li-S batteries. *Chem. Eng. J.* **430**, 132734 (2022).

31. X. Wang, D. Luo, J. Wang, Z. Sun, G. Cui, Y. Chen, T. Wang, L. Zheng, Y. Zhao, L. Shui, G. Zhou, K. Kempa, Y. Zhang, Z. Chen, Strain Engineering of a MXene/CNT Hierarchical Porous Hollow Microsphere Electrocatalyst for a High-Efficiency Lithium Polysulfide Conversion Process. *Angew. Chem. Int. Ed. Engl.* **60**, 2371–2378 (2021).

32. C. N. Zhu, C. C. Sun, R. H. Li, S. T. Weng, L. W. Fan, X. F. Wang, L. X. Chen, M. Noked, X. L. Fan, Anion-Diluent Pairing for Stable High-Energy Li Metal Batteries. *ACS Energy Lett.* **7**, 1338–1347 (2022).

33. Y. Jin, P. M. L. Le, P. Y. Gao, Y. B. Xu, B. W. Xiao, M. H. Engelhard, X. Cao, T. D. Vo, J. T. Hu, L. R. Zhong, B. E. Matthews, R. Yi, C. M. Wang, X. L. Li, J. Liu, J.-G. Zhang, Low-solvation electrolytes for high-voltage sodium-ion batteries. *Nat. Energy* **7**, 718–725 (2022).

34. X. Zuo, L. Wang, M. Zhen, T. You, D. Liu, Y. Zhang, Multifunctional TiN-MXene-Co@CNTs networks as sulfur/lithium host for high-areal-capacity lithium-sulfur batteries. *Angew. Chem. Int. Ed. Engl.* **63**, e202408026 (2024).

35. Z. Li, P. Li, X. Meng, Z. Lin, R. Wang, The Interfacial Electronic Engineering in Binary Sulfiphilic Cobalt Boride Heterostructure Nanosheets for Upgrading Energy Density and Longevity of Lithium-Sulfur Batteries. *Adv. Mater.* **33**, e2102338 (2021).

36. D. Yang, M. Li, X. Zheng, X. Han, C. Zhang, J. Jacas Biendicho, J. Llorca, J. Wang, H. Hao, J. Li, G. Henkelman, J. Arbiol, J. Ramon Morante, D. Mitlin, S. Chou, A. Cabot, Phase engineering of defective copper selenide toward robust lithium-sulfur batteries. *ACS Nano* **16**, 11102–11114 (2022).

37. Z. Jin, T. Lin, H. Jia, B. Liu, Q. Zhang, L. Li, L. Zhang, Z.-M. Su, C. Wang, Expediting the Conversion of Li_2S_2 to Li_2S Enables High-Performance Li-S Batteries. *ACS Nano* **15**, 7318–7327 (2021).

38. M. Zhen, X. Wang, Q. Yang, Z. Zhang, Z. Hu, Z. Li, Z. Wang, Nitrogen-Doped TiO_{2-x}(B)/MXene Heterostructures for Expediting Sulfur Redox Kinetics and Suppressing Lithium Dendrites. *Adv. Sci.* **11**, e2406475 (2024).

39. W. Cai, G. Li, K. Zhang, G. Xiao, C. Wang, K. Ye, Z. Chen, Y. Zhu, Y. Qian, Conductive Nanocrystalline Niobium Carbide as High-Efficiency Polysulfides Tamer for Lithium-Sulfur Batteries. *Adv. Funct. Mater.* **28**, 1704865 (2017).

40. Z. Li, F. Zhang, T. Cao, L. Tang, Q. Xu, H. Liu, Y. Wang, Highly stable lithium-sulfur batteries achieved by a SnS/porous carbon nanosheet architecture modified celgard separator. *Adv. Funct. Mater.* **30**, 2006297 (2020).

41. B. Wang, Y. Ren, Y. Zhu, S. Chen, S. Chang, X. Zhou, P. Wang, H. Sun, X. Meng, S. Tang, Construction of Co_3O_4/ZnO heterojunctions in hollow N-Doped carbon nanocages as microreactors for lithium-sulfur full batteries. *Adv. Sci.* **10**, e2300860 (2023).

42. C. Dong, C. Ma, C. Zhou, Y. Yu, J. Wang, K. Yu, C. Shen, J. Gu, K. Yan, A. Zheng, M. Gong, X. Xu, L. Mai, Engineering d-p Orbital Hybridization with P, S Co-Coordination Asymmetric Configuration of Single Atoms Toward High-Rate and Long-Cycling Lithium-Sulfur Battery. *Adv. Mater.* **36**, e2407070 (2024).

43. J. Feng, C. Zhang, W. Liu, S. Yu, L. Wang, T. Wang, C. Shi, X. Zhao, S. Chen, S. Chou, J. Song, Enabling Efficient Anchoring-Conversion Interface by Fabricating Double-Layer Functionalized Separator for Suppressing Shuttle Effect. *Angew. Chem. Int. Ed. Engl.* **63**, e202407042 (2024).

44. Z. Li, Y. Zhou, Y. Wang, Y.-C. Lu, Solvent-Mediated Li_2S Electrodeposition: A Critical Manipulator in Lithium-Sulfur Batteries. *Adv. Energy Mater.* **9**, 1802207 (2019).

45. F. Y. Fan, W. C. Carter, Y.-M. Chiang, Mechanism and Kinetics of Li_2S Precipitation in Lithium-Sulfur Batteries. *Adv. Mater.* **27**, 5203–5209 (2015).

46. K. Yang, F. Zhao, J. Li, H. Yang, Y. Wang, Y. He, Designing Organic-Inorganic Hybrid Materials to Construct a Complementary Interface with Versatility for Li-S Batteries. *Adv. Funct. Mater.* **34**, 2410236 (2024).

47. Q. Liu, X. Han, Z. Zheng, P. Xiong, R.-G. Jeong, G. Kim, H. Park, J. Kim, B.-K. Kim, H. S. Park, Crystallinity Regulated Functional Separator Based on Bimetallic Ni_xFe_y Alloy Nanoparticles for Facilitated Redox Kinetics of Lithium-Sulfur Batteries. *Adv. Funct. Mater.* **32**, 2207094 (2022).

48. T. Li, H. Hao, J. Wang, Z. Yuan, Z. Yu, H. Liu, Engineering covalent organic frameworks to enhance interfacial lithium-ion flux redistribution in dendrite-free lithium-sulfur batteries. *J. Mater. Chem. A* **13**, 7939–7948 (2025).

49. T. Li, C. Yang, H. Hao, Z. Yuan, Z. Yu, H. Liu, MXene porous engineering as lithium-ion kinetic pump for durable lithium-sulfur batteries. *J. Alloys Compd.* **1022**, 180032 (2025).

50. S. Chen, J. Luo, N. Li, X. Han, J. Wang, Q. Deng, Z. Zeng, S. Deng, Multifunctional LDH/Co_9S_8 heterostructure nanocages as high-performance lithium-sulfur battery cathodes with ultralong lifespan. *Energy Storage Mater.* **30**, 187–195 (2020).

51. M. Zheng, C. Guo, Z. Luo, J. Wu, X. Tang, L. Li, Q. Sun, Q. Ouyang, B. Shi, H. Nie, J.-J. Shao, G. Zhou, Molybdenum disulfide (MoS_2)/porous silica nanosheet composite barrier for polysulfide shuttling inhibition in lithium-sulfur batteries. *Compos. B: Eng.* **264**, 110898 (2023).

52. J. Xu, S. An, X. Song, Y. Cao, N. Wang, X. Qiu, Y. Zhang, J. Chen, X. Duan, J. Huang, W. Li, Y. Wang, Towards High Performance Li-S Batteries via Sulfonate-Rich COF-Modified Separator. *Adv. Mater.* **33**, e2105178 (2021).

53. B. Zhang, C. Luo, G. Zhou, Z.-Z. Pan, J. Ma, H. Nishihara, Y.-B. He, F. Kang, W. Lv, Q.-H. Yang, Lamellar MXene Composite Aerogels with Sandwiched Carbon Nanotubes Enable Stable Lithium-Sulfur Batteries with a High Sulfur Loading. *Adv. Funct. Mater.* **31**, 2100793 (2021).

54. Y. Zhang, J. Liu, J. Wang, Y. Zhao, D. Luo, A. Yu, X. Wang, Z. Chen, Engineering oversaturated Fe-N_5 multifunctional catalytic sites for durable lithium-sulfur batteries. *Angew. Chem. Int. Ed. Engl.* **60**, 26622–26629 (2021).

55. L. Zuo, Q. Ma, P. Xiao, Q. Guo, W. Xie, D. Lu, X. Yun, C. Zheng, Y. Chen, Upgrading the Separators Integrated with Desolvation and Selective Deposition toward the Stable Lithium Metal Batteries. *Adv. Mater.* **36**, e2311529 (2024).

56. H. Tang, N. Hu, L. Ma, H. Weng, D. Huang, J. Zhu, H. Yang, Z. Chen, X. Yin, J. Xu, H. He, Interfacial Dual-Modulation via Cationic Electrostatic Shielding and Anionic Preferential

Adsorption toward Planar and Reversible Zinc Electrodeposition. *Adv. Funct. Mater.* **34**, 2402484 (2024).

57. Y. Chen, K. Zou, X. Dai, H. Bai, S. Zhang, T. Zhou, C. Li, Y. Liu, W. K. Pang, Z. Guo, Polysulfide filter and dendrite inhibitor: highly graphitized wood framework inhibits polysulfide shuttle and lithium dendrites in Li-S batteries. *Adv. Funct. Mater.* **31**, 2102458 (2021).

58. X. Dong, W. Gu, X. Tong, G. Liu, J. Sun, H. Li, X. Gu, T. Zhu, S. Zhang, In Situ Growth Strategy to Construct "Four-In-One" Separators with Functionalized Polyphosphazene Coatings for Safe and Stable Lithium-Sulfur Batteries. *Small* **20**, e2311471 (2024).

59. J. Xu, A. Zhu, Z. Zheng, Y. Qi, Y. Cheng, Y. Cao, B. Peng, L. Ma, Y. Wang, Building Li-S batteries with enhanced temperature adaptability via a redox-active COF-based barrier-trapping electrocatalyst. *J. Energy Chem.* **101**, 702–712 (2025).

60. C.-X. Bi, N. Yao, X.-Y. Li, Q.-K. Zhang, X. Chen, X.-Q. Zhang, B.-Q. Li, J.-Q. Huang, Unveiling the Reaction Mystery Between Lithium Polysulfides and Lithium Metal Anode in Lithium-Sulfur Batteries. *Adv. Mater.* **36**, e2411197 (2024).

61. Y. Zhang, C. Kang, W. Zhao, Y. Song, J. Zhu, H. Huo, Y. Ma, C. Du, P. Zuo, S. Lou, G. Yin, D-P hybridization-induced "trapping-coupling-conversion" enables high-efficiency Nb single-atom catalysis for Li-S batteries. *J. Am. Chem. Soc.* **145**, 1728–1739 (2023).

62. J. Xu, L. Xu, Z. Zhang, B. Sun, Y. Jin, Q. Jin, H. Liu, G. Wang, Heterostructure ZnSe-$CoSe_2$ embedded with yolk-shell conductive dodecahedral as two-in-one hosts for cathode and anode protection of lithium-sulfur full batteries. *Energy Storage Mater.* **47**, 223–234 (2022).

63. H. Wang, H. Guo, Z. Huang, W. Liu, M. Li, J. Yao, J. Cui, Y. Wang, M. Ren, Bidirectional enhancement of Li_2S redox reaction by $NiSe_2$/$CoSe_2$-rGO heterostructured bi-functional catalysts. *J. Colloid Interface Sci.* **660**, 458–468 (2024).

64. G. Liu, Q. Zeng, Z. Fan, S. Tian, X. Li, X. Lv, W. Zhang, K. Tao, E. Xie, Z. Zhang, Boosting sulfur catalytic kinetics by defect engineering of vanadium disulfide for high-performance lithium-sulfur batteries. *Chem. Eng. J.* **448**, 137683 (2022).

65. J.-L. Yang, S.-X. Zhao, Y.-M. Lu, X.-T. Zeng, W. Lv, G.-Z. Cao, In-situ topochemical nitridation derivative MoO_2–Mo_2N binary nanobelts as multifunctional interlayer for fast-kinetic Li-Sulfur batteries. *Nano Energy* **68**, 104356 (2020).

66. X. Dong, W. Gu, X. Tong, G. Liu, J. Sun, H. Li, X. Gu, T. Zhu, S. Zhang, In Situ Growth Strategy to Construct "Four-In-One" Separators with Functionalized Polyphosphazene Coatings for Safe and Stable Lithium-Sulfur Batteries. *Small* **20**, e2311471 (2024).

67. L. Ma, Y. Zhang, S. Zhang, L. Wang, C. Zhang, Y. Chen, Q. Wu, L. Chen, L. Zhou, W. Wei, Integrating Energy Band Alignment and Oxygen Vacancies Engineering of TiO_2 Anatase/Rutile Homojunction for Kinetics-Enhanced Li-S Batteries. *Adv. Funct. Mater.* **33**, 2305788 (2023).

68. L. Wu, C. Cai, X. Yu, Z. Chen, Y. Hu, F. Yu, S. Zhai, T. Mei, L. Yu, X. Wang, Scalable 3D Honeycombed Co_3O_4 Modified Separators as Polysulfides Barriers for High-Performance Li–S Batteries. *ACS Appl. Mater. Interfaces* **14**, 35894–35904 (2022).

69. H. Li, Q. Jin, D. Li, X. Huan, Y. Liu, G. Feng, J. Zhao, W. Yang, Z. Wu, B. Zhong, X. Guo, B. Wang, Mo_2C-Embedded Carambola-like N, S-Rich Carbon Framework as the Interlayer Material for High-Rate Lithium–Sulfur Batteries in a Wide Temperature Range. *ACS Appl. Mater. Interfaces* **12**, 22971–22980 (2020).
